# Replay in the human visual cortex during brief task pauses is linked to implicit learning of successor representations

**DOI:** 10.1073/pnas.2507516122

**Published:** 2025-08-22

**Authors:** Lennart Wittkuhn, Lena M. Krippner, Christoph Koch, Nicolas W. Schuck

**Affiliations:** ^a^Department of Cognitive Neuroscience for Learning and Change, Institute of Psychology, University of Hamburg, Hamburg 20146, Germany; ^b^Max Planck Research Group NeuroCode, Max Planck Institute for Human Development, Berlin 14195, Germany; ^c^Max Planck UCL Centre for Computational Psychiatry and Ageing Research, Berlin 14195, Germany; ^d^Harding Center for Risk Literacy, University of Potsdam, Potsdam 14482, Germany

**Keywords:** replay, implicit learning, fMRI

## Abstract

Humans can implicitly learn about multistep sequential relationships between events in the environment from their statistical co-occurrence. Theoretical work has suggested that neural replay is a candidate mechanism that aids such learning. Here, we used functional MRI (fMRI) to test whether replay is related to implicit learning of higher-order sequential relationships. Human participants viewed sequences of images that followed probabilistic transitions determined by ring-like graph structures. Behavioral modeling of response times revealed that participants acquired multistep transition knowledge in a manner consistent with gradual updating of an internal successor representation (SR) model. Yet, half of participants did not report being aware of any sequential task structure, and most participants failed to provide meaningful transition probability ratings in a posttask test. Analyses of temporal dynamics of multivariate fMRI patterns during brief 10 s pauses from the ongoing statistical learning task indicated backward sequential replay of multistep sequences in visual cortical areas. Variations in model parameters between participants that captured response time patterns related to strength of neural replay. No corresponding relations between replay and measures of explicit awareness were found. These findings indicate that implicit learning of higher-order relationships establishes an internal SR-based map of the task and is accompanied by cortical on-task replay.

## Significance

Brain activity reflects more than ongoing perception and action, it also recapitulates past events during sleep and rest. This “neural replay” plays a key role in learning and memory and has been studied extensively in the rodent hippocampus. Using functional MRI analysis techniques, we ask which relation replay outside of the hippocampus has to human cognition, in particular statistical learning which occurs sometimes with and sometimes without explicit awareness. Focusing on very brief 10 s pauses during an ongoing task, we find that replay occurs in the visual cortex and is related to predictive sequential learning, but not to explicit sequence knowledge. This sheds light on how the human brain builds implicit predictive representations and the nature of neural replay.

Abstract structural knowledge that generalizes specific experiences in the form of a so-called cognitive map is thought to provide the basis for flexible learning, inference, and generalization (1–3). Previous studies have shown that neural replay—the sequential reactivation of neural patterns that reflects internal “offline” processing rather than external inputs (e.g., refs. 4 and 5)—is a mechanism that uses abstract knowledge to generate flexible behavior (6–10). While other research has suggested that replay could be also used to learn cognitive maps (7, 10), much less is known about whether, and in which context, this holds true in the human brain.

Here, we investigated whether neural replay plays a role in learning abstract task knowledge by extracting statistical regularities from individual events (11, 12). While most prior work on cognitive maps and replay has relied on tasks in which participants are explicitly instructed to follow sequential rules (e.g., refs. 6–8), natural learning commonly happens without instructions (12–14). Such “statistical” learning is automatic and incidental, i.e., it occurs without any premeditated intention to learn, and often leads to implicit knowledge that is not intentionally accessible (15–17). Many experiments have shown that humans can implicitly learn about higher-order relational structures among individual events that go beyond pairwise transition probabilities (e.g., refs. 18 and 19), including ordinal and hierarchical information that structures individual subsequences (20–23), graph topological aspects such as bottlenecks (11, 24, 25), and macroscale aspects of graph structures (26, 27).

One core benefit of abstracted knowledge of transition structures is that it facilitates planning multistep sequences (28, 29). While experienced transitions directly yield information about one-step probabilities between pairs of events, they can also be used to compute which events can be expected over a multistep future horizon, i.e. long-term visitation probabilities. This idea is formalized in the successor representation (SR) model (30), a predictive map that reflects the (discounted) expected visitations of future events, or states (12, 31–34), that can be learned online through a temporal difference (TD) learning mechanism that updates after each experience (30, 35). Based on this background, our study aimed to investigate whether implicit statistical learning leads to SR maps, which in turn supports mental simulation.

Neurally, our main interest was whether implicit learning of multistep knowledge involves replay, and if so, when and where this replay occurs. Theoretical work has proposed that replay can be used to learn SR representations (35), but empirical tests and knowledge about characteristics of SR-linked replay are lacking. While it is well established that replay in the hippocampus can reflect previously experienced transition structure (e.g., refs. 7, 36, and 37), some studies have found that visual cortical regions carry expectations about upcoming visual stimuli (38–41), and found anticipatory activation sequences following perceptual sequence learning (42–45). Concerning the idea that replay may specifically be linked to learning rather than using knowledge, it is interesting to observe that human as well as animal studies have found replay during very brief pauses from the ongoing task, a phenomenon known as “online” or “on-task” replay (6, 7, 46–49). The idea that neural replay during the task might support learning is in line with studies that have pointed out that short breaks of around 25 s between blocks in a motor learning task are linked to higher-order rule learning (50), variations in break duration can have effects on learning (51) and linked magnetoencephalography (MEG) markers of replay during 10 s breaks to behavioral gains (52). Hence, we tested whether online replay in visual cortical regions reflects learning of predictive knowledge upon which SR-based cognitive maps are based. Last, we addressed a question specifically about the human brain: Is replay related to whether knowledge becomes explicit or remains implicit?

To test these ideas, participants performed an incidental statistical learning paradigm (cf. 26, 53) in which visual presentation order and motor responses followed statistical regularities that were determined by ring-like graph structures. The nature of the graph structure allowed us to dissociate knowledge about individual transition probabilities from an SR-based cognitive map that entails long-term visitation probabilities. Moreover, the transition probabilities among the task stimuli changed halfway through the experiment without prior announcement, which allowed us to understand the dynamic updating of task knowledge and replay within the same participants. Finally, assessing knowledge in posttask questionnaires allowed us to investigate possible links between replay and implicit vs. explicit learning.

## Results

**Fig. 1.**
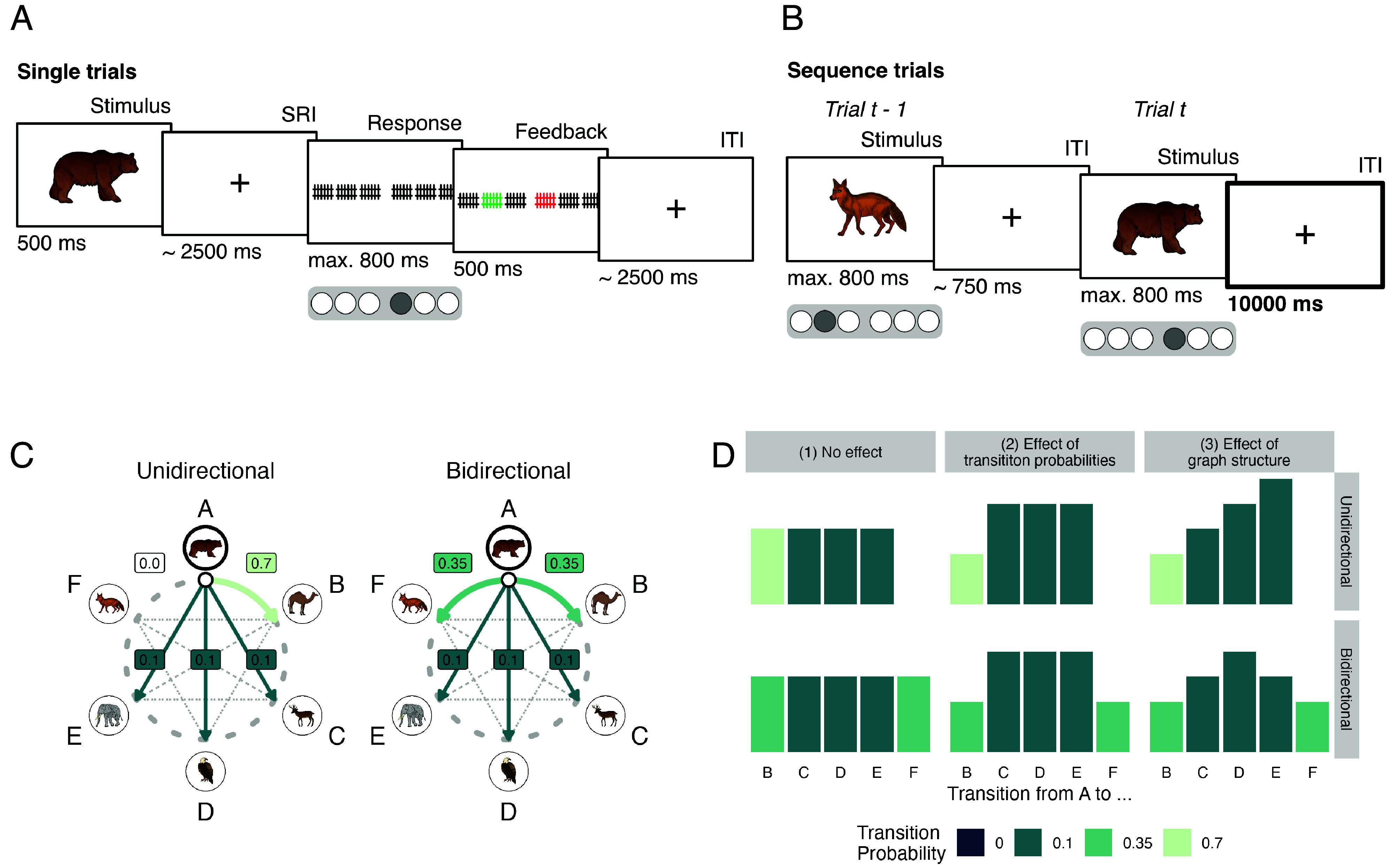
Task design. (*A*) On single trials image presentations were followed by response and feedback screens, separated by 2.5 s stimulus–response intervals (SRIs) and intertrial intervals (ITIs). (*B*) On sequence trials, images were separated by 750 ms in 90% of the trials, and by long 10 s ITIs in randomly selected 10% of cases (*interval trials*). (*C*) The unidirectional graph (*Left*) was characterized by frequent clockwise transitions ($P_{\mathrm{AB}}=0.7$), no counterclockwise transitions ($P_{\mathrm{AF}}=0$), and rare other transitions (P=0.1). In the bidirectional graph (*Right*), stimuli were equally likely to transition clockwise or counterclockwise ($P_{\mathrm{AB}}=P_{\mathrm{AF}}=0.35$). Other transitions remained the same. Transition probabilities are highlighted for node A only, but equally apply to all other nodes. (*D*) Illustration of surprise of different transitions assuming different knowledge. Columns reflect assumptions that participants have (*Left*) no knowledge, (*Middle*) one-step transition knowledge, or (*Right*) know the multistep “node distance.” Stimuli from ref. 55, courtesy of M. Tarr freely available at: https://sites.google.com/andrew.cmu.edu/tarrlab/stimuli under a CC BY-NC-SA 3.0 license. Drawings from ref. 54; see *SI Appendix*, Fig S2.

Thirty-nine human participants took part in an functional MRI (fMRI) experiment over two sessions (for details, see *Materials and Methods* and *SI Appendix*, Fig. S1). Participants were first informed that the experiment involved six images of animals (cf. 54, 55, *SI Appendix*, Fig. S2) and six response buttons mapped onto their index, middle, and ring fingers of both hands (*SI Appendix*, Fig. S3*B*). Participants then began the first session of MRI, during which they learned the stimulus–response (S-R) mappings between images and response buttons through feedback (*single trials*, Fig. 1*A*, 8 runs, 60 trials per run, 480 trials in Session 1). In single trials, images were shown without any particular sequential order, i.e., all pairwise sequential orderings of the images were presented equally often per run.

The second session started with one additional run of single trials that was followed by five runs of *sequence trials* (Fig. 1*B*, 5 runs, 240 trials per run, 1,200 trials in total). As before, participants had to press the correct button in response to each animal image. Participants were only informed that in this task condition, no feedback would be provided and images would be shown at a faster pace (800 ms per image and 750 ms intertrial interval (ITI) between images on average), with occasional 10 s breaks in between (10% of trials, i.e., 120 of all sequence trials per participant during which only a fixation cross was on the screen, henceforth *interval trials*; 24 trials per stimulus). Unbeknownst to participants, images also started to follow a probabilistic transition structure (see below and Fig. 1*C* and *SI Appendix*, Fig. S4*A*) in this phase, such that images as well as motor responses became sequentially structured and partially predictable.

The sequential ordering of images during sequence trials was determined by either a unidirectional or bidirectional ring-like graph structure with probabilistic transitions (Fig. 1*C* and *SI Appendix*, Fig. S4*A*; for details, see *Materials and Methods* and *SI Appendix*). In the unidirectional graph (Fig. 1*C*, *Left*, henceforth *uni*), each image had one frequent transition to the clockwise neighboring node (probability of $P_{\mathrm{ij}}=0.7$), never transitioned to the counterclockwise neighbor ($P_{\mathrm{ij}}=0.0$), and was followed occasionally by the three other nodes ($P_{\mathrm{ij}}=0.1$ each; *SI Appendix*, Fig. S4 *A*, *Left*). In consequence, stimuli most commonly transitioned in clockwise order along the ring shown in Fig. 1*C*. In the bidirectional graph (Fig. 1*C*, *Right*, henceforth *bi*), transitions to both neighboring nodes (clockwise and counterclockwise) were equally likely ($P_{\mathrm{ij}}=0.35$), and transitions to all other three nodes occurred with $P_{\mathrm{ij}}=0.1$ (*SI Appendix*, Fig. S4 *A*, *Right*), as in the unidirectional graph. Every participant started the task in one of these conditions (uni or bi). Halfway through the third run, transitions began to be governed by the alternative graph, such that all participants experienced both graphs as well as the change between them (12 participants started in the uni condition and transitioned to bi; 27 participants experienced the reverse order (*SI Appendix*, Fig. S4*B*). Sequence transitions were designed to ensure equal presentation of stimuli to prevent any bias in stimulus repetition (cf. 56). At the end of the second session, participants completed a posttask questionnaire assessing explicit sequence knowledge.

### Behavioral Results.

**Fig. 2.**
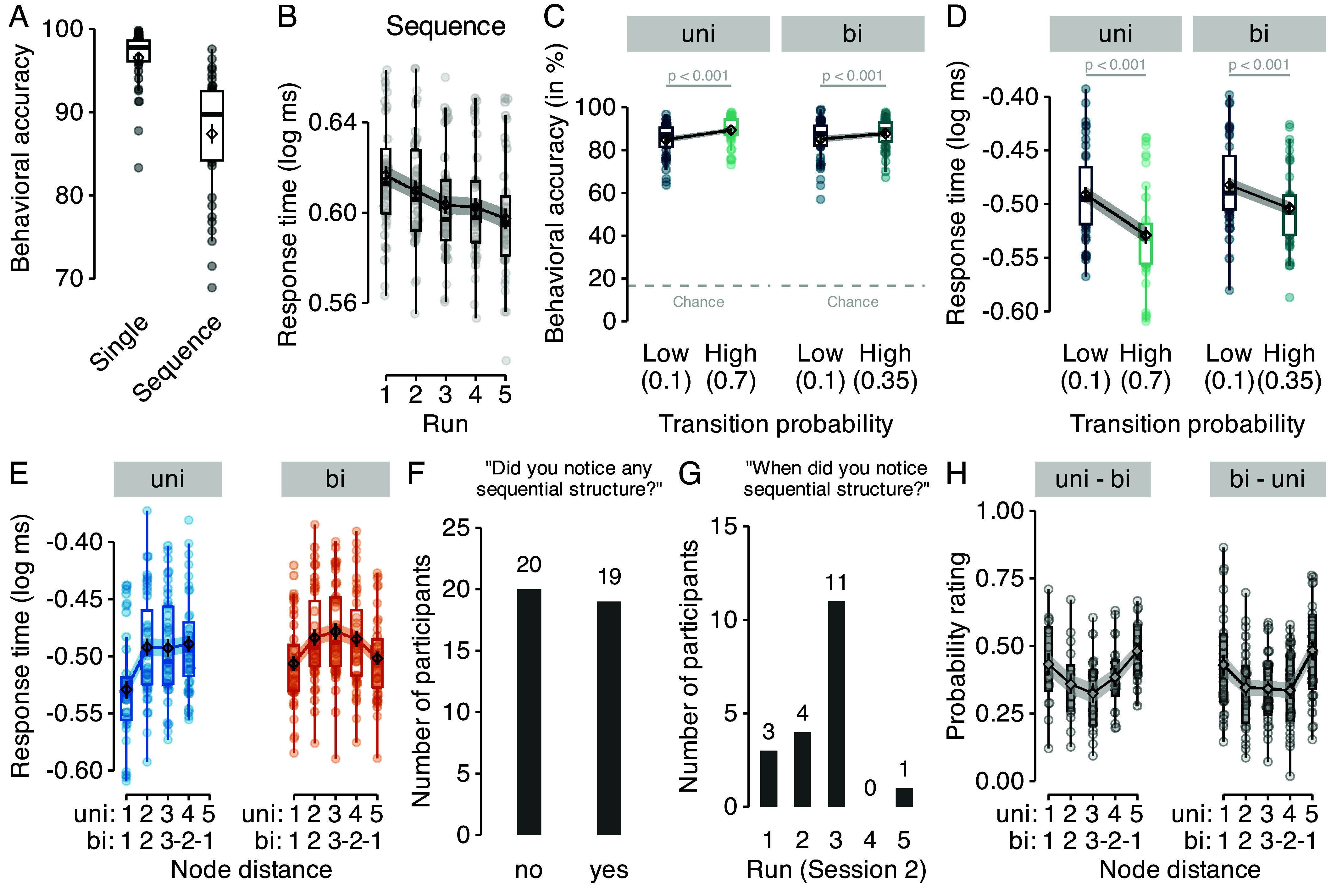
Behavior is influenced by multistep transition probabilities. (*A*) Mean behavioral accuracy (%) across all nine runs of single trials (*Left*) and across all five runs of the sequence trials (*Right*). (*B*) Mean log response time (ms) per run in sequence trials. (*C*) Mean behavioral accuracy following transitions with low and high probability for both graph structures (panels). Colors are as in Fig. 1 *C* and *D*. The horizontal dashed lines indicate the chance level (16.67%). (*D*) Mean log response time following transitions with low ($P_{\mathrm{ij}}=0.1$) and high probability ($P_{\mathrm{ij}}=0.7$ and $P_{\mathrm{ij}}=0.35$ in the uni- and bidirectional graph conditions of sequence trials, respectively) for both graph structures (panels). (*E*) Mean log response time as a function of uni- or bidirectional node distance (x-axis) in data from both graph structures (colors/panels). (*F*) Number of participants who reported noticing sequential structure in the task. (*G*) Distribution of when participants reported noticing sequential structure [only participants who answered yes in (*F*), n=19]. (*H*) Ratings of pairwise transition probabilities as a function of node distance, separately for both orders of graph structure (uni–bi vs. bi–uni; panels). Boxplots indicate the median and interquartile range (IQR), diamonds show sample mean, dots individual participants. Error bars and shaded areas indicate ±1 SEM.

Participants learned the stimulus–response (S-R) mappings between images and response buttons sufficiently well. Behavioral accuracy on single trials (Fig. 1*A*) surpassed the chance-level (16.67%) in all runs ($\overset{¯}{x}\geq 86.50$%, CIs [≥80.79, +∞], $t_{38}\geq 20.62$, Ps <0.001 (corrected), ds ≥3.30; Fig. 2*A* and *SI Appendix*, Fig. S5*A*). The same was true during sequence trials ($\overset{¯}{x}\geq 85.12$, CIs [≥82.55, +∞], $t_{38}\geq 44.90$, Ps <0.001 (corrected), ds ≥7.19; Figs. 1*B* and 2*A* and *SI Appendix*, Fig. S5*B*), where behavioral accuracy also improved with time (effect of run: $F_{1.00,38.00}=7.96$, P=0.008, *SI Appendix*, Fig. S5*B*).

Although participants were not informed that images during sequence trials followed a sequential structure, we expected that incidental learning would allow them to anticipate upcoming stimuli during these trials, and thus respond faster with learning. A linear mixed effects (LME) model that tested the effect of task run on response times was in line with this idea, showing a significant decrease of response times over the course of learning, $F_{1.00,38.00}=25.86$, P<0.001 (Fig. 2*B*). Consistent with the idea that participants learned about the probabilistic transition structure we also found that they responded faster and more accurately to one-step transitions with high compared to low probabilities in the unidirectional graph condition ($P_{\mathrm{ij}}=0.7$ (high) vs. $P_{\mathrm{ij}}=0.1$ (low) transition probabilities, Ps <0.001), and in the bidirectional graph condition ($P_{\mathrm{ij}}=0.35$ (high) vs. $P_{\mathrm{ij}}=0.1$ (low), all Ps <0.001 (corrected), ds ≥0.67; Fig. 2 *C* and *D*).

Our main behavioral hypothesis was that participants would not only learn about one-step transition probabilities but also form internal maps of the underlying graphs that reflect the higher-order structure of statistical multistep relationships between stimuli, i.e., how likely a particular stimulus will be experienced in two, three, or more steps (cf. 19, 26). In our task, this meant that participants might react differently to the three transitions that all have the same one-step transition probability, since they differ in how likely they would occur in multistep trajectories. For instance, the one-step transitions A→C, A→D, and A→E all had the same probabilities in the unidirectional graph ($P_{\mathrm{ij}}=0.1$; see Fig. 1*C*), but the two-step probability of A→C was higher than for the other transitions, since the most likely two-step path was A→B→C. This implies that participants should react faster to A→C than to A→D or A→E transitions. For simplicity, we will henceforth refer to the A→C transition as having a shorter “node distance,” than A→D or A→E (see the rightmost column in Fig. 1*D*, where colors reflect one-step transition probabilities, and the height of the bars indicate node distance). Analyzing response times as a function of the node distance (Fig. 1*D*; for details, see *Materials and Methods* and *SI Appendix*) indicated a significant effect of node distance on response times in both unidirectional, $F_{1.00,115.78}=44.34$, P<0.001, and bidirectional data, $F_{1.00,38.00}=57.36$, P<0.001 (Fig. 2*E*).

We assessed whether participants were able to express knowledge of the sequential ordering of stimuli and graph structures explicitly during a posttask questionnaire. Asked whether they had noticed any sequential ordering of the stimuli in the preceding sequence task, n=19 participants replied “yes” and n=20 replied “no” (Fig. 2*F*). Performance on the sequence task did not differ significantly between participants with and without explicit knowledge, as the effect of node distance on response times (see above) was comparable between these two groups (Ps ≥0.48 for each node distance). Of those participants who noticed sequential ordering (n=19), almost all (18 out of 19) indicated that they had noticed ordering within the first three runs of the task (Fig. 2*G*), and more than half of those participants (11 out of 19) indicated that they had noticed ordering in the third task run, during which the graph structure was changed. Thus, sequential ordering of task stimuli remained at least partially implicit in half of the sample, and the change in the sequential order halfway through the third run of graph trials seemed to be one potential cause for the realization of sequential structure.

The posttask questionnaire also asked participants to estimate the transition probabilities of all pairwise stimulus combinations (A→B, B→A, A→C, etc.; for details, see *SI Appendix*, *Methods*). On average, probability ratings reflected the bidirectional graph structure (Fig. 2*H*), i.e., probabilities of clockwise and counterclockwise neighboring transitions were rated higher than other transitions, independently of experienced graph order. A detailed analysis that compared the answers of each participant to a random guessing model (for details, *Materials and Methods* and *SI Appendix*) indicated that the ratings of n=26 (66%) subjects were indistinguishable from random (*SI Appendix*, Fig. S7). While these results did not match with the binary responses on a participant-by-participant basis (11 of 19 participants who answered “yes” to the first question provided seemingly random probability ratings), they supported the overall conclusion that learning was implicit in half or more of participants.

To investigate how multistep knowledge emerged from experience, we modeled response times using a SR model (30) that iteratively learns the discounted long-term occupation probabilities of every node starting from all other nodes. In this model, each node is associated with a vector that reflects the probability that starting from the current node a participant would experience any of the other nodes over a future-discounted predictive horizon. While these SR-vectors were uniformly initialized at the beginning of the task, they were dynamically updated following each transition experienced in the task, using a TD learning rule (30, 35). Concretely, after experiencing the transition from image $s_{t}$ to $s_{t+1}$, the row corresponding to image $s_{t}$ of the successor matrix M was updated as follows

[1] $$\begin{array}{c} M_{s_{t},*}=M_{s_{t},*}+\alpha \left[1_{s_{t+1}}+\gamma M_{s_{t+1},*}-M_{s_{t},*}\right], \end{array}$$

whereby $1_{s_{t+1}}$ is a one-hot vector with a 1 in the $s_{t+1}$th position, and α (“alpha”) is a learning rate. The discounting parameter γ (“gamma”) defines the extent to which multistep transitions are taken into account, which we will henceforth refer to as the “predictive horizon” (cf. 57, 58).

**Fig. 3.**
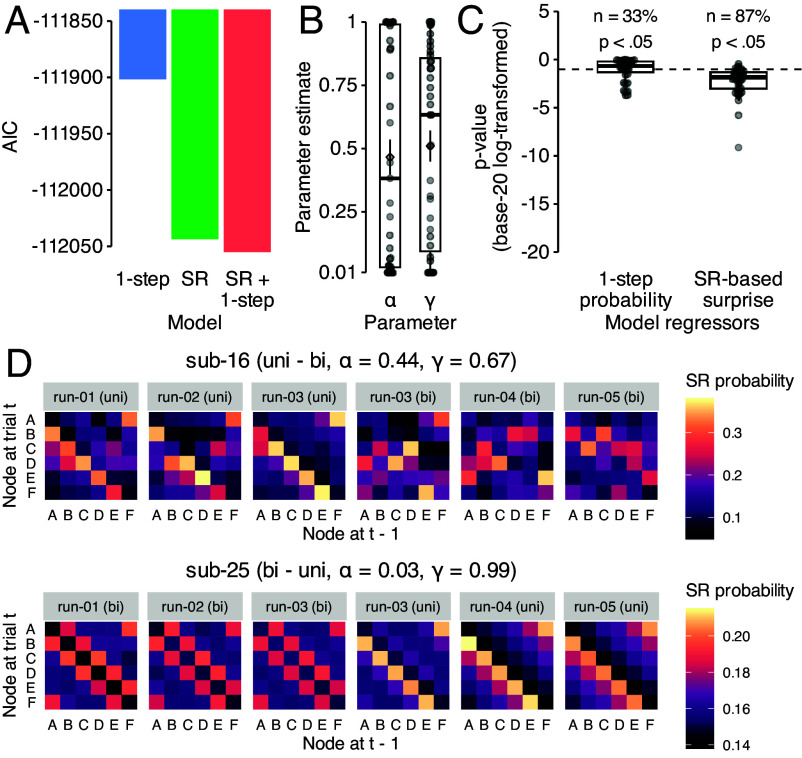
successor representation (SR) modeling of behavior. (*A*) AIC scores for three models tested in behavioral data: 1) a baseline SR model with a free α parameter and a fixed γ to 0 (1-step; blue), 2) a SR model with free γ and α parameters (SR; green), and 3) a hybrid model with free γ and α parameters and an additional regressor for one-step probabilities (SR + 1-step; blue). Smaller AIC values indicate better model fit. (*B*) Estimates for α and γ parameters (interval: [0.01,1.0]) in the SR + 1-step model after model fitting. (*C*) P-values (on $log_{20}$ scale) of the fixed effect of the SR-based Shannon surprise and one-step transition probability in the GLM that, in combination with the SR-model parameters α and γ, best explained participants’ response times. The dashed line indicates an alpha level of 0.05. (*D*) Model derived successor representation (SR) matrices for two participants for at the last trial of each run (details about fitted parameter and group see *Top* panel). The colors indicate the normalized expected future visitation of each of the six nodes in the graph structure. Note, that the third run included the change from one to the other graph structure and the data is therefore shown separately for the two halves of the run. Boxplots and error bars are as in Fig. 2.

We Fitted the γ and α parameters of the SR model to each participant’s reaction time data (for details, see *Materials and Methods* and *SI Appendix*). A model that combined SR and 1-step predictions had the best fit (Fig. 3*A*; AICs: SR + 1-step −111899.1, SR −111887.7, 1-step −111823.7−99668.43 for baseline model; smaller values indicate better fit). Thirty-one out of 39 participants (about 80%) had estimates of γ>0.1, and the mean of the best fitting parameters for the SR + 1-step model was γ=0.51 (SD: σ(γ)=0.36 σ(γ)=0.38; Fig. 3*B*), indicating multistep graph knowledge consistent with SR models. The average learning rate was α=0.47 (σ(α)=0.43; Fig. 3*B*). Parameter recovery tests for all models yielded excellent parameter identifiability of r=1.00 and r=0.96 for α and γ, respectively (Ps <0.001; *SI Appendix*, Fig. S9 *A* and *B*; for details, see *SI Appendix*). As a sanity check, we also verified that the Shannon surprise predictor derived from the individually fitted model parameters had a significant effect for individual participants, which was the case for the large majority of participants (34 of 39, i.e., ca. 87%; Fig. 3*C*; using an α-level of 0.05). In contrast, the one-step probability regressor in the SR + 1-step model had a significant effect on response times only in one third of participants (13 of 39, ca. 33%; Fig. 3*C*). Splitting the data by graph order (uni–bi vs. bi–uni) did not result in significant differences in parameter estimates for both α and γ (ts ≥0.43, Ps ≥0.66, ds ≥0.15; *SI Appendix*, Fig. S10*A*). Relating model parameters to behavior, we found a significant negative correlation of the difference in learning slopes between high- and low-probability transitions and α (r=−0.44, P<0.001) but not γ (r=−0.28, P=0.09), indicating faster learning of multistep transitions in participants with a higher learning rate (for details, see *SI Appendix*, Fig. S9 *C* and *D*). Sequence awareness (“yes” vs. “no”) was not related to significant differences in parameter estimates for both α and γ (ts ≥1.32, Ps ≥0.20, ds ≥0.42; *SI Appendix*, Fig. S10*B*). Fitted SR matrices for two example participants are shown in Fig. 3*D*. For illustrative purposes, we selected one participant with fitted parameters close to the mean parameters in the sample (α=0.47, γ=0.51) and a participant with a deep predictive horizon (γ=0.99). *SI Appendix*, Fig. S11 illustrates SR matrices resulting from more extreme parameter combinations which indicate, as expected, that γ reflects the predictive horizon and high values of α result in encoding of only the most recent, not average, transition statistics. Individual SR matrices of all participants can be found in *SI Appendix*, Figs. S12 and S13.

### fMRI Results.

**Fig. 4.**
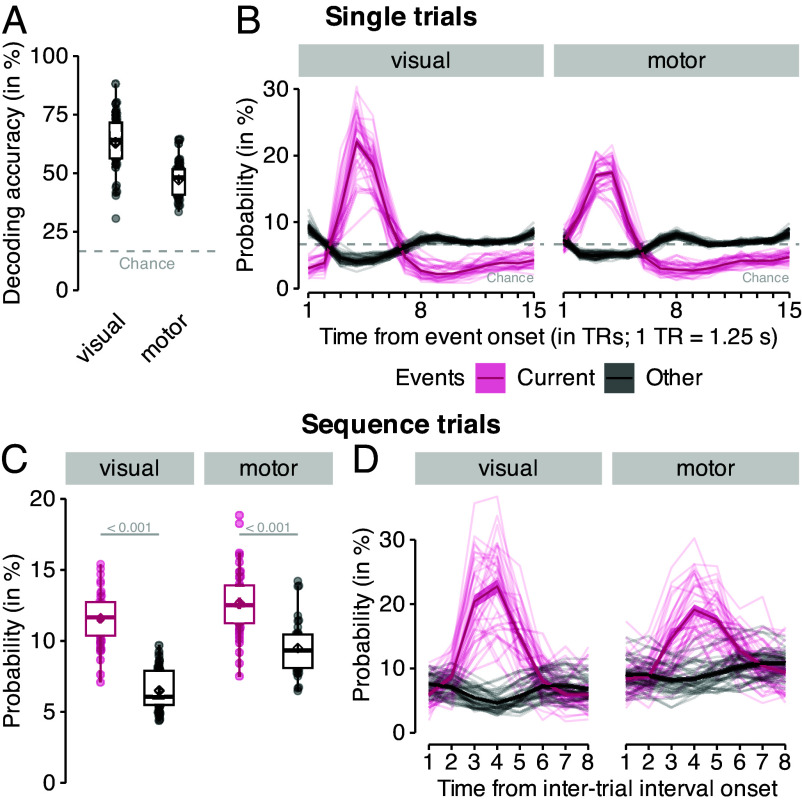
fMRI-based classification accuracy and class probability time courses. (*A*) Cross-validated leave-one-run-out classification accuracy for decoding six visual objects in occipito-temporal data (“visual”; *Left*) and six responses in sensorimotor cortex data (“motor”; *Right*) during single trials. Chance level is at 16.67% (horizontal dashed line). (*B*) Time courses (in TRs from stimulus/response onset) of class probabilities for the event on the current single trial (purple color) compared to all other events (black color), separately for both ROIs (panels). Classifier probabilities were normalized across 15 TRs, chance level at 100/15=6.67% (dashed line). (*C*) Mean classifier probability (in %; y-axis) for the event that occurred on the current sequence trial (purple color), shortly before the onset of the on-task interval, compared to all other events (black color), averaged across eight TRs in on-task intervals, separately for each ROI (panels). (*D*) Time courses (in TRs from on-task interval onset) of mean classifier probability in sequence trials for the event that occurred on the current trial (purple color) and all other events (back color). In contrast to data from single trials shown in (*B*), nonnormalized classifier probabilities are shown. Boxplots indicate the median and IQR, and diamonds, dots, and error bars are as in Fig. 2. 1 TR = 1.25 s.

To ask whether learning of map-like graph representations was accompanied by online neural replay, we first trained fMRI pattern classifiers that could detect stimulus-related activity. Logistic regression classifiers were trained on fMRI signals related to stimulus and response onsets in correct single trials, where category order was random and trials were sufficiently separated by stimulus-response intervals (SRIs) and intertrial intervals (ITIs) of 2,500 ms each (one repetition time (TR) per trial; onsets shifted by 4 s after stimulus or motor onset; one-versus-rest training; for details, see *Materials and Methods* and *SI Appendix*; cf. 59). Separate classifiers were trained on data from gray-matter-restricted anatomical (ROIs) of occipito-temporal cortex and pre- and postcentral gyri, which reflect visual object processing (cf. 60) and sensorimotor activity (e.g., ref. 61), respectively. The trained classifiers successfully distinguished between the six stimuli on single trials. Leave-one-run-out classification accuracy was M=63.08% in occipito-temporal data (SD =12.57, $t_{38}=23.06$, CI [59.69, +∞], P<0.001, compared to chance level of 16.67%, d=3.69) and M=47.05% in motor cortex data (SD =7.79%, $t_{38}=24.36$, CI [44.95, +∞], P<0.001 vs. chance, d=3.90, all P-values Bonferroni-corrected, Fig. 4*A*). Training only on data from Session 1 (eight runs of single trials) and testing on data from Session 2 (one run of single trials) indicated no decoding decrements compared to within-session testing, $F_{8,655}=0.95$, P=0.48 (*SI Appendix*, Fig. S14; for details, see *Materials and Methods* and *SI Appendix*). A time-resolved analysis of classifier probabilities over fifteen time points (TRs of 1.25 s) following event onsets showed that the normalized probability of the true stimulus class given the data peaked at the fourth TR (3.75 to 5.0 s; Fig. 4*B*) as expected based on our previous work (59). During this peak TR, the probability of the true class (i.e., the class of the current trial) was significantly higher than the mean probability of all other classes (difference between current vs. other animals in visual ROI: M=17.88, $t_{38}=21.72$, CI [16.22, 19.55], P<0.001, d=3.48; motor ROI: M=12.24, $t_{38}=32.10$, CI [11.47, 13.01], P<0.001, d=5.14, all P-values Bonferroni-corrected; Fig. 4*B*). Decoding of image categories on the localizer data in an anatomical ROI of the hippocampus did not surpass the chance level (decoding accuracy: M=16.55%, SD =1.71%; $t_{38}=0.42$, 95% CI [16.08, +∞], P=0.66, compared to chance (16.67%), d=0.07; using the same decoding approach; for details, see *SI Appendix*). We next applied the trained classifiers to data from the sequence task, where on some trials participants experienced an extended poststimulus interval of 10 s (roughly 8 TRs) during which only a fixation cross was displayed (120 trials per participant in total; 24 trials per class). As expected, the classifier probability of the animal displayed at the beginning of the extended poststimulus interval (or the corresponding motor response, respectively) was higher compared to all other classes (Fig. 4*C*), and rising and falling slowly as observed in single trials (Fig. 4*D*; mean probability of current event vs. all others in both ROIs; ts ≥11.92, Ps <0.001, ds ≥1.91, P-values Bonferroni-corrected).

Because our main focus was on replay rather than stimulus-driven activity, we removed classifier probabilities of the current stimulus in interval trials from all following analyses and asked whether we could detect sequential replay of the five stimulus categories that had not been displayed on the current trial. We tracked fast sequential replay by investigating the time-courses of the classifier evidence (7, 59). Briefly, the analysis involves applying a classifier for each stimulus class to each TR in the interval trials. Then, using simple linear regression, we tested whether the ordering of classification probabilities within each single TRs followed the graph structure (*Materials and Methods*). Our previous work has shown experimentally that this approach can detect fast neural event sequences when brief events are separated by as little as 32 ms and found analytically that the frequency spectrum of the regression coefficients is linked to sequence speed. Using the above-described analysis, the specific question we asked was whether replay would involve only one-step or multistep transitions derived from each participant’s best fitting SR model, as described above. Qualitatively, a one-step perspective predicts that in unidirectional sequence trials the classifier of either stimulus B or F should be activated following the presentation of A, given that both were one step away from image A in the forward and backward directions (Fig. 1*C*). The additional expectation derived from the SR-model was that classifier probabilities for C, D, and E would reflect their multistep SR-probabilities (C > D > E), although they all had the same one-step probability. Following our previous work (59), we also assumed that the ordering during the earlier phase of the on-task interval (TRs 1 to 4) would reflect the true directionality of the replayed sequence and would be reversed in the later phase of the interval (TRs 5 to 8), reflecting the rising and falling slopes of the underlying hemodynamic response function HRF. For example, forward replay would be indicated by forward sequentiality in earlier TRs and backward sequentiality in later TRs, while the reverse would be true for backward replay, i.e., backward sequentiality in earlier TRs and forward sequentiality in later TRs.

**Fig. 5.**
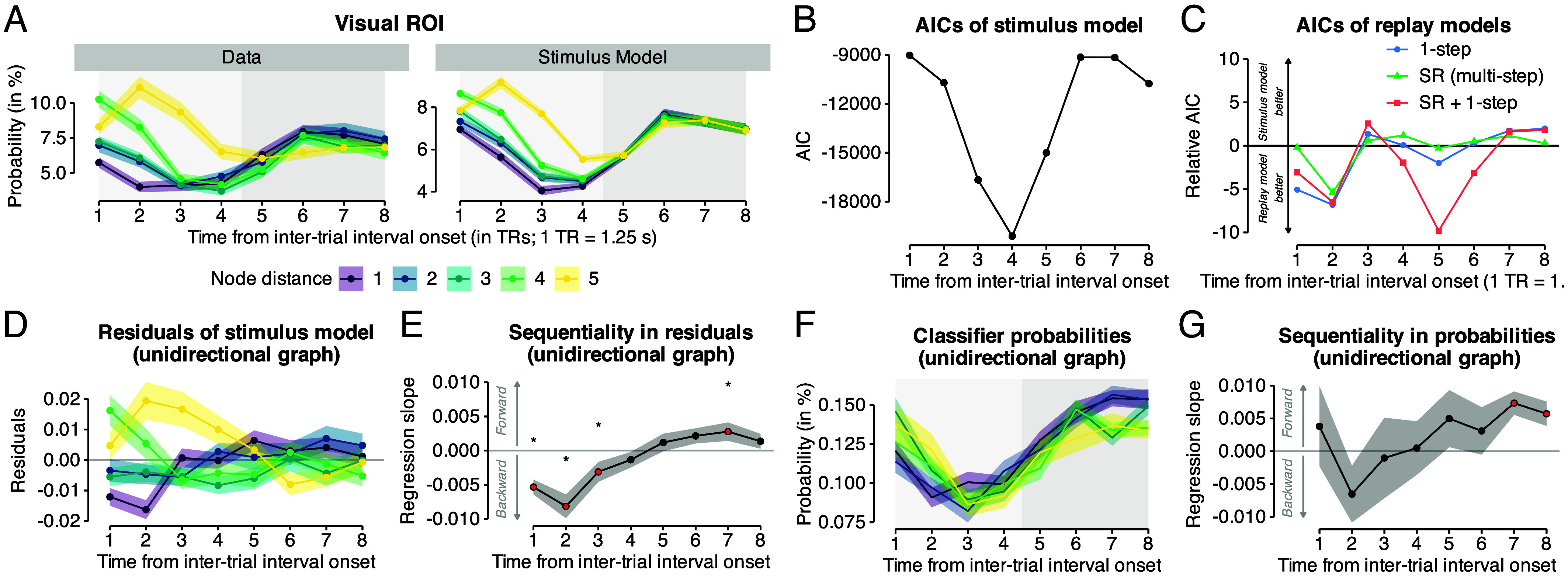
Classifier probabilities suggest replay during on-task intervals. (*A*) *Left*: Empirical classifier probability time courses during interval trials decoded from an occipito-temporal (“visual”) ROIs while participants learned the unidirectional graph structure (“Data”; *Left* panel). *Right*: simulated stimulus-driven classifier responses (“Stimulus Model”; *Right* panel; for details, see Text and *Materials and Methods*). Time courses are shown separately for the five node distances in unidirectional graph data (colors see Legend). (*B*) Time course of AIC scores of the baseline model that captured only stimulus-evoked activity. (*C*) Time courses of AIC scores of replay models, relative to baseline model shown in b (zero line = AIC stimulus model). Shown are AICs of 1) a 1-step transition model (blue), 2) a multistep SR model (green), and 3) a hybrid model with 1-step and multistep SR probabilities (red). Negative values indicate better fit of the respective LME model compared to the baseline model. (*D*) Time courses of the residuals of the stimulus model separately for the five node distances in unidirectional graph data [colors; see legend in panel (*A*)]. (*E*) Time courses of mean regression slopes relating node distance (i.e., sequential position from current node in the graph structure) to their residuals in the stimulus model as in (*D*). Positive and negative values indicate forward and backward sequentiality, respectively. Red dots and asterisks indicate significant differences from baseline (horizontal gray line at zero; all *P*s ≤0.05, uncorrected; two-sided one-sample *t* tests, one test per TR). (*F*) Time courses of mean class probabilities, after removing TRs expected to contain stimulus-evoked activation based on our modeling approach. (*G*) Time courses of mean regression slopes relating node distance to normalized classifier probability (*F*). Positive and negative values indicate forward and backward sequentiality, respectively. Red dots indicate significant differences from baseline (horizontal gray line at zero; all *P*s ≤0.05, FDR-corrected; two-sided one-sample *t* tests, one test per TR). Shaded areas represent ±1 SEM. 1 TR = 1.25 s.

A major obstacle for replay analyses during brief pauses from ongoing behavior is that the sequential ordering of previously displayed stimuli will lead to residual stimulus-evoked activation that can bias any analysis of sequential reactivation. To account for this, we modeled the stimulus-driven classifier probabilities based on the specific previous trial history of each interval, and asked whether the observed classifier probabilities reflected ordering above and beyond the trial history effects, leveraging, in particular, variability due to probabilistic transitions. Stimulus-driven classifier effects were modeled based on sine-based response functions that were fit to the time courses in (independent) single trials, as done previously in ref. 59 (with parameters for amplitude, response duration, onset delay, and baseline; see *Materials and Methods* and *SI Appendix*, Fig. S15 for example fits and comparisons across stimulus categories). The fitted participant- and stimulus-specific response functions were then convolved with the onsets of ten stimuli preceding and following each interval trial (Fig. 5*A* and *SI Appendix*, Fig. S16). We used the resulting model time courses of classifier probabilities to set up a baseline LME model to capture stimulus-driven effects during interval trials.

Using this approach, we first investigated replay in the visual ROI. As expected, the nuisance regressors reflected the overall activation patterns well (Fig. 5*A*, *Left* panel), resulting in a good overall fit of the baseline model, peaking at the fourth TR after the last stimulus was shown (P<0.001, Fig. 5*B*). To investigate replay, we compared this baseline model to a suite of three models that included additional regressors for either one-step task transition probabilities (1-step model), the individually derived SR probabilities (SR model), as well as the combination of both of these factors (SR + 1-step model; for details, see *Materials and Methods* and *SI Appendix*). For each of the three models, the corresponding regressors were entered into one LME model, separately for each ROI and TR. Fig. 5*C* shows the results for the visual cortex ROI, plotting the relative AIC scores of all models across the eight TRs of interval trials (AIC_baseline_ - AIC_model_; negative values indicate better model fit compared to the baseline model). AIC scores indicated two phases of replay during the interval. In an early phase, beginning with the second TR after interval onset, we observed strongest evidence for reactivation of only the category that is one step away from the last shown stimulus (Fig. 5*C*, AICs at TR 2 for stimulus model −10709.42, 1-step model −10716.23, SR (multistep) −10714.81, SR + 1-step −10715.90). In a later phase, between TR 4 to 6, and most strongly for TR 5, the model that jointly considered multistep SR and one-step probabilities provided the best fit of classifier probabilities (Fig. 5*C*, AICs at TR 5 for stimulus model −15005.26, 1-step model −15007.24, SR (multistep) −15005.54, SR + 1-step −15015.08). Hence, our results indicate on-task sequential reactivation of categories that were close to the last shown stimulus in participants’ mental maps of the task. The two disjoint peaks of evidence for sequential reactivation in early and late TRs align with the anticipated wave-like dynamics of sequentiality in fMRI (59), wherein forward and backward effects transiently cancel each other out, yielding an interval of reduced statistical detectability that separates the early and late phase of the interval.

To get a better understanding of the nature of this sequential reactivation, we extracted the residual classifier probabilities from the baseline model, i.e., the pattern of classifier probabilities that were above or below stimulus-driven activation resulting from the preinterval trial history. Confirming our model fitting results, these residuals indicated evidence for backward replay in the unidirectional graph condition (Fig. 5*D*). Classifier probabilities in earlier TRs were ordered in reverse relative to the tested sequence (Order after A: F>E>D>C>B) and in later TRs switched the order in the opposite direction. Testing this ordering with the regression approach developed in ref. 59 showed a significant regression slope of sequential ordering at TRs 1 to 3 and 7 (Fig. 5*E*, ts ≥2.06, Ps <0.05, ds ≥0.33, P-values uncorrected). Our previous work demonstrated that the time course of the regression slope indicates the direction and speed of neural replay (59). In light of these findings, the observed change from a negative slope in the early TRs 1 to 3 to a reversed pattern by TR 7 is consistent with backward replay occurring at a speed of 128 ms per item or slower.

We confirmed the above results by running an additional analysis that did not rely on modeling stimulus-driven influences, but rather used subsets of “clean” trials to assess the magnitude of reactivation in the absence of stimulus-driven influences. Leveraging the probabilistic nature of our task, we built a subset of the data in which all classifier probabilities were removed that reflected categories with stimulus-driven activity in the interval trial under consideration (minimum time since a category was last shown on the screen: 10 trials; time window subselection was done separately for each time point, participant and stimulus category based to our modeling approach; see *Materials and Methods*). This procedure reduced the number of trials available for further analysis to about 30% (19 out of 60 trials per participant) in the first TR and 80% (49 out of 60 trials) in the last TR of the on-task interval in the unidirectional graph condition (similar for the bidirectional graph condition; *SI Appendix*, Fig. S17*A*). Applying the same regression approach used above, we again found a similar time course of sequentiality, with a significant regression slope of ordering at TRs 7 to 8 (Fig. 5 *F* and *G*, ts ≥3.08, Ps <0.03, ds ≥0.49, P-values FDR-corrected). As before, the ordering was indicative of fast backward replay as reflected in higher classifier probabilities for categories which lay in the past in the early TRs (TRs 1 to 3) followed by the reverse in the later interval TRs (TR 7). Repeating the same analyses in a motor cortex ROI revealed no comparable evidence for on-task replay in this region (*SI Appendix*, Fig. S20).

**Fig. 6.**
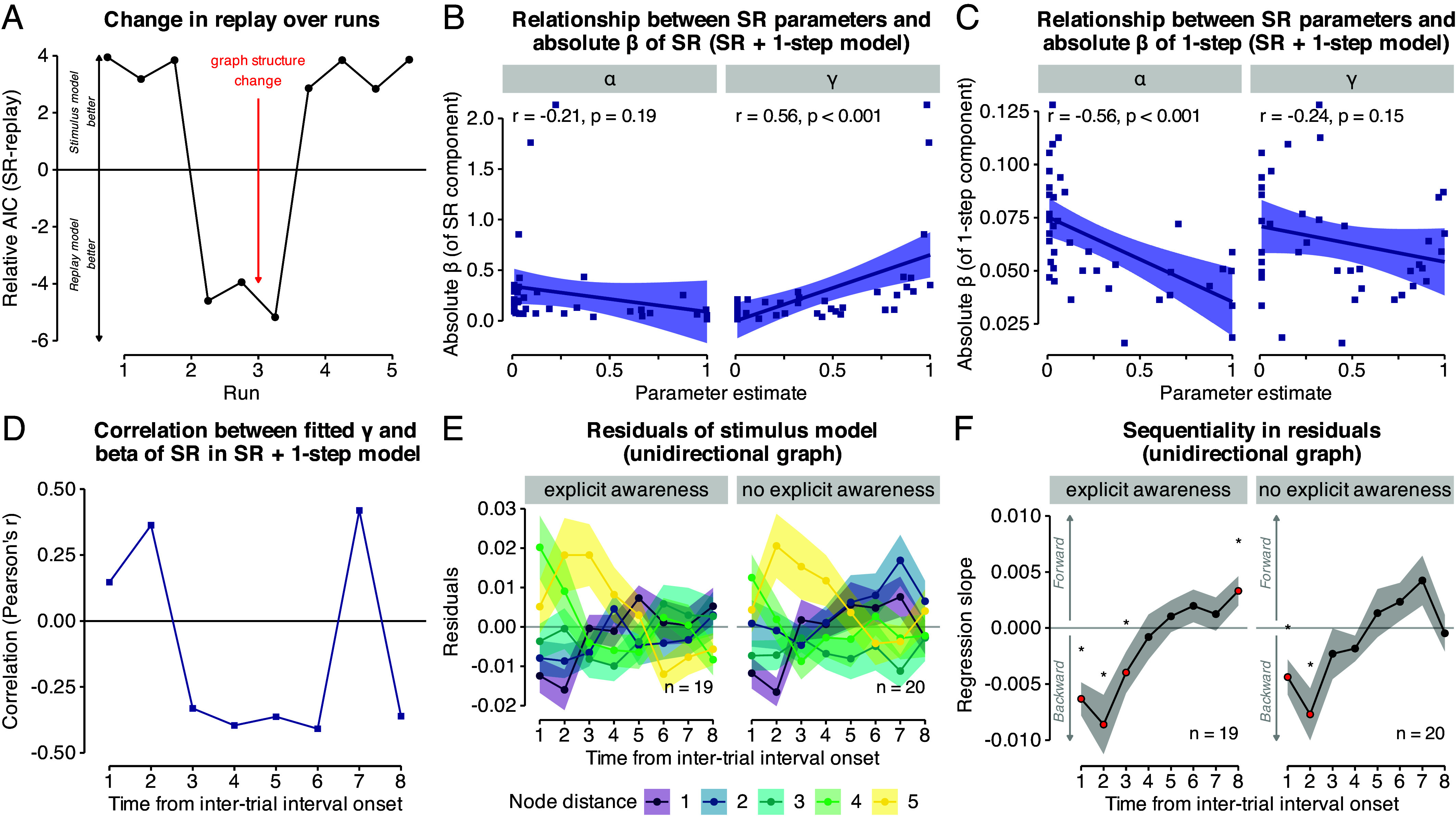
Time course of SR replay, relationship between SR replay and SR model parameters, and influence of explicit awareness. (*A*) Time courses (runs; x-axis) of model AIC scores (y-axis) relative to baseline model. The red arrow indicates the change in graph structure halfway through the third task run. Positive and negative values indicate worse and better fit, respectively, of the SR replay model compared to the stimulus model. (*B*) Relationship between the participant-wise behavioral model fit of the SR model (α and γ parameters; x-axis and panels) and the participant-wise absolute betas of the SR regressor of the SR + 1-step model averaged across all eight TRs (y-axis). (*C*) As in (*B*) but for the absolute betas of the 1-step regressor. (*D*) Time courses (in TRs from intertrial interval (ITI) onset; x-axis) of correlation coefficients (Pearson’s r; y-axis) quantifying the relationship between participant-wise betas of the regressors of the SR + 1-step model at each TR and the participant-wise behavioral model fit of the SR model (γ parameter) in data from the unidirectional task condition and visual cortex ROI. (*E*) Time courses (in TRs from ITI onset; x-axis) of the residuals of each classifier node distance of the stimulus model (colors), split by explicit awareness (plot as in Fig. 5*D*, unidirectional graph data). (*F*) Time courses (in TRs from ITI onset; x-axis) of mean regression slopes (y-axis) relating node distance to their residuals in the stimulus model as in (*E*), split by explicit awareness (“explicit awareness” vs. “no explicit awareness knowledge”; also see Fig. 2*F*). Positive and negative values indicate forward and backward sequentiality, respectively. Red dots and asterisks indicate significant differences from baseline (horizontal gray line at zero; all *P*s ≤0.05, uncorrected; two-sided one-sample *t* tests, one test per TR). 1 TR = 1.25 s.

Having established the existence of on-task replay in visual, but not motor, cortex, we asked how replay changed across learning (recall that every participant experienced both graph structures and the graph structure changed without prior announcement halfway through the task, *SI Appendix*, Fig. S4*B*). Previous theoretical accounts have suggested that replay might be particularly beneficial after the transition structure of the environment changes in order to update previous task representations (10). In order to investigate whether replay would be influenced by the change in graph structure, we split each block data into half and ran the same modeling approach as described above on the partitioned datasets and compared the AIC scores of the SR + 1-step model to the stimulus model. This analysis revealed a better fit of the SR + 1-step model compared to the stimulus model in the second half of the second run up to the second half of the third run (Fig. 6*A*). While this approach can shed some light on the time course of replay, it should be noted that what this analysis tests is specifically replay that is consistent with the tested SR model. Hence, absence of evidence could either reflect no or less replay, or replay that is less consistent with the fitted SR matrices.

Next, we asked which relation neural on-task replay had to behavior, based on the idea that SRs can be updated through replay, rather than through online experience alone (34, 35). To this end, we correlated mean absolute beta values (across the entire interval trial) of the SR regressor from the SR + 1-step model in the visual cortex with the participant-wise γ parameter of the behavioral SR model. This indicated a correlation of r=0.56 for the unidirectional graph structure (P<0.001, Fig. 6*B*). We did not find a correlation between the γ parameter and the 1-step regressor (P=0.15, uncorrected). In contrast, we found a significant negative correlation of the 1-step regressor with the participant-wise α parameter (r=−0.56, P<0.001), but not the γ parameter (r=−0.24, P=0.15). A time-resolved analysis of the relationship between on-task SR replay and the behavioral SR parameter that correlated the individual fMRI SR regressor with the individually fitted behavioral SR γ parameter showed significant negative associations in TRs 3 to 6 and 8 (rs ≤−0.33, Ps ≤0.04, FDR-corrected) and a significant positive correlation in TRs 2 and 7 (rs ≥0.36, Ps ≤0.03, FDR-corrected) in unidirectional data in the visual cortex ROI (Fig. 6*D*).

Finally, we examined whether the extent to which sequence knowledge was implicit had an effect on on-task SR replay. We first split the data of the stimulus model residuals shown in Fig. 5*D* by a) whether participants had reported being aware of the sequential task structure or not (n=19 vs. n=20; Fig. 2*F*), b) whether participants’ posttask transition probability ratings were related to the task structure or random (n=13 vs. n=26), and c) also only considered participants who met both criteria (n=24 vs. n=15; for details, see *SI Appendix*). None of these analyses revealed a qualitative differences in the time course of SR replay between groups, and indicated the same backward sequentiality dynamics in all cases (*SI Appendix*, Fig. S21). Specifically, a statistical analysis of each participants’ average slope of the regression coefficients across all TRs did not suggest any effects when comparing participants who reported vs. not to have noticed a sequence ($t_{35.46}=0.86$, P=0.40), when comparing participants who reported nonrandom transitions vs. those that did not ($t_{19.81}=0.26$, P=0.80), or when applying both criteria ($t_{34.19}=0.67$, P=0.51). This suggests that on-task backward SR replay was unrelated to explicit sequence awareness.

## Discussion

Human participants performed an incidental statistical learning task. While the task led to reportable knowledge in only a subset of participants, we found clear behavioral evidence that the majority of participants learned multistep sequential expectations as predicted by a SR model. Our neuroimaging results show online backward replay in visual cortical areas while participants paused briefly for 10 s between trials of the ongoing task. Sequential replay was consistent with an SR model that updated knowledge incrementally after each transition, and replay evidence correlated with behavioral evidence of SR model use. No evidence for a link between SR-based replay and sequence awareness was found.

Our fMRI sequentiality results are in line with our previous work (7, 59), further validate our analytic approach, but also substantially extend our knowledge about replay in the human brain. The observed time course of the sequentiality slope suggested that on-task replay was backward and, based on a comparison with data from ref. 59, occurred on a time scale on the order of 32 to 128 ms between item activations. The direction and speed of replay are notable given that they run counter to ideas that stipulate backward replay is related to reward processing, while planning would engage forward replay (62), and prior work finding forward sequential activation related to SR models (63). As we discuss below, we stipulated that replay reported in this study reflects a learning mechanism, which particularly serves to update SRs and works backward (30, 35).

In this light, we highlight three aspects of the replay observed here that differentiate our study from previous work: First, replay occurred during very short pauses from ongoing behavior, that differ substantially from the minute- or hour-long rest and sleep periods during which most replay has been detected and investigated in humans so far. Participants were also not instructed to learn about any sequentiality in the task and were not informed about the purpose of on task pauses, which furthermore occurred at unpredictable times. Second, we provide a test of whether replay is related to sequence awareness in humans and find no evidence for this idea using two different knowledge reports. Third, replay occurred only in the visual cortex, but not in the motor cortex, even though the task involved motor sequences that followed the same structure as the visual sequences.

Our behavioral results are consistent with previous findings showing that humans learn about networks of stimuli beyond one-step transitions (e.g., refs. 11, 18, 19, 24–27, and 64) and studies showing that brief pauses from a task are linked to learning and possibly involve rapid replay (50–52). Computational modeling showed that an SR model with a medium predictive horizon best explained behavioral data, thereby establishing a link between previously known behavioral effects and online TD learning of an SR model (30). Our findings add to a growing set of studies that uses models based on SRs to demonstrate the formation of predictive representations of task structure in human behavioral and neuroimaging data (12, 34, 35, 58, 65, 66). We note that statistical regularities in our main task were governed by two graph structures: one for transitions in the first half of the experiment and another for the second half. Our results therefore speak to the idea that SR learning can be considered a continuous process that adapts to environmental changes. Model fitting results and behavior that are split by graph condition and graph order are reported in *SI Appendix*, Figs. S8 and S10. Given that each participant experienced both conditions and group sizes are unequal between conditions, we argue for caution in interpreting any differences.

One unique aspect of our study was that we assessed whether knowledge reflected in replay was implicit or explicit. Our findings suggest that neural replay is most prominent during transitions in structure, the same time window during which sequence awareness seems to emerge according to self-reports. Yet, we do not find evidence for a direct link between replay and awareness. We note that this may be due to the binary awareness measure being less sensitive and more biased than the forced-choice transition reporting metric. One notable recent study has found that unconscious reactivation might lead to broader effects on memory due to a more liberal spread of activation, as compared to unconscious activation (67). Hence, our data might suggest that functionally relevant replay had no impact on awareness since our task learning crucially involved forming and updating long-range associations, rather than remembering individual elements.

Our evidence for on-task replay relates to research in rodents, where time-compressed sequential place cell activations, called theta sequences, occur during active behavior (68) and reflect multiple potential future trajectories when the animal pauses at a decision point (46), or cycle between future trajectories during movement (69) possibly reflecting an online planning process. However, in contrast to previous studies in rodents and humans (6, 49, 70), participants in our experiment likely did not engage in any (explicit) planning process, as discussed above.

One important aspect of our work is that we focused on cortical replay of predictive representations in visual (occipito-temporal) and sensorimotor (pre- and postcentral gyri) cortex. Previous work has largely focused on the hippocampus as a site of replay and as a potential brain region to host predictive cognitive maps (12, 71), while other studies have also emphasized the role of the prefrontal cortex (PFC) (72–74). Several fMRI studies demonstrated that hippocampal activity is modulated by stimulus predictability in sequential learning tasks (75–77) and is related to the reinstatement of cortical task representations in the visual cortex (40, 41, 78). Given that we were not able to decode stimulus representations in the hippocampus, it remains unclear if cortical replay occurred independently from hippocampal replay. One potential reason why we found no hippocampus involvement could be that the single trials that were used to train the classifiers were not suitable to activate the hippocampus, possibly because they did not involve any strong explicit mnemonic task component. In our previous work, we also could not successfully decode from the hippocampus but found evidence for cortical replay during rest in occipito-temporal brain regions (59). Replay is known to occur throughout the brain (see e.g., ref. 5) but the functions of distributed replay events still remain to be further illuminated. Our findings shed light on the distribution of predictive representations and replay in the human brain, and suggest a specific involvement of sensory but not motor areas. Yet, which roles the hippocampus and motor cortex play in this process remains an open question.

One shortcoming is that we cannot determine sequence order for the bidirectional graph, where clockwise and counterclockwise sequences were equally possible. While we could broadly confirm our findings of replay separately for the uni- and bidirectional conditions, no interpretation of the observed sign of the sequentiality index was possible in the latter case. The observed backward order is in line with research reporting awake replay in both forward and backward order in rodents (36, 62, 79) as well as in humans (9). Some studies suggest that the directionality of replay may be tied to different functions, such as memory consolidation, planning, or value learning (e.g., refs. 8, 10, 62, and 80). Neural sequences associated with a prospective planning function are typically in forward order relative to the experienced sequence (46, 81–83), although other studies showed that planning backward is possible (6, 84), and reported backward sequences (during theta) in rodents (85) as well as in humans (9). A broader question is to what extent our replay detection approach can be used to study mental diseases in humans. Research using MEG has suggested links between schizophrenia and replay (86), while other work has highlighted that learning gains related to short on-task pauses, possibly linked to replay, are intact in individuals with autism spectrum disorder (87). Using fMRI to detect replay during short pauses therefore promises to enhance our knowledge of these links. In conclusion, our results suggest a role of cortical replay in human implicit learning and use of predictive maps of the environment during short on-task pauses.

## Materials and Methods

### Participants and Procedures.

Forty-four participants completed the experiment. Data from five participants were excluded due to a programming error in the behavioral task (n=39; see *SI Appendix*, *Extended Methods*). The study was approved by the German Society of Psychology (ref. SchuckNicolas2020-06-22VA). All volunteers gave written informed consent before experiments started.

### Design and Experimental Tasks.

The study consisted of two MRI sessions (*SI Appendix*, Fig. S1). In Session 1, participants completed eight blocks of single trials used for classifier training as well as pre- and posttask resting-state scans. In Session 2, participants completed another run of single trials followed by five runs of sequence trials, interleaved with resting-state scans. For each participant 6 stimuli were randomly selected from a set of 24 animal images (*SI Appendix*, Fig. S2) and assigned to one of six response buttons (*SI Appendix*, Fig. S3). Participants learned the mapping of these stimuli to response buttons during 30*training trials* with pairwise balanced order of stimuli, followed by single trials runs and sequence trials runs; see above (*SI Appendix*).

During *single trial* runs (60 trials/run; 90 trials/stimulus; 540 trials total; Fig. 1*A*), participants had to press the button associated with the shown image (500 ms) within a 800 ms response window (jittered SRI of avg. 2,500ms). Feedback about the correct button followed on incorrect trials (500 ms); no feedback was given on correct trials. Trials ended with a jittered ITI of avg. 2,500 ms.

In *sequence trials* images and required responses followed a probabilistic transition structure (see below, 5 runs, 240 trials per run, 1,200 trials in total; Fig. 1*B*). As before, participants were asked to press the correct button in response to each animal image. Feedback was not provided, and in 90% of trials the images were presented at a faster pace than in single trials (800 ms image presentation, followed by an avg. 750 ms ITI with a fixation cross on the screen). In 10% of the trials (120), a 10-s ITI separated images (*interval trials*). Participants were informed that the sequence trials would be faster-paced and without feedback but were not made aware that the images now followed a sequential structure that made images partially predictable.

The order of images was governed by either a unidirectional or bidirectional graph structure (see Main text; Fig. 1*C*; the uni graph created a predominately clockwise ordering of images, in bi graph condition transitions were more balanced). Sequence trials started with one graph structure and halfway through the third run the transition structure was switched, ensuring that each participant experienced both structures and the change between them.

A post task questionnaire asked participants about handedness, whether they noticed sequential order in the stimuli, to specifying when they first noticed it (if applicable), and to rate the transition probabilities of all 30 stimulus pairs (for details, see *SI Appendix*).

### Behavioral Analyses.

Statistical analyses used a combination of linear mixed effects (LME) models, analysis of variances (ANOVAs) and t tests, including corrections for multiple testing; for details, see *SI Appendix*. One-sample or paired t tests were used to test accuracy and RTs against chance (16.67%), or to compare high vs. low transition probability trials (Fig. 2*A*, *C*, and *D* and *SI Appendix*, Fig. S5). To test multilevel factors, such as run or linear/quadratic node distance, we used LME models with the variables of interest as fixed effects factors and a random effect structure that contained subject as a random effect (Fig. 2 *B* and *E*). To estimate the learning-related speedup, we calculated participant wise regression slopes of response times across runs separately for each condition and correlated these slopes with estimates for α and γ from the SR + 1-step model.

### SR Model Fitting.

We modeled SR models for each participant based on experienced transitions during training, single, and sequence trials (*SI Appendix*). Each of the six stimuli was associated with a vector reflecting the long-term visitation probability of all stimuli, forming a 6-by-6 SR matrix ($M^{t}$) that changed trial-by-trial. The SR matrix on the first trial was initialized uniformly with $\frac{1}{36}$. After each transition between stimuli $s_{t}$ and $s_{t+1}$, the matrix row corresponding to $s_{t}$ was updated using a TD learning rule (30, 35) with a learning rate α and discount factor γ following Eq. **1**. To relate SR models to participants’ response times, we calculated Shannon information (88) for each transition from stimulus i to j was given the history of transitions up to time point t: $I\left(j\right)=-{\text{log}}_{2}\left({\tilde{m}}_{i,j}^{t}\right)$, where ${\tilde{m}}_{i,j}^{t}$ is the normalized (i,j)th entry of SR matrix $M^{t}$. SR models were fitted to each participants’ data to derive the best fitting learning rate (α) and discount factor (γ). Parameter fitting aimed to minimize the negative log-likelihood of a GLM predicting RTs using an inverse gamma link function. A nested optimization approach was used, where α and γ were adjusted via a nonlinear search method, while GLM coefficients were optimized using maximum likelihood estimation (cf. 89, 90). Both α and γ were constrained within 0.1 to 1.0. Every tested model included predictors for SR-based Shannon surprise, trial, task block, and response button. The SR + 1-step model included an additional regressor for one-step transition probabilities. For the 1-step model γ was fixed to zero. The fitted α and γ values were then used to generate participant-specific SR matrices for every trial, modeling how expectations evolved over time. In a complementary approach, we accounted for possible nuisance effects of task run, graph structure (uni- vs. bidirectional), and graph order (uni–bi vs. bi–uni) and determined the model with the best-fitting γ parameter based on the AIC in a model comparison.

### fMRI Decoding.

MRI preprocessing is described in the extended Methods. L2-regularized logistic regression classifiers were trained on fMRI data from single trials for each of the six stimuli/motor responses (c.f. 59). We added 4 s to each event onset and chose the volume closest to the assumed peaks of the BOLD response. fMRI data were detrended for each run and z-scored for each classifier and run separately for each training and test set.

### Analyses of Classifier Accuracy and Probability Time Courses.

Classification accuracy was averaged across correctly answered trials in Session 1 and compared to the 16.67% chance baseline using a one-sided t test. In Session 2 we tested whether classifiers were sensitive to the presented images by calculating the mean classifier probabilities for the current vs. all other five events in a given interval trial across all eight TRs, using two two-sided paired t tests, one for each ROI. The Bonferroni-correction method (91) was used to correct the P-values for two comparisons. Cohen’s d was used as an effect size metric (92).

### Modeling Stimulus-Driven Classifier Time Courses.

We previously found that probabilistic classifier evidence from single-trial stimuli can be broadly characterized by a sine wave-like response function, truncated after one cycle, scaled by amplitude, and adjusted to baseline (59). We captured stimulus-driven activity from previous trials during on-task intervals by modeling the classifier probability response function in the same manner. Classifier response functions were fitted to the mean classifier time courses across ten TRs on single trials, separately for each stimulus class, ROI, and participant. Next, we modeled stimulus-evoked classifier probabilities extending into the on-task interval by convolving individually fitted response functions with the onsets of ten stimuli before and after the interval. This approach accounted for stimulus-driven activity leaking into the on-task interval period and any potential combination of stimulus- and replay-driven activity extending beyond it. Finally, we computed the mean stimulus-evoked activity for each TR of the on-task interval per participant. Since the sine-based response model is a continuous function, it can be evaluated at any resolution, with the mean value approximated by the area under the curve. We evaluated the function over a 10-TR window with a sampling frequency of 0.1, noting that higher frequencies would marginally improve accuracy at the cost of computation time. To account for overlapping activation of the same stimulus from preceding trials, we summed the modeled activation values separately for each stimulus at each TR. Activation values were aggregated within 1.25 s TR bins, with missing stimulus-related activity set to zero. Stimulus-driven classifier probability time courses are illustrated in *SI Appendix*, Fig. S16.

### Analysis of Classifier Time Courses in On-Task Intervals.

We conducted analyses of classifier probabilities related to putative replay events using LME models while accounting for (modeled) stimulus-evoked activity. Specifically, we compared four different models in a model comparison. A baseline model (Model 1) described the relationship between classifier probabilities (as the main response variable) and modeled stimulus-evoked activity as the fixed effect predictor. The second LME model investigated the influence of one-step task transition probabilities by including one additional fixed effect of 1-step probabilities (one-step model; Model 2). Finally, we investigated the SR-related replay by entering the probability of individual task nodes from the SR model at the given trial count ($M^{t}$). Model 3 included only the effects of the baseline model plus the SR predictions (multistep SR model), while Model 4 added an additional predictor of one-step task transition probabilities (one-step + multistep SR model; Model 4). The models were fit to all data of the sequence learning task, separately for each ROI and TR. Note, that for each of the four models, the corresponding regressors were entered into one LME model (per ROI and TR). This procedure then allowed to compare the models at each TR of the on-task intervals, quantified by their AIC scores. All models included a random intercept of subject. Subject specific slopes led to nonsingular fit of the model and hence were removed (for details on statistical analyses, see above).

### Sequenceness Analysis.

To analyze evidence for sequential replay during interval trials, we calculated the sequentiality metric quantified by the slope of a linear regression between the (residual) classifier probabilities and the sequential orderings of a 5-item sequence in each TR, similar to previous work (59), see *SI Appendix*, *Methods* and Fig. S18]. The mean slope coefficients of all participants were compared to zero at each TR using a series of two-sided one-sample t test, separately for each condition (depending on the analyses, separated by ROI, graph structure, or sequence awareness). P-values were adjusted for multiple comparisons using FDR or Bonferroni correction (91) and Cohen’s d was used as an effect size.

### Analysis of Data without Stimulus-Evoked Activity.

We conducted an additional analysis focusing on a subset of “clean” trials that were expected to be less influenced by stimulus-evoked activation patterns. To this end, we selected for each category specific classifier only TRs from pause intervals which were separated enough from the last time that stimulus category was displayed. Hence, the cat classifier was only analyzed for interval trials in which the cat has not been displayed for at least X seconds, etc., ensuring that no stimulus-driven activity could not drive the classification timecourse. As expected, this selection reduced the number of available data points, particularly for earlier TRs (*SI Appendix*, Fig. S17). The sequenceness analysis followed the same procedures as outlined above in all other regards, ensuring that results were not confounded by stimulus-driven activity.

## Supplementary Material

Appendix 01 (PDF)

## Data Availability

We publicly share code to reproduce statistical analyses (https://github.com/lnnrtwttkhn/zoo-analysis) (93), behavioral modeling (https://github.com/lnnrtwttkhn/zoo-modeling) (94), and study and task illustration (https://github.com/lnnrtwttkhn/zoo-illustration) (95). Behavioral and fMRI data is freely available at https://gin.g-node.org/lnnrtwttkhn/zoo-bids (96).
