# Supplementary material for: Replay in the human visual cortex during brief task pauses is linked to implicit learning of successor representations
Source: Proc Natl Acad Sci U S A. 2025 Aug 22;122(34):e2507516122. doi: 10.1073/pnas.2507516122 (PMC12403150; doi:10.1073/pnas.2507516122)
Supplement: Supplementary file 1 — Appendix 01 (PDF) [file pnas.2507516122.sapp.pdf]

Supporting Information for

Replay in Human Visual Cortex During Brief Task Pauses is Linked to Implicit Learning of Successor Representations

Lennart Wittkuhn, Lena M. Krippner, Christoph Koch and Nicolas W. Schuck

Corresponding Author: Nicolas W. Schuck
E-mail: nicolas.schuck@uni-hamburg.de

This PDF file includes:

Figs. S1 to S23
Table S1
SI References

Supporting Information Results

Additional analyses of post-task transition probability ratings. Using a LME model, we found that probability ratings were influenced by the true transition probabilities of both unidirectional ($F_{1.00,1239.00} = 9.02$, $p < 0.001$) and bidirectional graph structures ($F_{1.00,1239.00} = 100.31$, $p < 0.001$), with a stronger effect for the bidirectional structure. However, neither graph order nor sequence awareness significantly predicted probability ratings (all ps ≥ 0.15). These results suggest that the bias toward bidirectional representations is not due to explicit awareness of sequence order. Instead, the findings align with prior research indicating that bidirectional associations naturally emerge from unidirectional experiences (e.g., 1) and that humans may use backward predictions strategically in decision-making (e.g., 2).

Additional analyses of SR modeling. To test whether the `SR` model accounted for the emergence of multi-step knowledge in our task, we first computed a series of SR models that covered the continuum between mere one-step learning ($\gamma = 0$) and learning over a large predictive horizon ($\gamma = 0.95$, models in steps of 0.05), using the exact stimulus sequences that each participant experienced in the task. We then asked how well response times were predicted by the myopic compared to the more far-sighted SR models by comparing the AIC scores of corresponding LME models. Each LME model regressed one participant's trial-by-trial development of multi-step knowledge as predicted by the SR model against their trial-by-trial response times, wherein transitions that were less likely according to the model should be associated with longer response times (the successor matrix $\mathbf{M}$ was converted to a Shannon surprise predictor (cf. 3), and LME models included fixed effects of task run, graph and graph order, and by-participant random intercepts and slopes; for details, see SI Methods below). This analysis showed that a discount parameter of $\gamma = 0.3$ resulted in the lowest AIC score (Fig. 3a main text), and models with non-zero γ parameters yielded substantially better fits than a model which assumed only knowledge of one-step transitions ($\gamma = 0$, leftmost data point in Fig. 3a main text). Thus, participants' response times clearly indicated multi-step graph knowledge consistent with SR models. Separate analyses for the two graph structures (uni vs. bi) and graph orders (uni – bi vs. bi – uni), showed that non-zero γ parameters achieved better fits in all cases, and indicated some differential effects of the γ parameter depending on graph structure and graph order (Fig. S8).

Supporting Information Methods

Participants. 44 young and healthy adults were recruited from an internal participant database or through local advertisement and fully completed the experiment. No statistical methods were used to predetermine the sample size but it was chosen to be larger than similar previous neuroimaging studies (e.g., 4–6). Five participants were excluded from further analysis because they viewed different task stimuli in session 1 and 2 due to a programming error in the behavioral task. Thus, the final sample consisted of 39 participants (mean age = 24.28 years, $SD = 4.24$ years, age range: 18 - 33 years, 23 female, 16 male). All participants were screened for MRI eligibility during a telephone screening prior to participation and again at the beginning of each study session according to standard MRI safety guidelines (e.g., asking for metal implants, claustrophobia, etc.). None of the participants reported to have any major physical or mental health problems. All participants were required to be right-handed, to have corrected-to-normal vision, and to speak German fluently. The ethics commission of the Deutsche Gesellschaft für Psychologie (DGPS) approved the study protocol (reference number: `SchuckNicolas2020-06-22VA`). All volunteers gave written informed consent prior to the beginning of the experiments. Every participant received 70.00 Euro and a performance-based bonus of up to 5.00 Euro upon completion of the study. None of the participants reported to have any prior experience with the stimuli or the behavioral task.

Task.

Stimuli. All visual stimuli were taken from a set of colored and shaded images commissioned by (7), which are loosely based on images from the original Snodgrass and Vanderwart set (8). The images are freely available on the internet at https://sites.google.com/andrew.cmu.edu/tarrlab/stimuli under the terms of the Creative Commons Attribution-NonCommercial-ShareAlike 3.0 Unported license (for details, see https://creativecommons.org/licenses/by-nc-sa/3.0/) and have been used in similar previous studies (e.g., 9). Stimulus images courtesy of Michael J. Tarr at Carnegie Mellon University (for details, see http://www.tarrlab.org/). In total, we selected 24 images which depicted animals that could be expected in a public zoo. Specifically, the images depicted a bear, a dromedary, a deer, an eagle, an elephant, a fox, a giraffe, a goat, a gorilla, a kangaroo, a leopard, a lion, an ostrich, an owl, a peacock, a penguin, a raccoon, a rhinoceros, a seal, a skunk, a swan, a tiger, a turtle, and a zebra (in alphabetical order; for an overview of all images, see Fig. S2). For each participant, six task stimuli were randomly selected from the set of the 24 animal images and each image was randomly assigned to one of six response buttons. This randomization ensured that any potential systematic differences between the stimuli (e.g., familiarity, preference, or ability to decode) would not influence the results on a group level (for a similar reasoning, see e.g., 10). Cages were represented by a clipart illustration of a black fence which is freely available from https://commons.wikimedia.org/wiki/File:Maki-fence-15.svg, open-source and licensed under the Creative Commons CC0 1.0 Universal Public Domain Dedication, allowing further modification (for details, see https://creativecommons.org/publicdomain/zero/1.0/). When feedback was presented in the training and single trial task conditions, correct responses were indicated by a fence colored in green and incorrect responses were signaled by a fence colored in red. The color of the original image was modified accordingly. All stimuli were presented against a white background.

Hardware and software. Behavioral responses were collected using two 4-button inline fiber optic response pads (Current Designs, Philadelphia, PA, USA), one for each hand, with a linear arrangement of four buttons. Buttons were colored in blue, yellow, green, and red, from left to right, but participants were instructed that the button color was irrelevant for the task. For an illustration of the hand placement and response button mapping, see Fig. S3b. The two response pads were attached horizontally to a rectangular cushion that was placed in participants' laps such that they could place their fingers on the response buttons with arms comfortably extended while resting on the scanner bed. Participants were asked to place their index, middle, and ring finger of their left and right hand on the yellow, green, and red buttons of the left and right response pads, respectively. The fourth (blue) button on each response pad was masked with tape and participants were instructed to never use this response button. Behavioral responses on the response pads were transferred to the computer running the experimental task and mapped to the keyboard keys `z`, `g`, `r` and `w`, `n`, `d` for the left and right hand, respectively. The task was programmed in PsychoPy3 (version 3.0.11; 11–13) and run on a Windows 7 computer with a monitor refresh-rate of 16.7 ms. We recorded the presentation time stamps of all task events (onsets of all presentations of the fixation, stimulus, SRI, response, feedback, and ITI events) and confirmed that all components of the experimental task procedure were presented as expected.

Instructions. After participants entered the MRI scanner during the first study session and completed an anatomical T1w scan and a 5 min fMRI resting-state scan, they read the task instructions while lying inside the MRI scanner (for an illustration of the study procedure, see Fig. S1). Participants were asked to read all task instructions carefully They were further instructed to clarify any potential questions with the study instructor right away and to lie as still and relaxed as possible for the entire duration of the MRI scanning procedure. As part of the instructions, participants were presented with a cover story in order to increase motivation and engagement. Participants were told to see themselves in the role of a zookeeper in training whose main task is to ensure that all animals are in the correct cages. In all task conditions, participants were asked to always keep their fingers on the response buttons to be able to respond as quickly and as accurately as possible.

Training trials. After participants read the instructions and clarified all remaining questions with the study instructors via the MRI intercom, they completed the *training* phase of the task (for an illustration of the trial procedure, see Fig. S3a). The training condition was designed to explicitly teach participants the assignment of stimuli to response buttons. Each of the six animal stimuli selected per participant was randomly assigned to one of six response buttons. For the training condition, participants were told to see themselves in the role of a zookeeper in training in a public zoo whose task is to learn which animal belongs in which cage. During each trial, participants saw six black cages at the bottom of the screen with each cage belonging to one of the six animals. On each trial, an animal appeared above one of the six cages. Participants were tasked to press the response button for that cage as fast and accurately as possible and actively remember the cage where the animal belonged. The task instructions emphasized that it would be very important for participants to actively remember which animal belonged in which cage and that they would have the chance to earn a higher bonus if they learned the assignment and responded accurately.

In total, participants completed 30 trials of the training condition. Across all trials, the pairwise ordering of stimuli was set to be balanced, with each pairwise sequential combination of stimuli presented exactly once, i.e., with $n = 6$ stimuli, this resulted in $n * (n - 1) = 6 * (6 - 1) = 30$ trials. In this sense, the stimulus order was drawn from a random walk along the graph with all nodes connected to each other and an equal probability of $p_{ij} = 0.2$ of transitioning from one node to any other node in the graph. This pairwise balancing of sequential combinations was used to ensure that participants would not learn any particular sequential order among the stimuli. Note, that this procedure only controlled for sequential order between pairs of consecutive stimuli but not higher-order sequential ordering of two steps or more.

On the first trial of the training condition, participants first saw a small black fixation cross that was displayed centrally on the screen for a fixed duration of 300 ms and signaled the onset of the following stimulus. The fixation cross was only shown on the first trial of the training phase, to allow for a short preparation signal before stimulus presentation began. Following the fixation cross, one of the animals was presented in the upper half of the screen above one of six cages that referred to the six response buttons and were presented in the lower half of the screen. The stimuli were shown for a fixed duration of 800 ms which was also the maximum time allowed for participants to respond. Note, that the instructions told participants that they would have 1 s to respond, an actual difference of 200 ms that was likely hardly noticeable by participants. Following the stimulus, participants always received feedback that was shown for a fixed duration of 500 ms. If participants responded correctly, the cage corresponding to the correctly pressed response button, was shown in green. If participants did not respond correctly, the cage referring to the correct response button was shown in green and the cage referring to the incorrectly pressed response button was shown in red. If participants responded too late, the cage referring to the correct response button was shown in green and the German words "Zu langsam" (in English: "Too slow") appeared in large red letters in the upper half of the screen. Finally, a small black fixation cross was shown during an ITI with a variable duration of $M = 1500$ ms. The ITIs were drawn from a truncated exponential distribution with a mean of $M = 1.5$ s, a lower bound of $x_1 = 1.0$ s and an upper bound of $x_2 = 10.0$ s. To this end, we used the `truncexpon` distribution from the `SciPy` package (14) implemented in Python 3 (15). The `truncexpon` distribution is described by three parameters, the shape b, the location μ and the scale β. The support of the distribution is defined by the lower and upper bounds, $[x_1, x_2]$, where $x_1 = \mu$ and $x_2 = b * \beta + \mu$. We solved the latter equation for the shape b to get $b = (x_2 - x_1)/\beta$. We chose the scale parameter β such that the mean of the distribution would be $M = 2.5$. To this end, we applied `scipy.optimize.fsolve` (14) to a function of the scale β that becomes zero when $truncexpon.mean((x_2 - x_1)/\beta, \mu, \beta) - M) = 2.5$. In total, the training phase took approximately 2 min to complete.

Single trials. After participants finished the training phase of the task in the first experimental session, they completed eight runs of the *single trials* condition and another ninth run at the beginning of the second session (for an illustration of the trial and study procedure, see main text Fig. 1a and Fig. S1). The single trial condition mainly served two purposes: First, on a behavioral level, the single trial condition was used to further train participants on the associations between animal stimuli and response keys. Second, on a neural level, the single trial condition was designed to elicit object-specific neural activation patterns of the presented visual animal stimuli and the following motor response. The resulting neural activation patterns were later used to train multivariate classifiers (for details, see below). The cover story of the instructions told participants that they would be tested on how well they have learned the association between animals and response keys during the training phase.

In total, participants completed nine runs of the single trial condition. Eight runs were completed during session 1 and an additional ninth run was completed at the beginning of session 2 in order to remind participants about the S-R mappings (for an illustration of the study procedure, see Fig. S1). Each run consisted of 60 trials. As in the training phase, the proportion of pairwise sequential combinations of stimuli was balanced within a run. Across all trials, each pairwise sequential combination of stimuli was presented twice, i.e., with $n = 6$ stimuli, this results in $n * (n - 1) * 2 = 6 * (6 - 1) * 2 = 60$ trials. As for the training trials, the sequential ordering of stimuli was drawn from a graph with all nodes connected to each other and an equal probability of $p_{ij} = 0.2$ of transitioning from one node to any other node in the graph. With 60 trials per run, each of the six animal stimuli was shown 10 times per run. Given nine runs of the single trial condition in total, this amounted to a maximum of 90 trials per stimulus per participant of training examples for the classifiers. Including a ninth run at the beginning of session 2 offered two advantages. First, participants were reminded about the associations between the stimuli and response keys that they had learned extensively during session 1. Second, the ninth run allowed to investigate decoding performance across session boundaries. Note, that the two experimental sessions were separated by about one week. Although the pre-processing of fMRI data (for details, see section on fMRI data pre-processing below) should align the data of the two sessions, remaining differences between the two sessions (e.g., positioning of the participant in the MRI scanner) could lead to a decrement in decoding accuracy when testing classifiers that were trained on session 1 data to data from session 2. Our decoding approach was designed such that pattern classifiers would be mainly trained on neural data from single trials in session 1 but then applied to data from sequence trials in session 2.

As in training trials, the first trial of each run in the single trial phase started with a black fixation cross on a white background that was presented for a fixed duration of 300 ms. Only the first trial of a run contained a fixation cross, to provide a preparatory signal for participants which would later be substituted for by the ITI. Participants were then presented with one of the six animal stimuli that was presented centrally on the screen for a fixed duration of 500 ms. Participants were instructed to not respond to the stimulus. To check if participants indeed did not respond during the stimulus or the following SRI, we also recorded responses during these trial events. During the breaks between task runs, participants received feedback about the proportion of trials on which they responded too early. If participants responded too early, they were reminded by the study instructors to not respond before the response screen. A variable SRI followed the stimulus presentation during which a fixation cross was presented again. Including a jittered SRI ensured that the neural responses to the visual stimulus and the motor response could be separated in time and reduced temporal autocorrelation. Following the SRI, the cages indicating the response buttons were displayed centrally on the screen for a fixed duration of 800 ms, which was also the response time limit for participants. If participants responded incorrectly, the cage referring to the correct response button was shown in green and the cage referring to the incorrectly pressed response key was shown in red. If participants responded too late, the cage referring to the correct response button was shown in green and the German words "Zu langsam" (in English: "Too slow") appeared in large red letters in the upper half of the screen. If participants responded correctly, the feedback screen was skipped. Each trial ended with an ITI with a variable duration of $M = 2.5$ s. Both SRIs and ITIs were drawn from a truncated exponential distribution as on training trials (for details, see the description of training trials above).

Sequence trials. Following the ninth run of the single trial condition in the second session, participants completed five runs of the *sequence trials* condition (for an illustration of the study procedure, see Fig. S1). During sequence trials, participants were exposed to a fast-paced stream of the same six animal stimuli as in the training and single trial phase. While the transition probabilities between stimuli were balanced in single trials, unbeknownst to participants, the sequential ordering of animal stimuli in sequence trials followed particular transition probabilities.

During sequence trials, the sequential order of stimuli across trials was determined by two graph structures with distinct transition probabilities. In the first graph structure, each node had a high probability ($p_{ij} = 0.7$) of transitioning to the next neighboring (i.e., transitioning from A to B, B to C, C to D, D to E, E to F, and F to A). Transitions to all other nodes (except the previous node) happened with equal probability of 0.1. Transitions to the previous node never occurred (transition probability of $p_{ij} = 0.0$). These transition probabilities resulted in a sequential ordering of stimuli that can be characterized by a continuous progression in a unidirectional (i.e., clockwise) order around the ring-like graph structure. We therefore termed this graph structure the *unidirectional graph* (or *uni*, in short). The second graph structure allowed sequential ordering that could also progress in counterclockwise order. To this end, stimuli were now equally likely to transition to the next neighboring but also the previous node (probability of $p_{ij} = 0.35$, i.e., splitting up the probability of $p_{ij} = 0.7$ of transitioning to the next neighboring node only in the unidirectional graph structure). As in the unidirectional graph, transitions to all other nodes happened with equal probability of $p_{ij} = 0.1$. Given that stimuli could follow a sequential ordering in both directions of the ring, we refer to this graph structure as the *bidirectional graph* (or *bi*, in short).

Participants completed five runs of the sequence trials. Each run consisted of 240 trials. Each stimulus was shown 40 times per run. In the unidirectional graph, for each stimulus the most likely transitions (probability of $p_{ij} = 0.7$) to the next

neighboring node occurred 28 times per participant. Per stimulus and participant, 4 transitions to the other three possible nodes (low probability of $p_{ij} = 0.1$) happened. No transitions to the previous node happened when stimulus transitions were drawn from a unidirectional graph structure. Together, this resulted in $28 + 4 * 3 = 40$ presentations per stimulus, run and participant. For the bidirectional graph structure, transitions to the next neighboring and the previous node occurred 14 times per stimulus and to all other nodes 4 times as for the unidirectional graph structure. Together, this resulted in $14 + 14 + 4 * 3 = 40$ presentations per stimulus, run and participant. The equal presentation of stimuli within a run also ruled out the possibility that replay could be influenced by the disproportionate repetition of individual stimuli, as demonstrated in the study by (16).

As for the other task conditions, only the first trial of the sequence trial phase started with the presentation of a small black fixation cross that was presented centrally on the screen for a fixed duration of 300 ms. Then, an animal stimulus was presented centrally on the screen for a fixed duration of 800 ms, which also constituted the time limit in which participants could respond with the correct response button. Participants did not receive feedback during sequence trials in order to avoid any influence of feedback on sequence learning. The stimulus was followed by an ITI with a mean duration of 750 ms. The ITI in the sequence trials was also drawn from a truncated exponential distribution with a mean of $M = 750$ ms, a lower bound of $x_1 = 500$ ms and an upper bound of $x_2 = 5000$ ms.

Importantly, during the sequence trials, we also included long ITIs of 10 s in order to investigate on-task replay. As stated above, participants completed 240 trials of the sequence trials per run. In each run, each stimulus was shown on a total of 40 trials. For each stimulus, every 10th trial on average was selected to be followed by a long ITI of 10 s. This meant that in each of the five main task runs, 4 trials per stimulus were followed by a long ITI. In total, each participant experienced 24 long ITI trials per run and 120 long ITI trials across the entire experiment. The duration of 10 s (roughly corresponding to eight TRs at a TR of 1.25 s) was chosen based on our previous results showing that the large majority of sequential fMRI signals can be captured within this time period (cf. 17, their Fig. 3).

Post-task questionnaire. After participants left the scanner in session 2, they were asked to complete a computerized post-task questionnaire consisting of four parts. First, participants were asked to report their handedness by selecting from three alternative options, "left", "right" or "both", in a forced-choice format. Note, that participants were required to be right-handed to participate in the study, hence this question merely served to record the self-reported handedness in addition to the participant information acquired as part of the recruitment procedure and demographic questionnaire assessment. Second, participants were asked whether they noticed any sequential order among the animal stimuli during sequence trials and could respond either "yes" or "no" in a forced-choice format. Third, if participants indicated that they had noticed a sequential order of the stimuli (i.e., if they answered "yes" to the previous question), they were asked to indicate during which run of the sequence trials they had started to notice the ordering (selecting from run "1" to "5"). In case participants indicated that they did not notice a sequential ordering, they were asked to select "None" when asked about the run. Fourth, participants were presented with all sequential combinations of pairs of the animal stimuli and asked to indicate how likely animal A (on the left) was followed by animal B (on the right) during the sequence trial condition of the task. Participants were instructed to "follow their gut feeling" in case they were uncertain about the probability ratings. With $n = 6$ stimuli, this resulted in $n * (n - 1) = 6 * (6 - 1) = 30$ trials. Participants indicated their response using a horizontal slider on a continuous scale from 0% to 100%. We recorded participants probability rating and response time on each trial. There was no time limit for any of the assessments in the questionnaire. Participants took $M = 5.49$ min ($SD = 2.38$ min; range: 2.23 to 12.63 min) to complete the questionnaire. The computerized questionnaire was programmed in PsychoPy3 (version 3.0.11; 11–13) and run on the same Windows 7 computer that was used for the main experimental task.

Study procedure. All participants were screened for study and MRI eligibility during a telephone screening prior to participation. The study consisted of two experimental sessions. For an illustration of the study procedure, see Fig. S1. As data collection took place during the COVID-19 pandemic, upon arrival at the study center in both sessions, participants were first asked about any symptoms that could indicate an infection with the SARS-CoV-2 virus. The study instructors then measured participants' body temperature which was required to not be higher than $37.5°C$. Participants were asked to read and sign all relevant study documents at home prior to their arrival at the study center.

Session 1. The first MRI session (Fig. S1a) started with a short localizer sequence of ca. 1 min during which participants were asked to rest calmly, close their eyes and move as little as possible. Once the localizer data was acquired, the study staff aligned the FOV for the acquisition of the T1w sequence. The acquisition of the T1w sequence took about 4 min to complete. Using the anatomical precision of the T1w images, the study staff then aligned the FOV for the functional MRI sequences. Here, the lower edge of the FOV was first aligned to the visually identified AC-PC line of the participant's brain. The FOV was then manually titled by 20 degrees forwards relative to the rostro-caudal axis (positive tilt; for details see the section on "" on page 6). Shortly before the functional MRI sequences were acquired, we performed Advanced Shimming. During the shimming period, which took ca. 2 min, participants were again instructed to move as little as possible and additionally asked to avoid swallowing to further reduce any potential movements. Next, we acquired functional MRI data during a resting-state period of 5 min. For this phase, participants were instructed to keep their eyes open and fixate a white fixation cross that was presented on a black background. Acquiring fMRI resting-state data before participants had any exposure to the task (including task instructions) allowed us to record a resting-state period that was guaranteed to be free of any task-related neural activation or reactivation. Following this pre-task resting-state scan, participants read the task instructions inside the MRI scanner and were

able to clarify any questions with the study instructions via the intercom system. Participants then performed the training phase of the task (for details, see the section “” on page 3; Fig. S3) while undergoing acquisition of functional MRI data. The training phase took ca. 2 min to complete. Following the training phase, participants performed eight runs of the single trial phase of the task of ca. 6 min each (for details, see section “” on page 4; main text Fig. 1a) while fMRI data was recorded. Before participants left the scanner, field maps were acquired.

Session 2. At the beginning of the second session (Fig. S1b), participants first completed the questionnaire for MRI eligibility and the questionnaire on COVID-19 symptoms before entering the MRI scanner again. As in the first session, the second MRI session started with the acquisition of a short localizer sequence and a T1w sequence followed by the orientation of the FOV for the functional acquisitions and Advanced Shimming. Participants were asked to rest calmly and keep their eyes closed during this period. Next, during the first functional sequence of the second study session, participants performed a ninth run of the single trial phase of the task in order to remind them about the correct response buttons associated with each of the six stimuli. We then acquired functional resting-state scans of 3 min each and functional task scans of 10 min each in an interleaved fashion, starting with a resting-state scan. During the acquisition of functional resting-state data, participants were asked to rest calmly and fixate a small white cross on a black background that was presented on the screen. During each of the functional task scans, participants performed the sequence task (for details, see section “” on page 4; main text Fig. 1b). Importantly, half-way through the third block of the sequence task, the graph structure was changed without prior announcement towards the second graph structure. After the sixth resting-state acquisition, field maps were acquired and participants eventually left the MRI scanner.

MRI data acquisition. All MRI data were acquired using a 32-channel head coil on a research-dedicated 3-Tesla Siemens Magnetom TrioTim MRI scanner (Siemens, Erlangen, Germany) located at the Max Planck Institute for Human Development in Berlin, Germany.

At the beginning of each of the two MRI recording sessions, high-resolution T1w anatomical MPRAGE sequences were obtained from each participant to allow co-registration and brain surface reconstruction (sequence specification: 256 slices; TR = 1900 ms; TE = 2.52 ms; flip angle = 9 degrees; TI = 900 ms; matrix size = 192 x 256; FOV = 192 x 256 mm; voxel size = 1 x 1 x 1 mm).

For the functional scans, whole-brain images were acquired using a segmented k-space and steady state T2*-weighted multi-band echo-planar imaging single-echo gradient sequence that is sensitive to the BOLD contrast. This measures local magnetic changes caused by changes in blood oxygenation that accompany neural activity (sequence specification: 64 slices in interleaved ascending order; A-P phase encoding direction; TR = 1250 ms; TE = 26 ms; voxel size = 2 x 2 x 2 mm; matrix = 96 x 96; FOV = 192 x 192 mm; flip angle = 71 degrees; distance factor = 0%; multi-band acceleration factor 4). Slices were tilted for each participant by 20 degrees forwards relative to the rostro-caudal axis (positive tilt) to improve the quality of fMRI signal from the hippocampus (cf. 18) while preserving sufficient coverage of occipito-temporal and motor brain regions. The same sequence parameters were used for all acquisitions of fMRI data. For each functional task run, the task began after the acquisition of the first four volumes (i.e., after 5.00 s) to avoid partial saturation effects and allow for scanner equilibrium.

The first MRI session included nine functional task runs in total. After participants read the task instructions inside the MRI scanner, they completed the training trials of the task which explicitly taught participants the correct mapping between stimuli and response keys. During this task phase, 80 volumes of fMRI were collected, which were not used in any further analysis. The other eight functional task runs during session 1 consisted of eight runs of the single trial condition. Each run of the single trial task was about 6 min in length, during which 320 functional volumes were acquired. We also recorded two functional runs of resting-state fMRI data, one before and one after the task runs. Each resting-state run was about 5 min in length, during which 233 functional volumes were acquired.

The second MRI session included six functional task runs in total. After participants entered the MRI scanner, they completed an additional ninth run of the single trial task. As before, this run of the single trial task was also about 6 min in length, during which 320 functional volumes were acquired. Participants then completed five runs of the sequence task. Each run of the five sequence task runs was about 10 min in length, during which 640 functional volumes were acquired. The five runs of the sequence task were interleaved with six recordings of resting-state fMRI data, each 3 min in length, during which 137 functional volumes were acquired.

At the end of each scanning session, two short acquisitions with six volumes each were collected using the same sequence parameters as for the functional scans but with varying phase encoding polarities, resulting in pairs of images with distortions going in opposite directions between the two acquisitions (also known as the *blip-up / blip-down* technique). From these pairs the displacement maps were estimated and used to correct for geometric distortions due to susceptibility-induced field inhomogeneities as implemented in the `fMRIPrep` preprocessing pipeline (19, for details, see below). In addition, a whole-brain spoiled GR field map with dual echo-time images (sequence specification: 36 slices; A-P phase encoding direction; TR = 400 ms; TE1 = 4.92 ms; TE2 = 7.38 ms; flip angle = 60 degrees; matrix size = 64 x 64; FOV = 192 x 192 mm; voxel size = 3 x 3 x 3.75 mm) was obtained as a potential alternative to the blip-up / blip-down method described above.

We also measured respiration during each scanning session using a pneumatic respiration belt as part of the Siemens physiological measurement unit. Pulse data could not be recorded as the recording device could not be attached to the participants' index finger as it would have otherwise interfered with the motor responses using the index finger (see Fig. S3b).

MRI data preparation.

Arrangement of data according to the Brain Imaging Data Structure (BIDS) standard. The majority of the steps involved in preparing and preprocessing the MRI data employed recently developed tools and workflows aimed at enhancing standardization and reproducibility of task-based fMRI studies (for a similar data processing pipeline, see e.g., 17, 20). Version-controlled data and code management was performed using `DataLad` (version 0.19.6; 21, 22), supported by the `DataLad` Handbook (23). Following successful acquisition, all study data were arranged according to the Brain Imaging Data Structure (BIDS) specification (24) using the `HeuDiConv` tool (version 0.8.0.2; freely available from https://github.com/ReproNim/reproin or https://hub.docker.com/r/repronim/reproin) in combination with the `ReproIn` heuristic (25, version 0.6.0) that allows for automated creation of BIDS data sets from the acquired DICOM images. To this end, the sequence protocol of the MRI data acquisition was set up to conform with the specification required by the `ReproIn` heuristic (for details of the heuristic, see https://github.com/nipy/heudiconv/blob/master/heudiconv/heuristics/reproin.py). `HeuDiConv` was run inside a `Singularity` container (26, 27) that was built from the most recent version (at the time of access) of a `Docker` container (tag 0.8.0.2), available from https://hub.docker.com/r/repronim/reproin/tags. DICOMs were converted to the NIfTI-1 format using `dcm2niix` (version 1.0.20190410GCC6.3.0; 28). In order to make personal identification of study participants unlikely, we eliminated facial features from all high-resolution structural images using `pydeface` (version 2.0.0; 29, available from https://github.com/poldracklab/pydeface or https://hub.docker.com/r/poldracklab/pydeface). `pydeface` (29) was run inside a `Singularity` container (26, 27) that was built from the most recent version (at the time of access) of a `Docker` container (tag 37-2e0c2d), available from https://hub.docker.com/r/poldracklab/pydeface/tags and used `Nipype`, version `1.3.0-rc1` (30, 31). During the process of converting the study data to BIDS the data set was queried using `pybids` (version 0.12.1; 32, 33), and validated using the `bids-validator` (version 1.5.4; 34). The `bids-validator` (34) was run inside a `Singularity` container (26, 27) that was built from the most recent version (at the time of access) of a `Docker` container (tag v1.5.4), available from https://hub.docker.com/r/bids/validator/tags.

MRI data quality control. The data quality of all functional and structural acquisitions was evaluated using the automated quality assessment tool `MRIQC`, version 0.15.2rc1 (for details, see 35, and the `MRIQC` documentation, available at https://mriqc.readthedocs.io/en/stable/). The visual group-level reports of the estimated image quality metrics confirmed that the overall MRI signal quality of both anatomical and functional scans was highly consistent across participants and runs within each participant.

MRI data preprocessing. Preprocessing of MRI data was performed using `fMRIPrep` 20.2.0 (Long Term Support (LTS) release; 19, 36, RRID:SCR_016216), which is based on `Nipype` 1.5.1 (30, 31, RRID:SCR_002502). Many internal operations of `fMRIPrep` use `Nilearn` 0.6.2 (37, RRID:SCR_001362), mostly within the functional processing workflow. For more details of the pipeline, see the section corresponding to workflows in `fMRIPrep`'s documentation at https://fmriprep.readthedocs.io/en/latest/workflows.html. Note, that version 20.2.0 of `fMRIPrep` is a Long-Term Support release, offering long-term support and maintenance for four years.

Preprocessing of anatomical MRI data using `fMRIPrep`. A total of two T1w images were found within the input BIDS data set, one from each study session. All of them were corrected for intensity non-uniformity using `N4BiasFieldCorrection` (38), distributed with ANTs 2.3.3 (39, RRID:SCR_004757). The T1w-reference was then skull-stripped with a Nipype implementation of the `antsBrainExtraction.sh` workflow (from ANTs), using `OASIS30ANTs` as target template. Brain tissue segmentation of CSF, white matter and grey matter was performed on the brain-extracted T1w using `fast` (FSL 5.0.9, RRID:SCR_002823, 40). A T1w-reference map was computed after registration of two T1w images (after intensity non-uniformity-correction) using `mri_robust_template` (FreeSurfer 6.0.1, 41). Brain surfaces were reconstructed using `recon-all` (FreeSurfer 6.0.1, RRID:SCR_001847, 42), and the brain mask estimated previously was refined with a custom variation of the method to reconcile ANTs-derived and FreeSurfer-derived segmentations of the cortical grey matter of Mindboggle (RRID:SCR_002438, 43). Volume-based spatial normalization to two standard spaces (MNI152NLin6Asym, MNI152NLin2009cAsym) was performed through nonlinear registration with `antsRegistration` (ANTs 2.3.3), using brain-extracted versions of both T1w reference and the T1w template. The following templates were selected for spatial normalization: FSL's MNI ICBM 152 non-linear 6th Generation Asymmetric Average Brain Stereotaxic Registration Model (44, RRID:SCR_002823; TemplateFlow ID: MNI152NLin6Asym), ICBM 152 Nonlinear Asymmetrical template version 2009c (45, RRID:SCR_008796; TemplateFlow ID: MNI152NLin2009cAsym).

Preprocessing of functional MRI data using `fMRIPrep`. For each of the BOLD runs found per participant (across all tasks and sessions), the following preprocessing was performed. First, a reference volume and its skull-stripped version were generated using a custom methodology of `fMRIPrep`. A B0-nonuniformity map (or fieldmap) was estimated based on two (or more) echo-planar imaging references with opposing phase-encoding directions, with `3dQwarp` (46, AFNI 20160207). Based on the estimated susceptibility distortion, a corrected echo-planar imaging reference was calculated for a more accurate co-registration with the anatomical reference. The BOLD reference was then co-registered to the T1w reference using `bbregister` (FreeSurfer) which implements boundary-based registration (47). Co-registration was configured with six degrees of freedom. Head-motion parameters with respect to the BOLD reference (transformation matrices, and six corresponding rotation and translation parameters) are estimated before any spatiotemporal filtering using `mcflirt` (FSL 5.0.9, 48). BOLD runs were slice-time corrected using `3dTshift` from AFNI 20160207 (46, RRID:SCR_005927). The BOLD time-series were resampled onto the following surfaces (FreeSurfer reconstruction nomenclature): `fsnative`. The BOLD time-series (including slice-timing correction) were resampled onto their original, native space by applying a single, composite transform to correct for head-motion and susceptibility distortions. These resampled BOLD time-series will be referred to as preprocessed BOLD in original space, or just preprocessed BOLD. The BOLD time-series were resampled into standard space, generating a preprocessed BOLD run in `MNI152NLin6Asym`

space. First, a reference volume and its skull-stripped version were generated using a custom methodology of `fMRIPrep`. Several confounding time-series were calculated based on the preprocessed BOLD: framewise displacement, DVARS and three region-wise global signals. framewise displacement was computed using two formulations following (49) (absolute sum of relative motions) and (48) (relative root mean square displacement between affines). Framewise displacement and DVARS are calculated for each functional run, both using their implementations in `Nipype` (following the definitions by 49). The three global signals are extracted within the CSF, the white matter, and the whole-brain masks. Additionally, a set of physiological regressors were extracted to allow for component-based noise correction (`CompCor`, 50). Principal components are estimated after high-pass filtering the preprocessed BOLD time-series (using a discrete cosine filter with $128s$ cut-off) for the two `CompCor` variants: temporal (`tCompCor`) and anatomical (`aCompCor`). `tCompCor` components are then calculated from the top 2% variable voxels within the brain mask. For `aCompCor`, three probabilistic masks (CSF, white matter and combined CSF+white matter) are generated in anatomical space. The implementation differs from that of (50) in that instead of eroding the masks by 2 pixels on BOLD space, the `aCompCor` masks are subtracted from a mask of pixels that likely contain a volume fraction of grey matter. This mask is obtained by dilating a grey matter mask extracted from the FreeSurfer's `aseg` segmentation, and it ensures components are not extracted from voxels containing a minimal fraction of grey matter. Finally, the masks are resampled into BOLD space and binarized by thresholding at 0.99 (as in the original implementation). Components are also calculated separately within the white matter and CSF masks. For each `CompCor` decomposition, the k components with the largest singular values are retained, such that the retained components' time series are sufficient to explain 50 percent of variance across the nuisance mask (CSF, white matter, combined, or temporal). The remaining components are dropped from consideration. The head-motion estimates calculated in the correction step were also placed within the corresponding confounds file. The confound time series derived from head motion estimates and global signals were expanded with the inclusion of temporal derivatives and quadratic terms for each (51). Frames that exceeded a threshold of 0.5 mm framewise displacement or 1.5 standardized DVARS were annotated as motion outliers. All resamplings can be performed with a single interpolation step by composing all the pertinent transformations (i.e. head-motion transform matrices, susceptibility distortion correction when available, and co-registrations to anatomical and output spaces). Gridded (volumetric) resamplings were performed using `antsApplyTransforms` (ANTs), configured with Lanczos interpolation to minimize the smoothing effects of other kernels (52). Non-gridded (surface) resamplings were performed using `mri_vol2surf` (FreeSurfer).

Additional preprocessing of functional MRI data following `fMRIPrep`. Following preprocessing using `fMRIPrep`, the fMRI data were spatially smoothed using a Gaussian mask with a standard deviation (FWHM parameter) set to 4 mm using the example `Nipype` smoothing workflow `create_susan_smooth` based on the SUSAN algorithm as implemented in FSL (53). In this workflow, each run of fMRI data was separately smoothed using FSL's SUSAN algorithm with the brightness threshold set to 75% of the median value of each run and a mask constituting the mean functional image of each run.

Multi-variate fMRI pattern analysis. All fMRI pattern classification analyses were conducted using the open-source `Python` (Python Software Foundation, Python Language Reference, version 3.8.6) packages `Nilearn` (version 0.7.0; 37) and `scikit-learn` (version 0.24.1; 54). In all classification analyses, we trained an ensemble of six independent classifiers, one for each of the six event classes. Depending on the analysis, these six classes either referred to the identity of the six visual animal stimuli or the identity of the participant's motor response, when training the classifiers with respect to the stimulus or the motor onset, respectively. For each class-specific classifier, labels of all other classes in the data were relabeled to a common "other" category. In order to ensure that the classifier estimates were not biased by relative differences in class frequency in the training set, the weights associated with each class were adjusted inversely proportional to the class frequencies in each training fold. Given that there were six classes to decode, the frequencies used to adjust the classifiers' weights were $\frac{1}{6}$ for the class of interest, and $\frac{5}{6}$ for the "other" class, comprising any other classes. Adjustments to minor imbalances caused by the exclusion of erroneous trials were performed in the same way. We used separate logistic regression classifiers with identical parameter settings. All classifiers were regularized using L2 regularization. The C parameter of the cost function was fixed at the default value of $C = 1.0$ for all participants. The classifiers employed the `lbfgs` algorithm to solve the multi-class optimization problem and were allowed to take a maximum of $4,000$ iterations to converge. Pattern classification was performed within each participant separately, never across participants. For each example in the training set, we added 4 s to the event onset and chose the volume closest to that time point (i.e., rounding to the nearest volume) to center the classifier training on the expected peaks of the BOLD response (i.e., accounting for hemodynamic lag; for a similar approach, see e.g., 55). At a TR of 1.25 s this corresponded roughly to the fourth MRI volume which thus compromised a time window of 3.75 s to 5.0 s after each event onset. We detrended the fMRI data separately for each run across all task conditions to remove low frequency signal intensity drifts in the data due to noise from the MRI scanner. For each classifier and run, the features were standardized (z-scored) by removing the mean and scaling to unit variance separately for each training and test set.

Classification procedures. First, in order to assess the ability of the classifiers to decode the correct class from fMRI patterns, we conducted a leave-one-run-out cross-validation procedure for which data from seven task runs of the single trials in session 1 were used for training and data from the left-out run (i.e., the eighth run) from session 1 was used for testing the classification performance. This procedure was repeated eight times so that each task run served as the testing set once. Classifier training was performed on data from all correct single trials of the seven runs in the respective cross-validation fold. Note that category order was randomized and trials were sufficiently separated to reduce temporal autocorrelation (SRIs and ITIs each 2500 ms, see procedure of single trials, cf. 17, 56). In each iteration of the leave-one-run-out procedure, the classifiers trained on seven

out of eight runs were then applied separately to the data from the left-out run. Specifically, the classifiers were applied to (1) data from the single trials of the left-out run, selecting volumes capturing the expected activation peaks to determine classification accuracy, and (2) data from the single trials of the left-out run, selecting all volumes from the volume closest to the stimulus or response onset and the next fifteen volumes to characterize temporal dynamics of probabilistic classifier predictions on a single trial basis.

Second, we assessed decoding performance on single trials across the two experimental sessions. The large majority of fMRI data that was used to train the classifiers was collected in session 1 (eight of nine runs of the single trials), but the trained classifiers were mainly applied to fMRI data from session 2 (i.e., on-task intervals during sequence trials). At the beginning of the second experimental session, participants completed another run of the single trials (i.e., a ninth run; for the study procedure, see Fig. S1). This additional task run mainly served the two purposes of (1) reminding participants about the correct S-R mapping that they had learned in session 1, and (2) to investigate the ability of the classifiers to correctly decode fMRI patterns in session 2 when they were only trained on session 1 data. This second aspect is crucial, as the main focus of investigation is the potential reactivation of neural task representations in session 2 fMRI data. Thus, it is important to demonstrate that this ability is not influenced by losses in decoding performance due to decoding across session boundaries. In order to test cross-session decoding, we thus trained the classifiers on all eight runs of the single trial condition in session 1 and tested their decoding performance on the ninth run of the single trial condition in session 2. Classifiers trained on data from all nine runs of the single trials were subsequently applied to data from on-task intervals in sequence trials in session 2. For the classification analyses in on-task intervals of the sequence task, classifiers were trained on the peak activation patterns from all correct single trials (including session 1 and session 2 data) and then tested on all TR corresponding to the sequence task ITIs.

Feature selection. All participant-specific anatomical masks were created based on automated anatomical labeling of brain surface reconstructions from the individual T1w reference image created with Freesurfer's `recon-all` (42) as part of the `fMRIPrep` workflow (19), in order to account for individual variability in macroscopic anatomy and to allow reliable labeling (57, 58). For the anatomical masks of occipito-temporal regions we selected the corresponding labels of the cuneus, lateral occipital sulcus, pericalcarine gyrus, superior parietal lobule, lingual gyrus, inferior parietal lobule, fusiform gyrus, inferior temporal gyrus, parahippocampal gyrus, and the middle temporal gyrus (cf. 17, 59). For the anatomical masks of the hippocampal ROI, we selected the corresponding labels of the left and right hippocampus. For the anatomical ROI of motor cortex, we selected the labels of the left and right gyrus precentralis as well as gyrus postcentralis. The labels of each ROI are listed in Table S1. Only gray-matter voxels were included in the generation of the masks as BOLD signal from non-gray-matter voxels cannot be generally interpreted as neural activity (60). Note, however, that due to the whole-brain smoothing performed during preprocessing, voxel activation from brain regions outside the anatomical mask but within the sphere of the smoothing kernel might have entered the anatomical mask (thus, in principle, also including signal from surrounding non-gray-matter voxels).

Statistical analyses. All statistical analyses were run inside a Docker software container or, if analyses were executed on a high-performance computing cluster, a Singularity version of the same container (26, 27). All main statistical analyses were conducted using LME models employing the `lmer` function of the `lme4` package (version 1.1.27.1, 61) in `R` (version 4.1.2, 62). If not stated otherwise, all models were fit with participants considered as a random effect on both the intercept and slopes of the fixed effects, in accordance with results from (63) who recommend to fit the most complex model consistent with the experimental design. If applicable, explanatory variables were standardized to a mean of zero and a standard deviation of one before they entered the models. If necessary, we removed by-participant random slopes from the random effects structure to achieve a non-singular fit of the model (63). Models were fitted using the BOBYQA optimizer (64, 65) with a maximum of $500,000$ function evaluations and no calculation of gradient and Hessian of nonlinear optimization solution. The likelihoods of the fitted models were assessed using Type III ANOVA with Satterthwaite's method. A single-step multiple comparison procedure between the means of the relevant factor levels was conducted using Tukey's HSD test (66), as implemented in the `emmeans` package in `R` (version 1.7.0, 62, 67). In all other analyses, we used one-sample t-tests if group data was compared to a baseline or paired t-tests if two samples from the same population were compared. If applicable, correction for multiple hypotheses testing was performed using the false discovery rate (FDR) (68) or Bonferroni (69) correction method. If not stated otherwise, the α-level was set to $\alpha = 0.05$, and analyses of response times included data from correct trials only. When effects of stimulus transitions during sequence trials were analyzed, data from the first trial of each run and the first trial after the change in transition structure in the third task run were removed.

Statistical analyses of behavioral data. In order to test the a-priori hypothesis that behavioral accuracy in each of the nine runs of the single trials and five runs of the sequence trials would be higher than the chance level, we performed a series of one-sided one-sample t-tests that compared participants' mean behavioral accuracy per run against the chance level of $100\%/6 = 16.67\%$ (Fig. S5). Participants' behavioral accuracy was calculated as the proportion of correct responses per run (in %). The effect sizes (Cohen's d) were calculated as the difference between the mean of behavioral accuracy scores across participants and the chance baseline (16.67%), divided by the standard deviation of the data (70). The resulting p-values were adjusted for multiple comparisons using the Bonferroni correction (69).

To examine the effect of task run on behavioral accuracy (Fig. S5b) and response times (main text Fig. 2b) in sequence trials, we conducted an LME model that included all five runs of sequence trials as a numeric predictor variable (runs 1 to 5) as the main fixed effect of interest as well as by-participant random intercepts and slopes.

Analyzing the effect of one-step transition probabilities on behavioral accuracy (main text Fig. 2c) and response times (main text Fig. 2d), we conducted two-sided paired t-tests comparing the effect of high vs. low transition probability separately

for both unidirectional ($p_{ij} = 0.7$ vs. $p_{ij} = 0.1$) and bidirectional ($p_{ij} = 0.35$ vs. $p_{ij} = 0.1$) data. Effect sizes (Cohen's d) were calculated by dividing the mean difference of the paired samples by the standard deviation of the difference (70) and p-values were adjusted for multiple comparisons across both graph conditions and response variables using the Bonferroni correction (69).

In order to examine the effect of node distance on response times in sequence trials (main text Fig. 2e), we conducted separate LME models for data from the unidirectional and bidirectional graph structures. For LME models of response time in unidirectional data, we included a linear predictor variable of node distance (assuming a linear increase of response time with node distance; see main text Fig. 1f, top right) as well as random intercepts and slopes for each participant. The linear predictor variable was coded such that the node distance linearly increased from -2 to $+2$ in steps of 1, modeling the hypothesized increase of response time with node distance from 1 to 5 (centered on the node distance of 3). For LME models of response time in bidirectional data, we included a quadratic predictor variable of node distance (assuming an inverted U-shaped relationship between node distance and response time; see main text Fig. 1f, bottom right) as well as by-participant random intercepts and slopes. The quadratic predictor variable of node distance was obtained by squaring the linear predictor variable.

Statistical analyses of post-task questionnaire data. First, to analyze whether participants were able to express knowledge of the sequential ordering of stimuli and graph structure, we summed the number of participants who answered "yes" or "no" to the question whether they had noticed sequential ordering. Second, in those participants who indicated that they had noticed sequential ordering, we summed the number of participants in each run in which participants had noticed sequential ordering first. Third, as a sanity check, we split the sample by graph order (`uni - bi` vs. `bi - uni`) and calculated the mean transition probability ratings, categorizing transitions by their node distance in the two graph structures. In order to investigate the influence of graph structure (`uni` vs. `bi`), graph order (`uni - bi` vs. `bi - uni`) and sequence awareness (`yes` vs. `no`) on transition probability ratings, we modeled these factors in a LME model, see Equation 1.

$$\text{probability\_rating} \sim (\text{prob\_uni} + \text{prob\_bi}) \times \text{graph\_order} \times \text{sequence\_detected} + (1 \mid \text{Subject}) \quad [1]$$

To determine whether participants' ratings of pairwise transition probabilities were related to any of the two true graph structures (uni- and bidirectional graphs), we compared participants' ratings to that of a random guessing model. In this model, we generated 6-by-6 transition matrices with random transition probabilities sampled from a uniform distribution, excluding self-transitions. Sampling from other distributions (e.g., beta distribution) did not result in noticeable differences. We then computed the correlation (Pearson's r) between the generated matrices and the true transition matrices for the uni- and bidirectional graph structures, respectively (see Fig. S4a) and took the mean of the two correlation coefficients. This process was repeated 100,000 times to obtain the distribution of mean correlation coefficients under random guessing conditions. We then computed the 95th percentile of these mean correlation coefficients as an empirical threshold for identifying non-random transition probability ratings. Finally, we calculated the mean correlation coefficient between participants' pairwise ratings of transition probabilities and the transition matrices for the uni- and bidirectional graph. Probability ratings of participants with a correlation coefficient below the threshold were considered indistinguishable from random guessing.

modeling of response times based on the . We modeled SRs for each participant depending on the transitions that were experienced in the task, including training, single and sequence trials. Specifically, each of the six stimuli was associated with a vector that reflected a *running* estimate of the long-term visitation probability of all six stimuli, starting from the current node. The SR matrix $\mathbf{M}^t$ was therefore a 6-by-6 matrix that contained six predictive vectors, one for each stimulus, and changed over time (hence the index t). The SR matrix on the first trial was initialized with a baseline expectation of $\frac{1}{36}$ for each node. After a transition between stimuli s_t and s_{t+1}, the matrix row corresponding to s_t was updated following a temporal difference (TD) learning rule (71, 72) as follows

$$\mathbf{M}^t_{s_t,*} = \mathbf{M}^t_{s_t,*} + \alpha \left[\mathbf{1}_{s_{t+1}} + \gamma \mathbf{M}^t_{s_{t+1},*} - \mathbf{M}^t_{s_t,*} \right] \quad [2]$$

whereby $\mathbf{1}_{s_{t+1}}$ is a one-hot vector with a 1 in the ${s_{t+1}}^{\text{th}}$ position, $\mathbf{M}^t_{s_t,*}$ is the row corresponding to stimulus s_t of matrix $\mathbf{M}$. The learning rate α was arbitrarily set to a fixed value of $\alpha = 0.1$, and the discount parameter γ was varied in increments of 0.05 from 0 to 0.95, as also described in the main text. This meant that the SR matrix would change throughout the task to reflect the experienced transitions of each participant, first reflecting the random transitions experienced during the training and single trials, then adapting to the first experienced graph structure and later to the second graph structure in sequence trials. In order to relate the SR models to participants' response times, we calculated how surprising each transition in the sequence task was – assuming participants' expectations were based on the current SR on the given trial, $\mathbf{M}^t$. To this end, we normalized $\mathbf{M}^t$ to sum to 1, and then calculated the Shannon information (3) for each trial, reflecting how surprising the just observed transition from stimulus i to j was given the history of previous transitions up to time point t:

$$I(j) = -\log_2(\tilde{m}^t_{i,j}) \quad [3]$$

where $\tilde{m}^t_{i,j}$ is the normalized $(i,j)^{\text{th}}$ entry of SR matrix $\mathbf{M}^t$. Rare events are more more surprising and require more information to represent them than common events. Using the base-2 logarithm allowed to express the units of information in bits (binary digits) and the negative sign ensured that the information measure was always positive or zero. In this case, Shannon information will be zero when the probability of an event is 1.0 or a certainty, i.e., there is no surprise (3).

The final step in this analysis was to estimate LME models that tested how strongly this trial-wise measure of SR-based surprise was related to participants' response times in the sequence task, for each level of the discount parameter γ. LME models therefore included fixed effects of the SR-based Shannon surprise, in addition to nuisance factors of task run, graph order (uni – bi vs. bi – uni) and graph structure (uni vs. bi) of the current run, as well as by-participant random intercepts and slopes. Separate LME models were conducted for each level of γ (twenty values for γ from 0 to 0.95 in steps of 0.05), and model comparison of the twenty models was performed using AIC, as reported in the main text. To independently investigate the effects of graph condition (uni vs. bi) and graph order (uni – bi vs. bi – uni), we analyzed separate LME models for each combination of the two factors, using only SR-based Shannon surprise as the main fixed effect of interest, and including by-participant random intercepts and slopes (see Fig. S8).

Participant-specific SR model fitting. The above approach compared twenty LME models that were run across the data from all participants. In order to get an estimate of multi-step learning per participant, we performed model fitting to each participant's data. We therefore fit the SR model to each participant's data individually. Parameter fitting consisted of fitting only two parameters, namely the α and γ parameters of the SR model (see model description above). Model fitting minimized the negative log-likelihood of a GLM predicting response times (using an inverse gamma link function) within each participant using a nested approach akin to a coordinate descent approach (cf. 73, 74). Specifically, the parameters α and γ were set in an outer loop using non-linear search method (`NLOPT_GN_DIRECT_L`; 75) implemented in the `nloptr` package (76) in `R`. The GLM included a by-participant random intercept and regressors of the SR-based Shannon surprise, together with regressors for trial, task block, and the fingers used for responses. Note, that this modeling approach differed from the LME-based procedure described above, in that it used GLM with an inverse gamma link function and did not include additional regressors for trial and fingers used for the responses. The β coefficients of these regressors were then set using maximum likelihood estimation in an inner loop, and the resulting likelihood of the GLM was used to inform the non-linear search for the outer parameters (α and γ). Both the α and γ parameter were constrained to lie in the interval between 0.01 (lower bound) and 1 (upper bound). As shown in main text Fig. 3d, the best-fitting LME models of participants widely exhibited a significant relationship between Shannon surprise and response time on a trial, suggesting reliable estimates of the γ parameter. The value of the γ parameter directly influenced the Shannon surprise on each trial (see Eqns. 2 and 3). A non-significant relationship between response time and surprise (values above the dashed line) would indicate no effect on the model's likelihood and suggest unreliable estimates of the γ parameter which controls the surprise on each trial.

After fitting the participant-specific parameters (α and γ) of the SR model for each participant, we used these parameters to generate the SR matrix for each participant at every trial during the task. To this end, we used the individually fitted parameters for α and γ as an input to Equation 2 to generate trial-by-trial SR matrices for every participant during the sequence task. Note, that the model parameters were fit based on a model that included all behavioral task data including data from the single trials (for details, see above), resulting in one value per parameter per participant. An example of the resulting SR matrices over the time course of the sequence task is shown in main text Fig. 3g.

To investigate the relationship between `SR + 1-step` model parameters and the speed of multi-step learning (in contrast to one-step learning), for each participant, we calculated the mean response time (on correct trials only) per run of the sequence task (run 1 to 5), separately for trials with a high ($p_{ij} = 0.7$ and $p_{ij} = 0.35$) vs. low ($p_{ij} = 0.1$) transition probability. Next, we ran separate GLMs for each participant and probability level (high vs. low) to calculate the effect of learning (linear regressor of run 1 to 5) on mean response times. We then extracted the slope of this predictor separately for high vs. low probability transitions and calculated the difference between the two slopes for each participant. This yielded a quantification of how much faster participants learned to respond to high vs. low probability transitions in sequence trials. Finally, we calculated the correlation (Pearson's r) between this difference in learning slopes and the model parameter estimates separately for both α and γ across participants.

Following best practices (77), we quantitatively assessed the model fitting process by conducting full parameter recovery. We performed model parameter recovery by simulating Shannon surprise of the SR-model based on each participants fitted parameter α and γ, and converting the surprise estimate into response times using the same GLM used during fitting (i.e., inserting the beta and surprise estimates into the equation that links response times to surprise). The resulting simulated response times were then used in a model fitting procedure identical to that reported in the main text. As a measure of parameter recovery, we report the correlation (Pearson's r) between the input parameters and the recovered parameters. For a similar procedure, see (78).

Statistical analyses of classification accuracy and classifier probability time courses on single trials. In order to assess the classifiers' ability to differentiate between the neural activation patterns of individual visual objects and motor responses, we compared the predicted visual object or motor response for each example in the test set to the visual object or motor response that actually occurred on the corresponding trial. We obtained an average classification accuracy score for each participant by calculating the mean proportion of correct classifier predictions across all correctly answered single trials in Session 1 (main text Fig. 4a). The mean decoding accuracy scores of all participants were then compared to the chance baseline of $100\%/6 = 16.67\%$ using a one-sided one-sample t-test, testing the a priori hypothesis that mean classification accuracy would be higher than the chance baseline. The effect size (Cohen's d) was calculated as the difference between the mean accuracy score and the chance baseline, divided by the standard deviation of the data (70). These calculations were performed separately for each ROI, and the resulting p-values were adjusted for multiple comparisons using Bonferroni correction (69).

Furthermore, we assessed the classifiers' ability to accurately detect the presence of visual objects and motor responses on a

per-trial basis. For this analysis, we applied the trained classifiers to fifteen volumes, starting from the volume closest to event onset, and examined the time courses of probabilistic classification evidence in response to the event on single trials (main text Fig. 4b). To test whether the classifier probabilities reflected the expected increase for the event occurring on a given trial, we compared the time series of classifier probabilities for the classified class with the mean time courses of all other classes using a two-sided paired t-test at the fourth TR from event onset. Classifier probabilities were normalized by dividing each probability by the sum of classifier probabilities across all fifteen TRs of a given trial. To adjust for multiple comparisons of two observations (one test per ROI), we applied the Bonferroni correction method (69). In the main text, we report results for the peak classification probability of the true class, which corresponds to the fourth TR after stimulus onset. The effect size (Cohen's d) was calculated as the difference between the means of the probabilities of the current versus all other stimuli, divided by the standard deviation of the difference (70).

We also examined the effect of task run on classification accuracy in single trials. To this end, we conducted an LME model, including task run as the main fixed effect of interest, along with by-participant random intercepts and slopes. We then assessed whether performance was above chance level for all nine task runs by conducting nine separate one-sided one-sample t-tests (one per run) separately for each ROI, testing the a priori hypothesis that mean decoding accuracy would exceed the 16.67% chance level in each run. All p-values were adjusted for 18 multiple comparisons (across nine runs and two ROIs) using Bonferroni correction (69). The results are reported in the main manuscript and shown in Fig. S14.

Modeling stimulus-driven classifier time courses in on-task intervals. In our previous work (17), we showed that analyzing probabilistic classifier evidence on single trials of a presented stimulus revealed multivariate decoding time courses that can be characterized by a response function that resembles single-voxel hemodynamics. Here, we applied the same methodology to capture the expected effects of stimulus-driven activity elicited by previous trials in on-task intervals. The details of this modeling approach were first described in (17), but, for completeness, we reproduce them here as well.

Specifically, we modeled an individual classifier probability response function as a sine wave that was flattened after one cycle, scaled by an amplitude and adjusted to baseline. The model was specified as follows:

$$h(t) = \frac{A}{2}\sin(2\pi f t - 2\pi f d - 0.5\pi) + b + \frac{A}{2} \quad [4]$$

where A is the response amplitude (the peak deviation of the function from baseline), f is the angular frequency (unit: 1/TR, i.e., $1/1.25 = 0.8$ Hz), d is the onset delay (in TRs), and b is the baseline (in %). The restriction to one cycle was achieved by converting the sine wave in accordance with the following piecewise function:

$$H(t) = \begin{cases} h(t) & \text{if} d \leq t \leq (d + \frac{1}{f}) \\ b & \text{otherwise} \end{cases} \quad [5]$$

As in (17), we fitted the four model parameters (A, f, d and b) to the mean probabilistic classifier evidence of each stimulus class at every TR separately for each participant. For convenience, we count time t in TRs. The default parameters (as well as lower and upper bounds) were set to $A = 0.6$ ($0.1 \leq A \leq 1.0$), $f = 0.2$ ($0.01 \leq f \leq 0.5$), $d = 0.0$ ($0 \leq d \leq 8$), and $b = 0.1$ ($0.0 \leq b \leq 0.3$), respectively. The parameters were optimized using a version of the COBLYA algorithm for derivative-free optimization with nonlinear inequality and equality constraints (`NLOPT_LN_COBYLA`; 79) implemented in the `nloptr` package (76) in `R`. The relative tolerance for convergence was set to 1.0×10^{-8}. The maximum number of evaluations allowed during optimization was set to 1.0×10^{5}. This function was then fit to the mean classifier time courses across ten TRs on single trials, separately for each stimulus class, ROI, and participant.

To assess the accuracy of the sine-based response function in predicting observed data, we implemented an evaluation function, aiming to fine-tune model parameters for improved predictive performance. Specifically, the sum of squared errors was computed to quantify the disparity between the predicted values (y) and the observed data (see Equation 6). The sum of squared errors metric serves as the indicator of the model's fidelity to the observed data, with lower values indicative of a more accurate fit. The sum of squared errors output from this evaluation function guided the optimization algorithm in iteratively adjusting model parameters to minimize the disparity between predicted and observed data, ultimately refining the accuracy of the sine-based response model.

$$SSE = \sum_i (data_i - y_i)^2 \quad [6]$$

Next, we modeled stimulus-evoked classifier probabilities lagging into the on-task interval. To this end, we first took the response functions (described by the four model parameters A, f, d and b) that were fit individually to the classifier probability data on single trials of each participant, ROI and stimulus and convolved them with the onsets of ten stimuli *before* and ten stimuli *after* the on-task intervals in the sequence task. Given the timings of the trial procedure (see Methods above), we reasoned that considering ten stimuli before the on-task interval would sufficiently capture any stimulus-evoked activity leaking into the on-task interval. Specifically, with a fixed stimulus duration of 800 ms and an average ITI of 750 ms (for details, see the description of the trial procedure in the Methods above) this would amount to considering stimulus-driven activity of up to 15.5 s on average before the on-task interval, which roughly amounts to the expected duration of the canonical hemodynamic response function (see e.g., 80). We reasoned that additionally considering stimuli *after* the on-task interval would allow to account for and investigate any combination of stimulus-evoked and replay-driven activity that might extend beyond the

on-task intervals. Of note, due to trial-specific timing of task components, not all trials had all stimuli occur before them, i.e., for some trials some stimuli never occurred in the ten preceding trials.

We then calculated the mean stimulus-evoked activity for each TR of the on-task interval separately for each participant. Note, that the sine-based response model is a continuous function that can be evaluated over any arbitrarily densely sampled interval. Of note, the average value of a continuous function on an interval f_{avg} can be approximated by a list of points to derive that the average value is proportional to the area under the curve, i.e., the definite integral:

$$f_{\text{avg}} = \frac{1}{b-a}\int_a^b f(x)\,dx \qquad [7]$$

Here, as a result of our model fitting procedure (see above) we evaluated the function across a time window of 10 TRs with a constant sampling frequency of 0.1. Note, that increasing the sampling frequency (e.g., to 0.01 or 0.001) would only marginally improve the approximation of the mean of the continuous function within a certain interval at the expense of increased computation time. As the same stimulus could occur multiple times on the interval-preceding trials, this might result in overlapping activation of the same stimulus. To account for overlapping activation from the same stimulus, we summed the modeled activation values separately for each stimulus at each TR. To obtain one activation value per TR, we considered all data points that would fall within the range of one TR of 1.25 s. When a specific TR did not contain any stimulus-related activity according to the modeling approach, the activation of that stimulus at that time point was set to 0. An illustration of the resulting modeled stimulus-driven classifier probability time courses is shown in Fig. S16.

Sequenceness Analysis. In our approach (for details, see 17), sequentiality of neural replay in fMRI is indexed by the ordering of multivariate classifier time courses within a *single* TR. This analysis approach is illustrated in Fig. S18 and described in the following: First, we apply trained classifiers to a series of volumes (TRs) where we expect replay (or any other sequential neural activity) to occur. In the current design, these are the 10 s ITIs during sequence trials (corresponding to roughly eight TRs at a TR of 1.25 s), where we expect online replay to occur. Of note, the method can be applied to any number of TRs in fMRI data, including extended resting-state periods. As we use *probabilistic* classifiers, we obtain a time course of classifier probabilities for each class across the relevant time window (see Fig. S18a). The core assumption of our analysis approach is that any sequentiality in the neural signal that is elicited by a replay sequence will systematically translate into the corresponding classifier time courses in a speed-dependent manner, even long after the replay event is over (an assumption that we have confirmed empirically in 17). Next, we conduct a TR-wise linear regression between the serial positions of the sequence events and their classification probabilities and obtain the slope of the regression as an index of linear association. Here, if earlier sequence events have a higher classification probability compared to later sequence events, the slope coefficient will be positive (Fig. S18b). In contrast, if later sequence events have a higher classification probability compared to earlier sequence events, the slope coefficient will be negative (Fig. S18c). Note that, for convenience, we flip the sign of the regression slopes so that positive values indicate forward ordering and negative values indicate backward ordering. Critically, in our previous work (17), we found that if we then analyze the time course of this sequentiality metric across multiple successive TRs, we find that the same neural replay sequence results in both forward and backward sequentiality in the corresponding fMRI classifier time courses (see 17, their Figure 3). In other words, in earlier TRs, classifier probabilities have the same order as the preceding neural event sequence, while the ordering of classifier probabilities at later TRs indicates reverse ordering relative to the true event sequence.

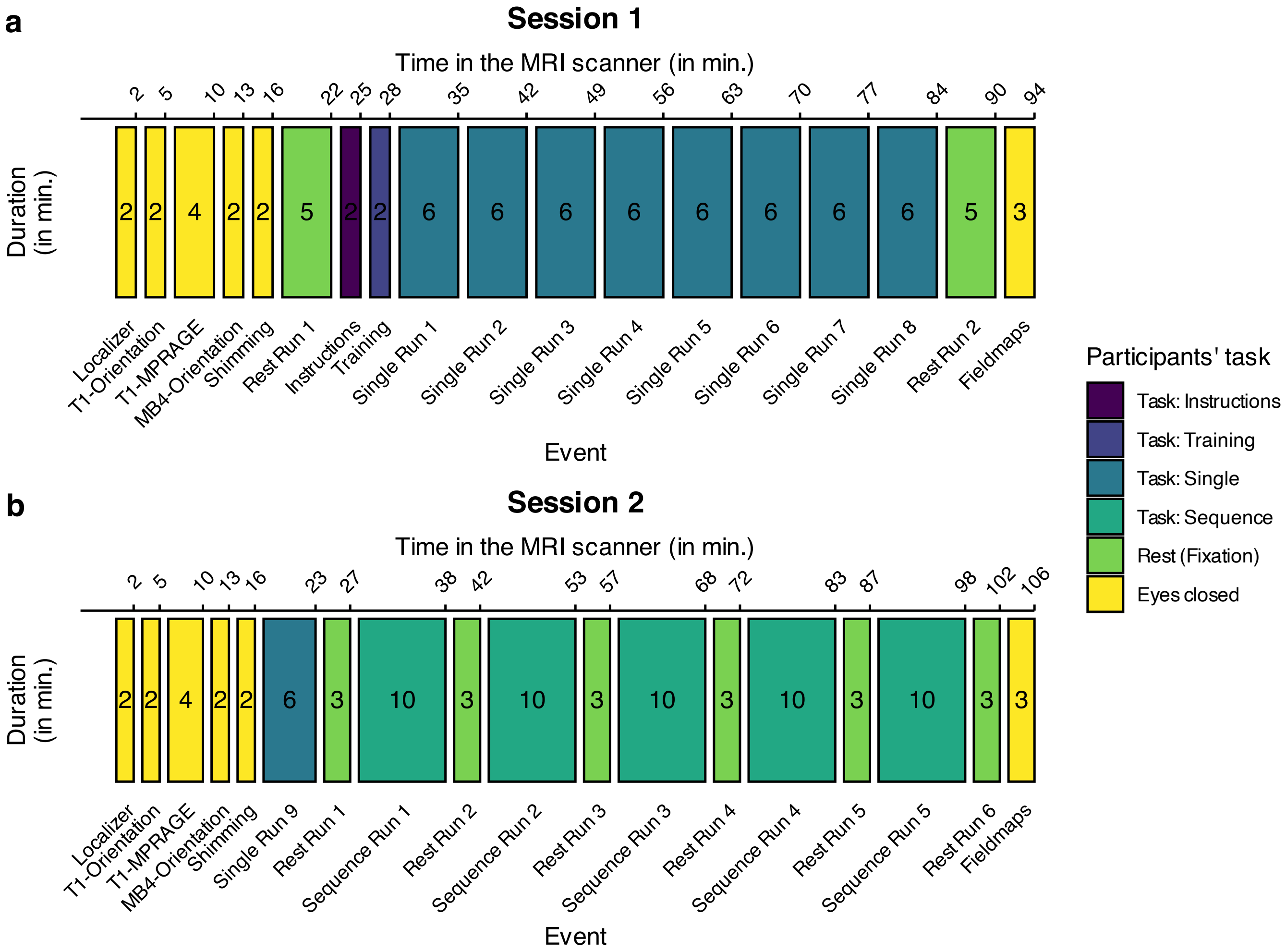

Fig. S1. Study procedure. (a) Session 1 started with a 5 min resting-state scan before participants read the task instructions and completed the training condition of the task. Participants then completed eight runs of the single trial condition of about 6 min each before another 5 min resting-state scan was recorded. **(b)** Session 2 started with another run of the single trial condition of about 6 min. Participants then completed all five runs of the sequence task of about 10 min each which were interleaved with six resting-state scans of 3 min each. Both experimental sessions started with a short localizer scan, a T1w anatomical MRI scan as well as advanced shimming and ended with the recording of fieldmaps (for details on MRI acquisition, see SI Methods). Participants were asked to keep their eyes closed during these scans and other additional preparations by the study staff, e.g., orientation of the FOV. The numbers inside the rectangles indicate approximate duration of each procedure in minutes. Colors indicate participants' task during each study event (see legend). All timings are rough estimates based on the task design and may have slightly varied within and between individual participants.

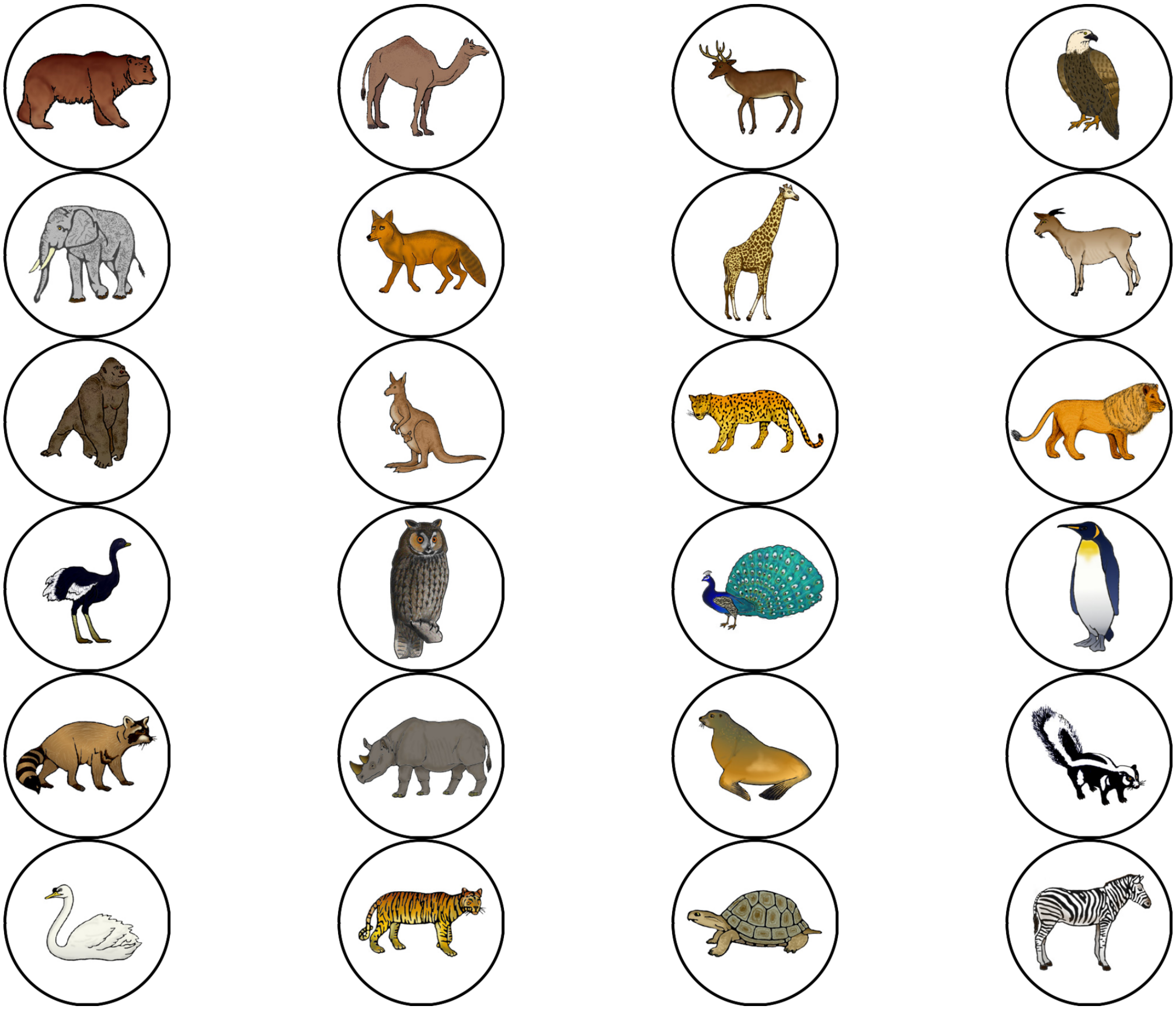

Fig. S2. Overview of all 24 animal stimuli. All visual stimuli were taken from a set of colored and shaded images commissioned by (7), which are loosely based on images from the original Snodgrass and Vanderwart set (8). The images are freely available from the internet at https://sites.google.com/andrew.cmu.edu/tarrlab/stimuli under the terms of the Creative Commons Attribution-NonCommercial-ShareAlike 3.0 Unported license (CC BY-NC-SA 3.0; for details, see https://creativecommons.org/licenses/by-nc-sa/3.0/) and have been used in similar previous studies (e.g., 9). Stimulus images courtesy of Michael J. Tarr, Carnegie Mellon University, (for details, see http://www.tarrlab.org/). In total, we selected 24 images which depicted animals that could be expected in a public zoo. Specifically, the images depicted a bear, a dromedary, a deer, an eagle, an elephant, a fox, a giraffe, a goat, a gorilla, a kangaroo, a leopard, a lion, an ostrich, an owl, a peacock, a penguin, a raccoon, a rhinoceros, a seal, a skunk, a swan, a tiger, a turtle, and a zebra (in alphabetical order, from left to right and top to bottom). For each participant, six task stimuli were randomly selected from the set of 24 the animal images and each image was randomly assigned to one of six response buttons. For more details, see the task description in the SI Methods.

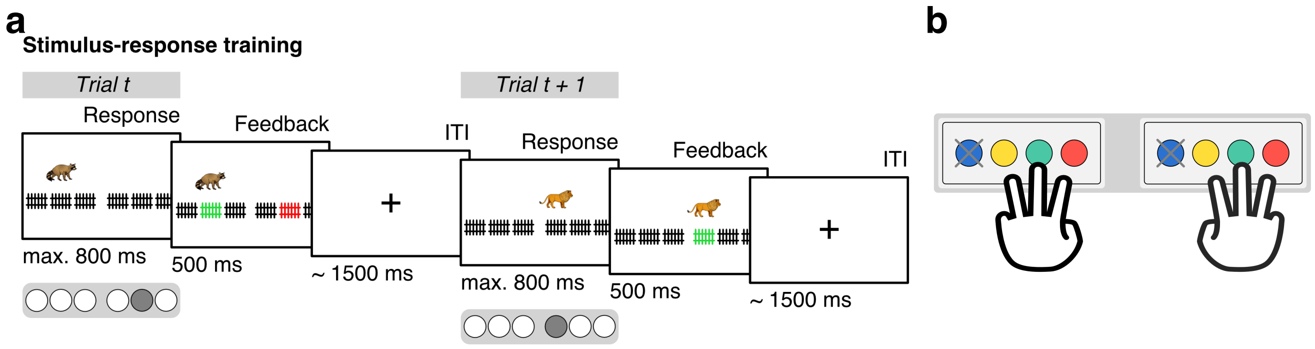

Fig. S3. Stimulus-response training and response mapping. (a) On training trials, participants were explicitly trained to learn associations between six animal stimuli and six response buttons. On each trial, an animal appeared above one of six cages that were representing the six response buttons. Participants were asked to press the corresponding response button within 800 ms and subsequently received feedback whether their response was incorrect (see *Trial t*) or correct (see *Trial t + 1*). fMRI data from training trials were not used for any further analysis. For more details, see the task description in the SI Methods. **(b)** Hand placement during behavioral responses in the MRI scanner. Each of the six animals was associated with one of six response buttons that participants controlled with their index, middle, and ring fingers of both hands. The response pad included a fourth button that participants were asked to ignore. Colors indicate the actual color of each button and are not related to any color in the task or figures in the main text. The illustration of the hands in (b) is an artwork "three fingers" by Herbert Spencer from the Noun Project, licensed under Creative Commons Attribution 3.0 United States (CC BY 3.0 US; for details, see https://creativecommons.org/licenses/by/3.0/us/) and available from https://thenounproject.com/term/three-fingers/155721/. All visual stimuli in (a) were taken from a set of colored and shaded images commissioned by (7), which are loosely based on images from the original Snodgrass and Vanderwart set (8). The images are freely available from the internet at https://sites.google.com/andrew.cmu.edu/tarrlab/stimuli under the terms of the Creative Commons Attribution-NonCommercial-ShareAlike 3.0 Unported license (CC BY-NC-SA 3.0; for details, see https://creativecommons.org/licenses/by-nc-sa/3.0/) and have been used in similar previous studies (e.g., 9). Stimulus images courtesy of Michael J. Tarr, Carnegie Mellon University, (for details, see http://www.tarrlab.org/).

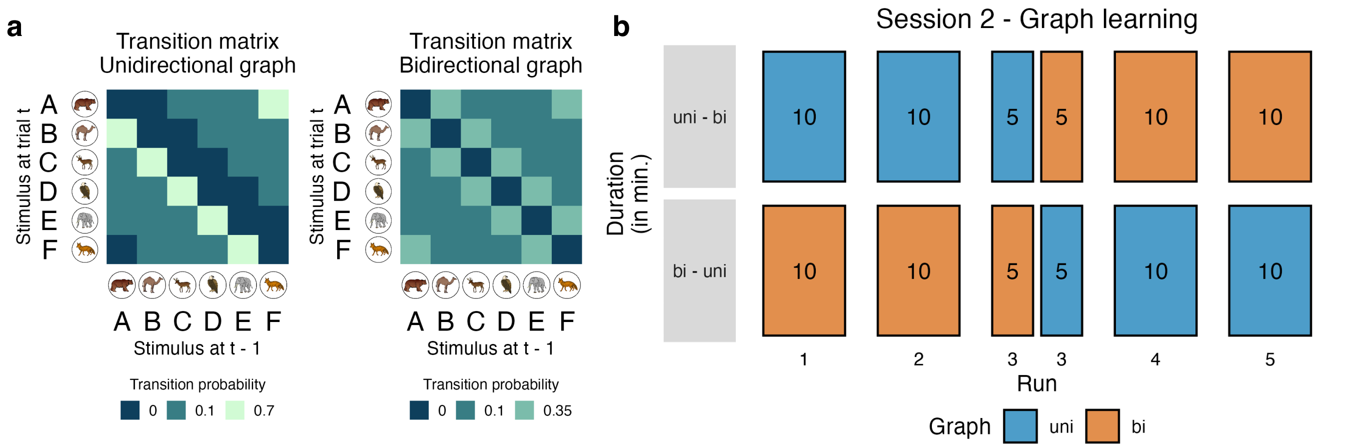

Fig. S4. Transition matrices and graph structure change (a) Transition matrices of the unidirectional (left) and bidirectional (right) graph structures. Each transition matrix depicts the probability (colors; see legend) of transitioning from the stimulus at the previous trial $t-1$ (x-axis) to the current stimulus at trial t (y-axis). **(b)** Within-participant order of the two graph structures across the five runs of the sequence trials. $n = 12$ participants first experienced the unidirectional, then the bidirectional graph structure (uni – bi; top horizontal panel) while $n = 27$ participants experienced the reverse order (bi – uni; bottom horizontal panel). In both groups of participants, the graph structure was changed without prior announcement halfway through the third task run of sequence trials. Numbers indicate approximate run duration in min. Colors indicate graph condition (uni vs. bi; see legend).

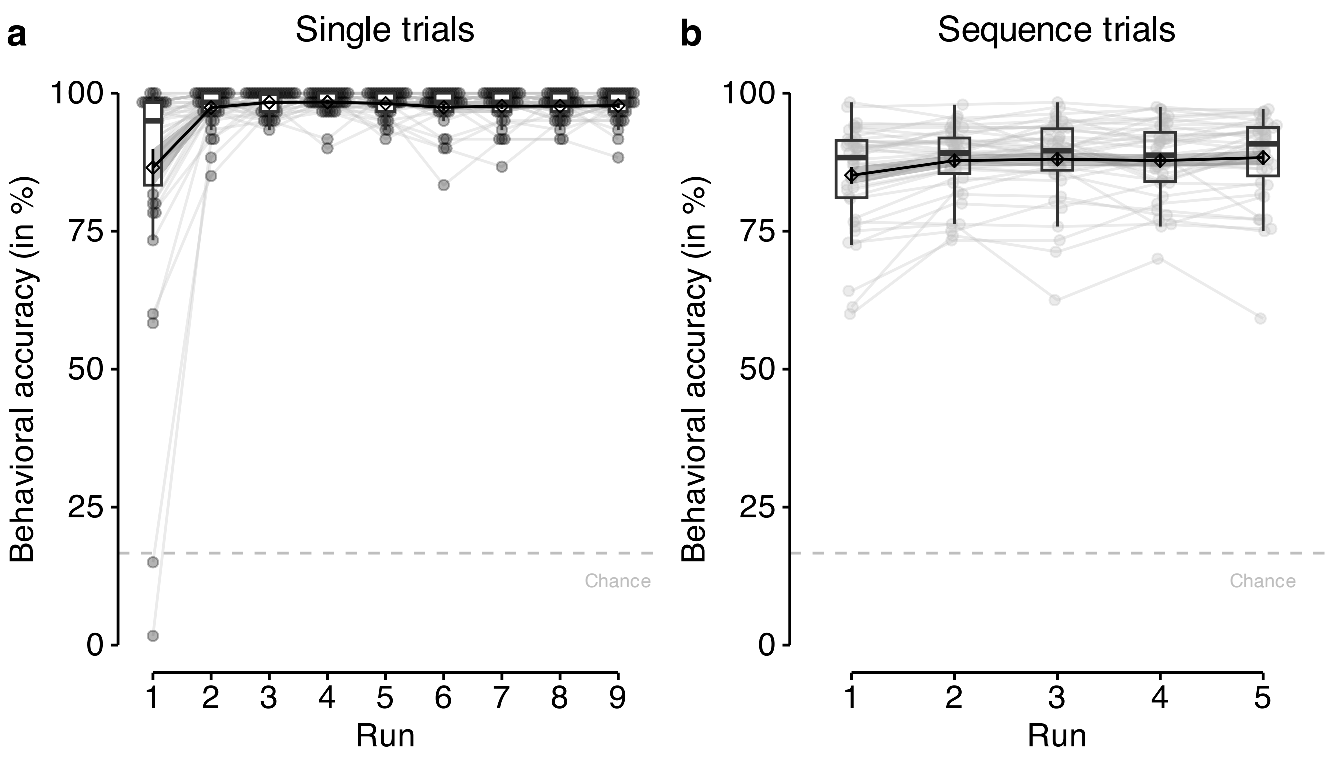

Fig. S5. Behavioral accuracy per task run in single and sequence trials. (a) Mean behavioral accuracy (in %; y-axis) per run (x-axis) in single trials. **(b)** Mean behavioral accuracy (in %; y-axis) per run (x-axis) in sequence trials. The chance level (gray dashed line) is at 16.67%. Each gray dot corresponds to averaged data from one participant. Gray lines connect data across runs for each participant. Boxplots indicate the median and IQR. The lower and upper hinges correspond to the first and third quartiles (the 25th and 75th percentiles). The upper whisker extends from the hinge to the largest value no further than $1.5*$ IQR from the hinge (where IQR is the inter-quartile range, or distance between the first and third quartiles). The lower whisker extends from the hinge to the smallest value at most $1.5*$ IQR of the hinge. The diamond shapes show the sample mean. Error bars and shaded areas indicate ± 1 SEM. All statistics have been derived from data of $n = 39$ human participants who participated in one experiment.

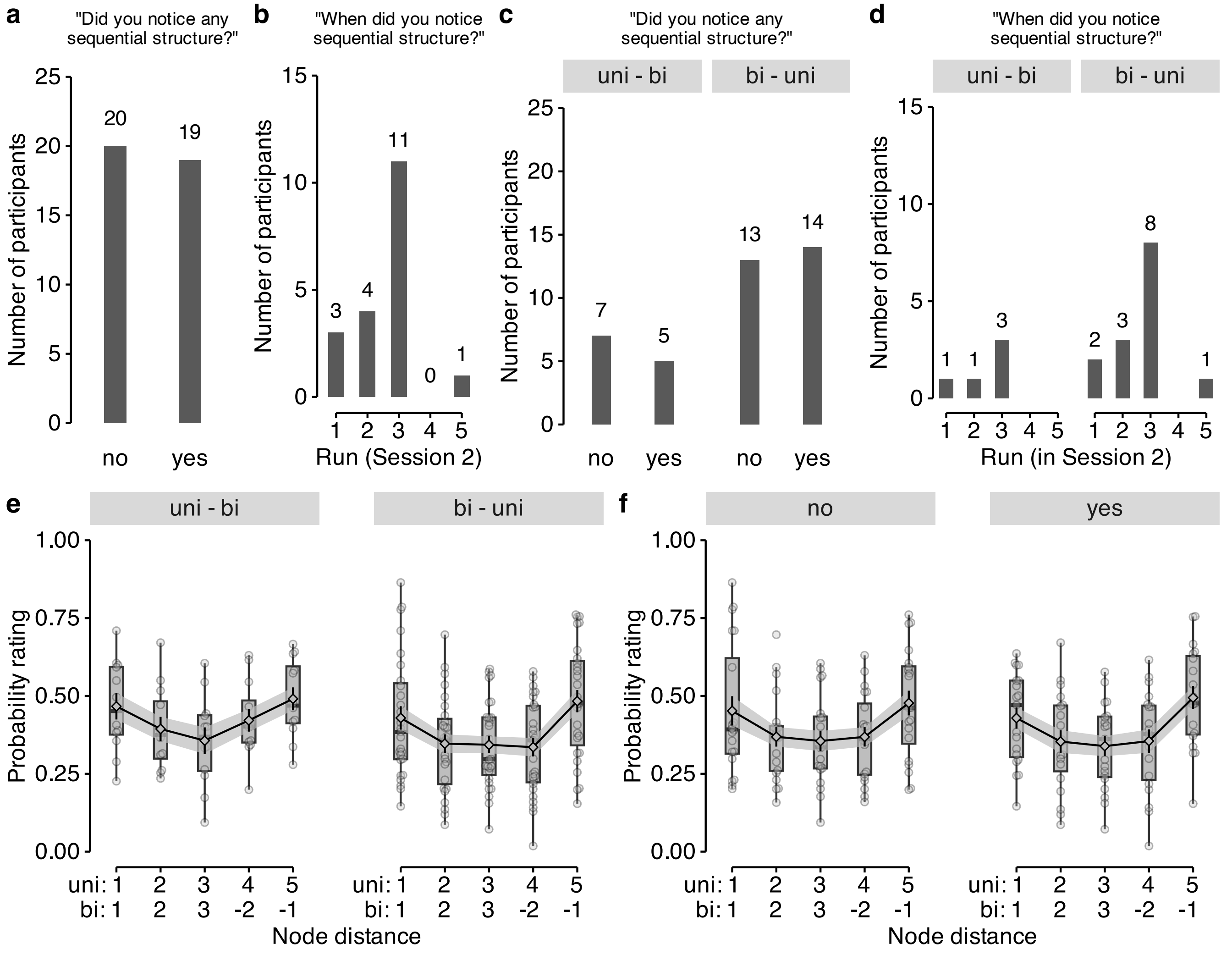

Fig. S6. Responses to post-task questionnaire. **(a)** Number of participants (y-axis) who noticed sequential structure in the task (x-axis). **(b)** Number of participants (y-axis) who detected sequential structure ($n = 19$, see panel (a)) indicating in which run of sequence trials (x-axis) they first noticed it. **(c)** As in (a), split by graph order (uni – bi vs. bi – uni; panels). **(d)** As in (b), split by graph order as in (c). **(e)** Ratings of pairwise transition probabilities (in %; y-axis) by node distance (x-axis), split by graph order as in (c). **(f)** As in (e), split by explicit awareness as in (a). Boxplots indicate the median and IQR. Diamond shapes show the sample mean. Error bars and shaded areas denote ± 1 SEM. Each dot corresponds to data from a single participant.

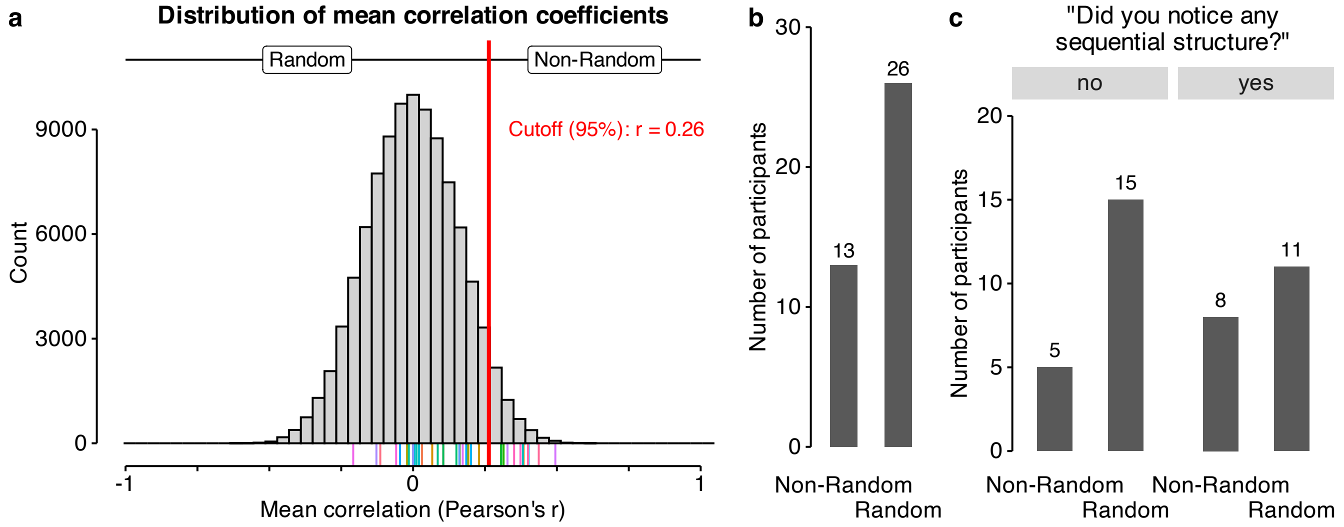

Fig. S7. Random guessing model to classify transition probability ratings. **(a)** Distribution of mean correlation coefficients between randomly generated transition matrices and the two graph transition matrices (uni- and bidirectional; cf. Fig. S4a). The histogram shows the frequency of mean correlation coefficients across 100,000 simulations. The red line represents the 95th percentile cutoff, indicating the threshold above which correlations are considered related (or non-random) with respect to the two graph structures. Each colored line at the bottom corresponds to data from a single participant. **(b)** Number of participants (y-axis) whose transition probability ratings were classified as non-random or random based on the random guessing model (x-axis) **(c)** As in (b), split by binary post-task report of explicit sequence awareness ("Did you notice any sequential structure?"; panels).

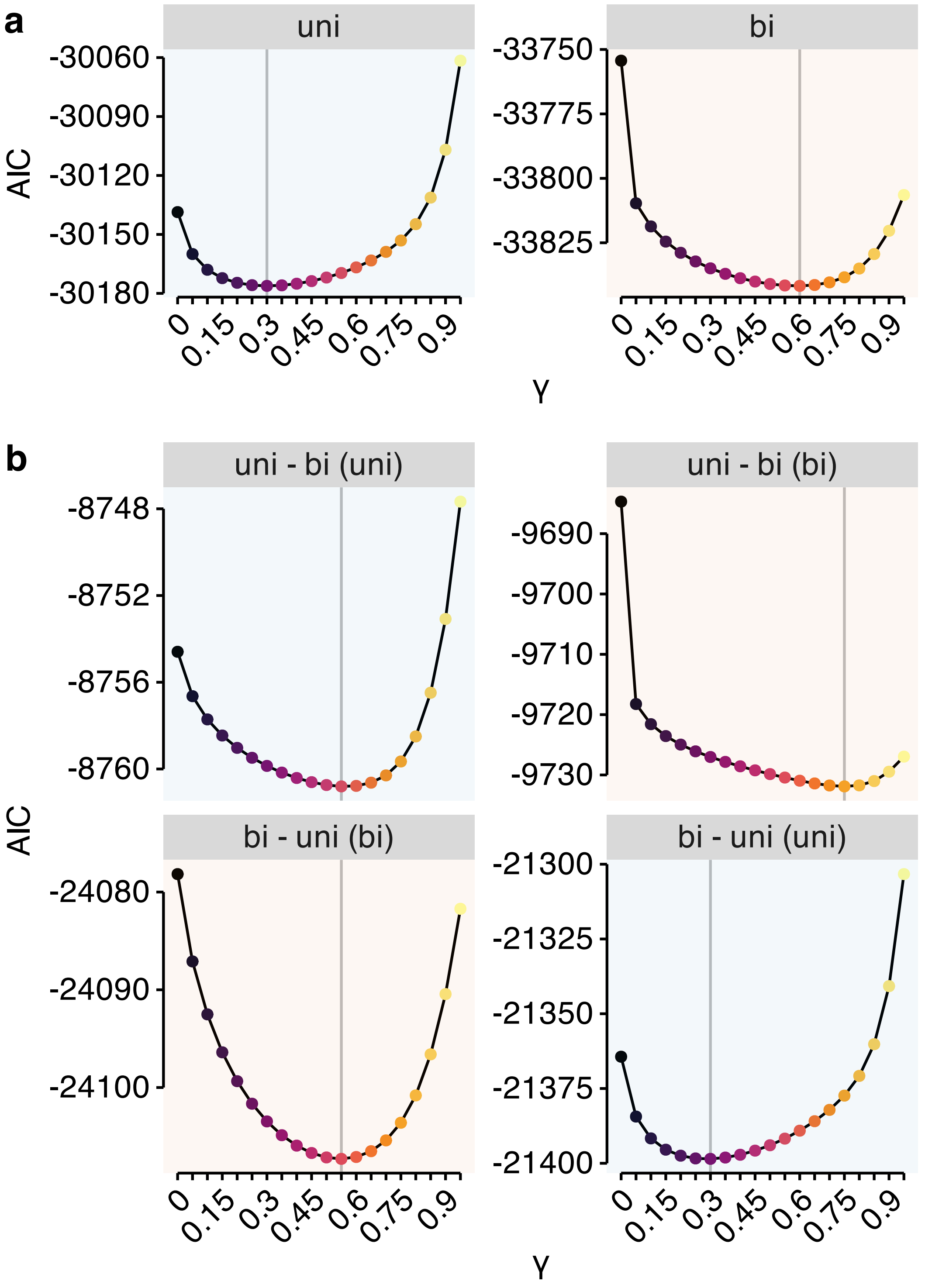

Fig. S8. Influence of graph condition and order on SR-based modeling of response times. (a) AIC scores (y-axis) for LME models fit to participants' log response time data using Shannon information based on SRs with varying predictive horizons (the discounting parameter γ; x-axis) as the predictor variable, separated by graph condition (uni vs. bi). **(b)** AIC scores (y-axis) for LME models fit to participants' log response time data using Shannon information based on SRs with varying predictive horizons (the discounting parameter γ; x-axis) as the predictor variable, separated by graph order (uni – bi vs. bi – uni; horizontal panels) and graph condition (uni vs. bi; panel colors). Vertical lines in (a) and (b) mark the lowest AIC score. All statistics have been derived from data of $n = 39$ human participants who participated in one experiment.

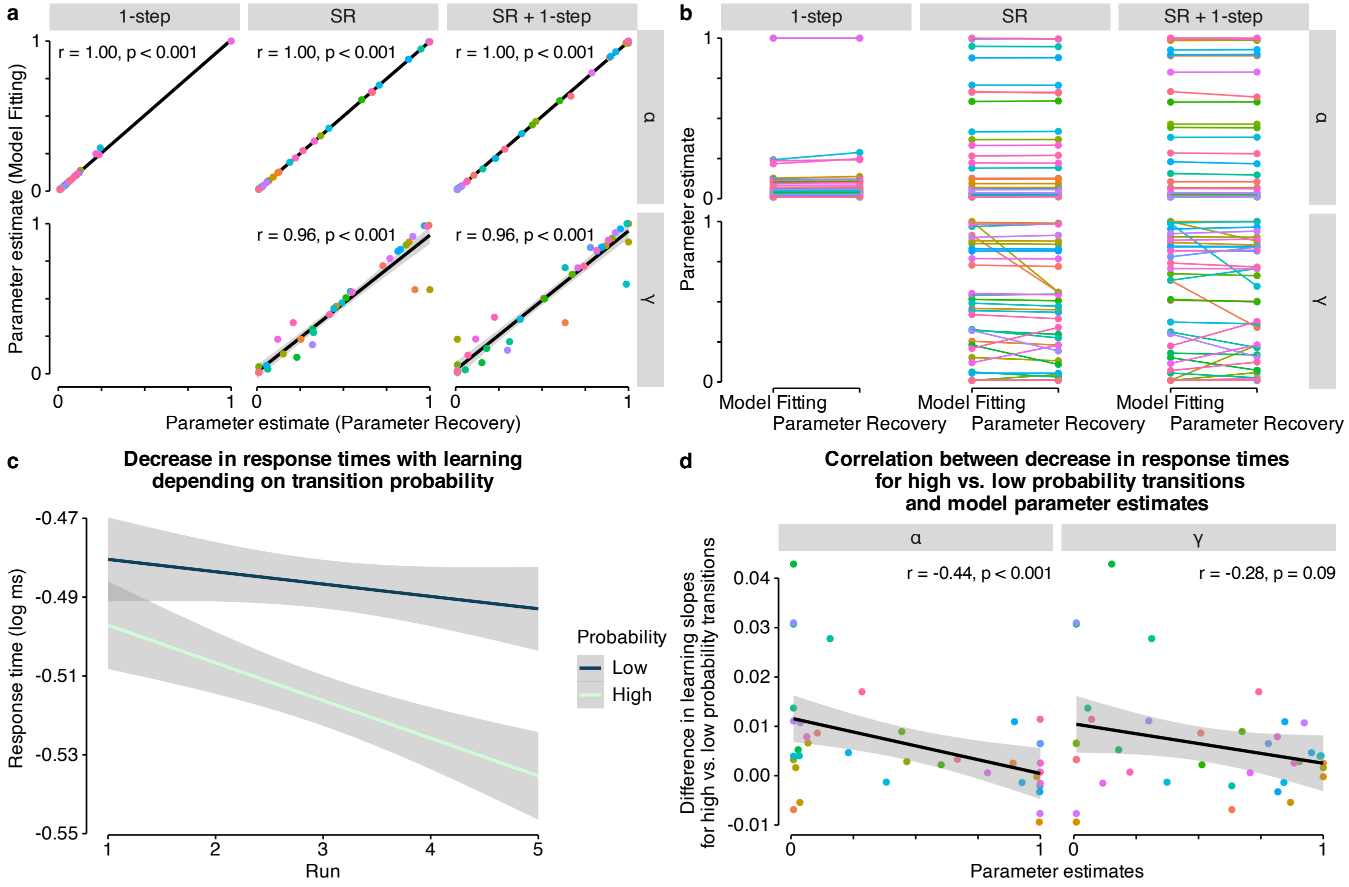

Fig. S9. Parameter recovery and relationship between parameters and speed of learning. (a) Relationship between parameters recovered from model fitting procedure on simulated response time data (x-axis) and input parameters used for simulation (y-axis), separately for each model (vertical panels) and model parameter (horizontal panels). Shaded areas indicate ± 1 SEM. **(b)** Relationship between parameters recovered from model fitting procedure on simulated response time data and input parameters used for simulation for each participant (colored dots and lines), separately for each model (vertical panels) and model parameter (horizontal panels). **(c)** Mean slope of log response time (y-axis) per run (x-axis) in sequence trials, separately for high- vs. low probability transitions (colors). **(d)** Correlation between participant-specific best-fitting parameter estimates in the `SR + 1-step` model (x-axis) and the difference in learning slopes for high- vs. low probability transitions (y-axis), separately for the α and γ model parameters (panels). Each dot corresponds to data from a single participant. All statistics have been derived from data of $n = 39$ human participants who participated in one experiment.

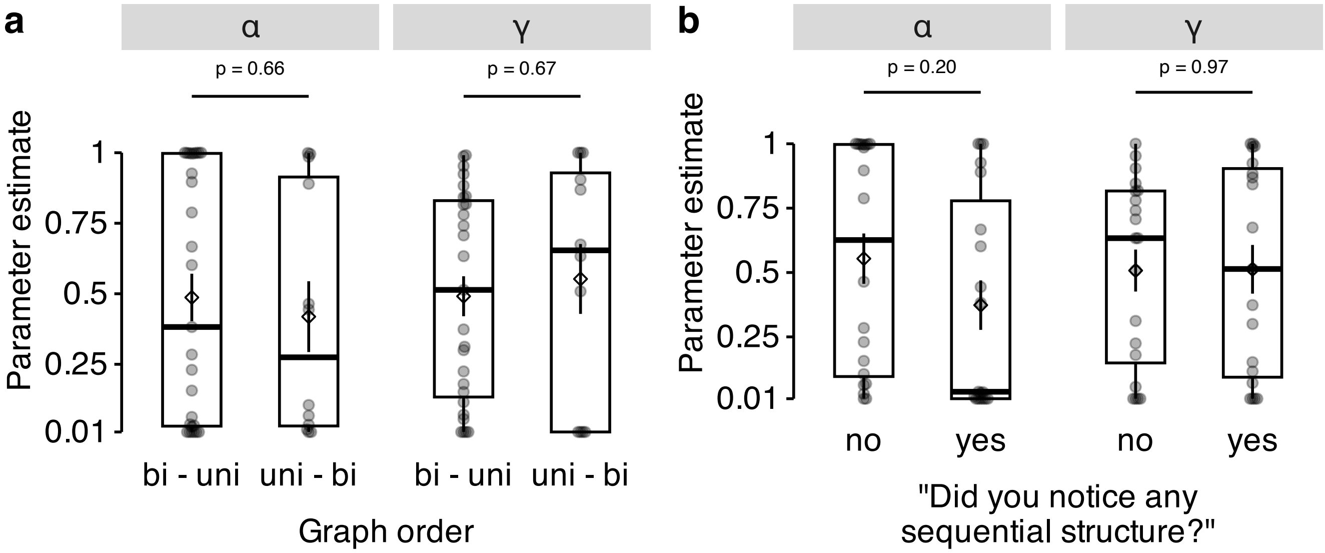

Fig. S10. SR parameter estimates split by graph order and explicit sequence awareness. (a) Model parameter estimates for α and γ (panels; interval: $[0.01, 1.0]$) in the `SR + 1-step` after participant-specific model fitting, separated by the graph order that the participant experienced (uni–bi vs. bi–uni). **(b)** Parameter estimates as in (a) but separated by explicit sequence awareness ("yes" vs. "no"). Boxplots indicate the median and inter-quartile range. The lower and upper hinges correspond to the first and third quartiles (the 25th and 75th percentiles). The upper whisker extends from the hinge to the largest value no further than $1.5*$ IQR from the hinge (where IQR is the inter-quartile range, or distance between the first and third quartiles). The lower whisker extends from the hinge to the smallest value at most $1.5*$ IQR of the hinge. Diamond shapes show the sample mean. Error bars indicate ± 1 SEM. Each dot corresponds to averaged data from one participant. All statistics have been derived from data of $n = 39$ human participants who participated in one experiment.

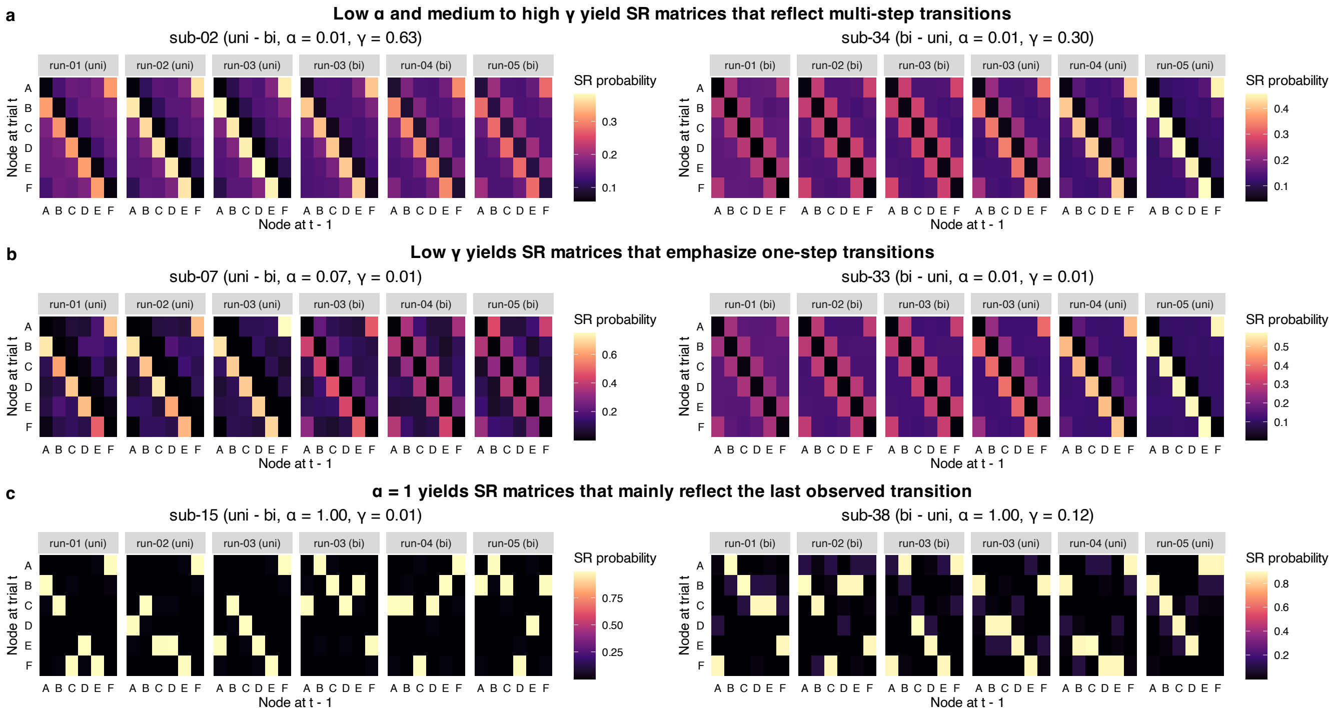

Fig. S11. Example SR matrices of participants with extreme model parameter estimates. In each panel, SR matrices for two participants were selected who experienced the two graph structures in `uni - bi` (left column) or `bi - uni` (right column) order, respectively, separately for each combination of run and graph structure in sequence trials (panels) at the last trial of the respective task run. Colors indicate the normalized expected future visitation of each of the six nodes in the graph structure according to Equation 2 (see main text). SR matrices were determined based on individually fitted parameters for α and γ (see plot titles). Note, that the third run included the change in graph structures halfway through the run and the data is therefore shown separately for the two halves of the run.

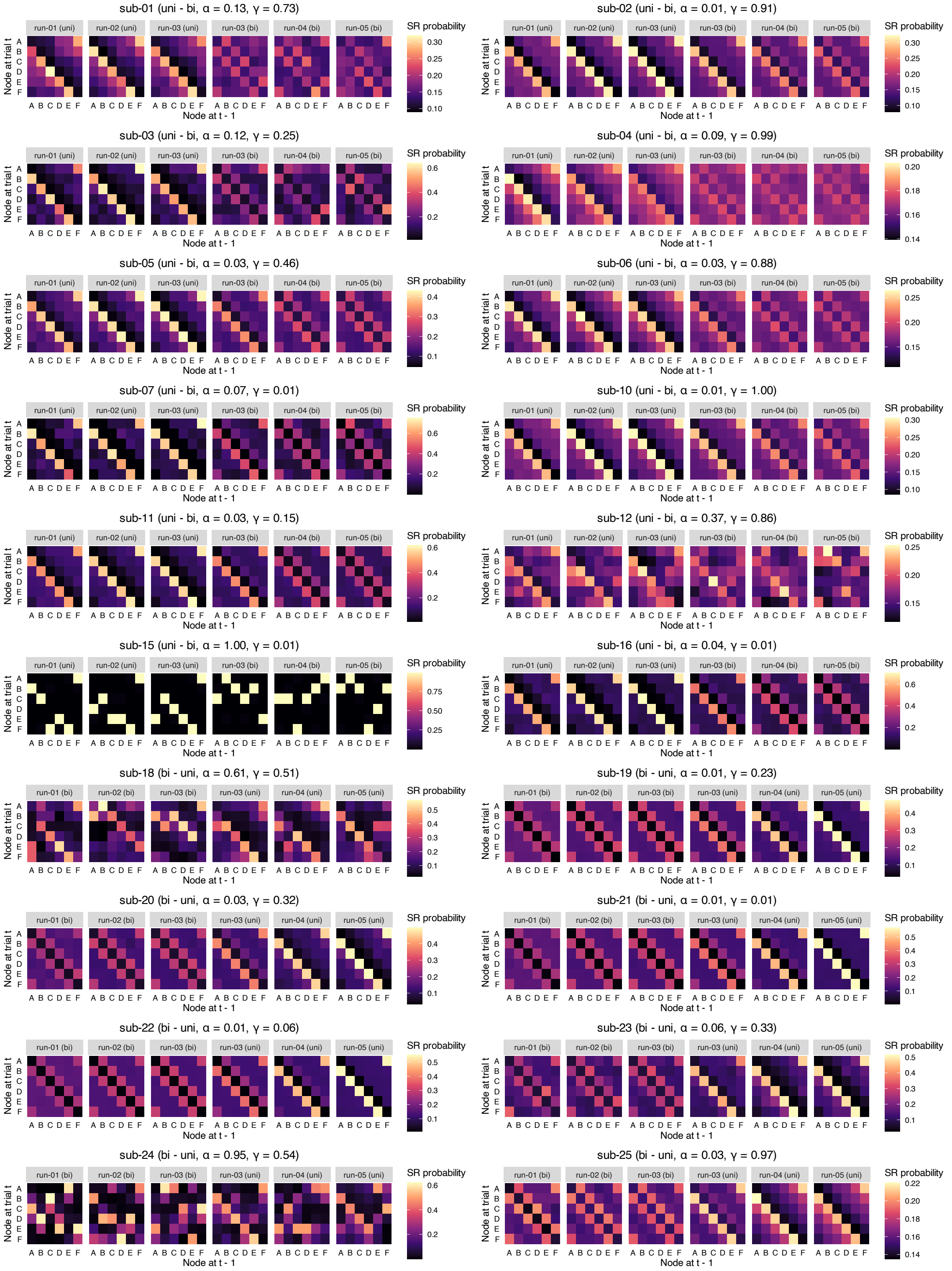

Fig. S12. SR matrices of participants 1-25. SR matrices for two selected participants who experienced the two graph structures in `uni - bi` (top panel) or `bi - uni` (bottom panel) order, respectively, separately for each combination of run and graph structure in sequence trials (panels) at the last trial of the respective task section. The colors indicate the normalized expected future visitation of each of the six nodes in the graph structure according to Equation 2. SR matrices were determined based on individually fitted parameters for α and γ (see plot titles). Note, that the third run included the change from one to the other graph structure and the data is therefore shown separately for the two halves of the run.

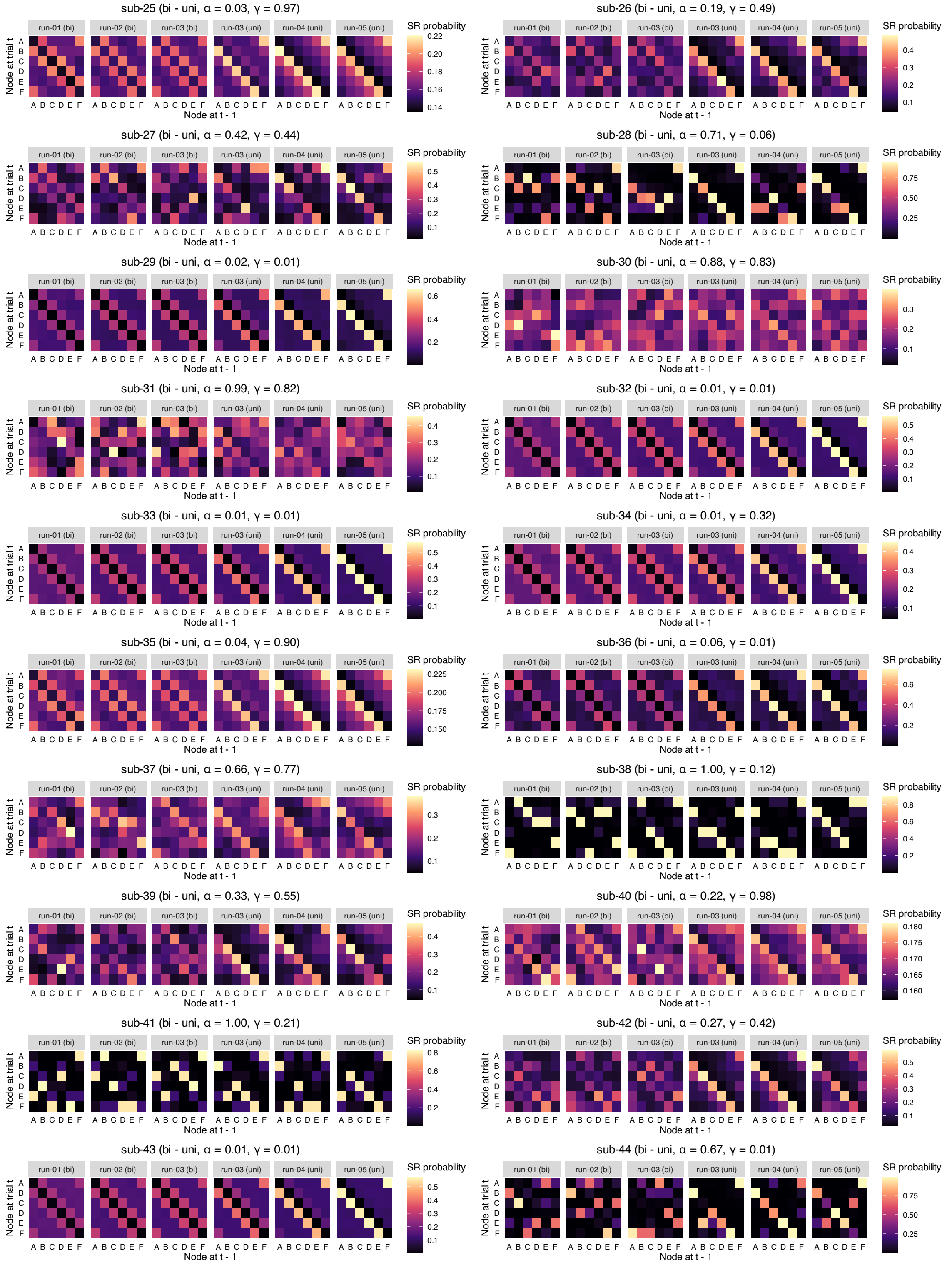

Fig. S13. SR matrices of participants 25-44. SR matrices for two selected participants who experienced the two graph structures in `uni – bi` (top panel) or `bi – uni` (bottom panel) order, respectively, separately for each combination of run and graph structure in sequence trials (panels) at the last trial of the respective task section. The colors indicate the normalized expected future visitation of each of the six nodes in the graph structure according to Equation 2. SR matrices were determined based on individually fitted parameters for α and γ (see plot titles). Note, that the third run included the change from one to the other graph structure and the data is therefore shown separately for the two halves of the run.

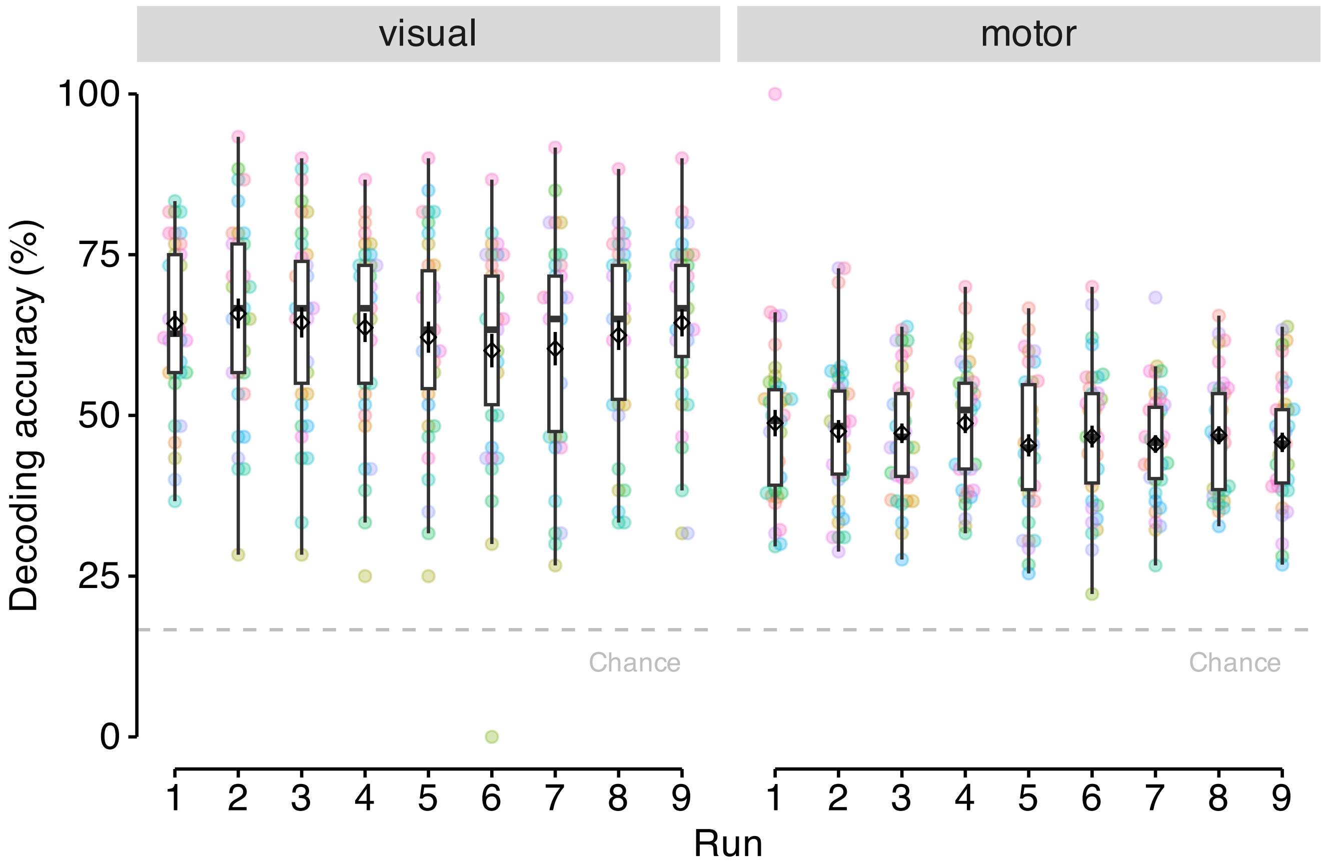

Fig. S14. Classification accuracy for each run of single trials. Cross-validated classification accuracy (in %) in decoding six unique visual objects in occipito-temporal data ("visual"; left panel) and six unique motor responses in sensorimotor cortex data ("motor"; right panel) during task performance on single trials, separately for each task run (x-axis). Chance level for each run is at 16.67% (horizontal dashed line). Boxplots indicate the median and IQR. The lower and upper hinges correspond to the first and third quartiles (the 25th and 75th percentiles). The upper whisker extends from the hinge to the largest value no further than $1.5*$ IQR from the hinge (where IQR is the inter-quartile range, or distance between the first and third quartiles). The lower whisker extends from the hinge to the smallest value at most $1.5*$ IQR of the hinge. The diamond shapes show the sample mean. Error bars indicate ±1 SEM. Each dot corresponds to averaged data from one participant. All statistics have been derived from data of $n = 39$ human participants who participated in one experiment.

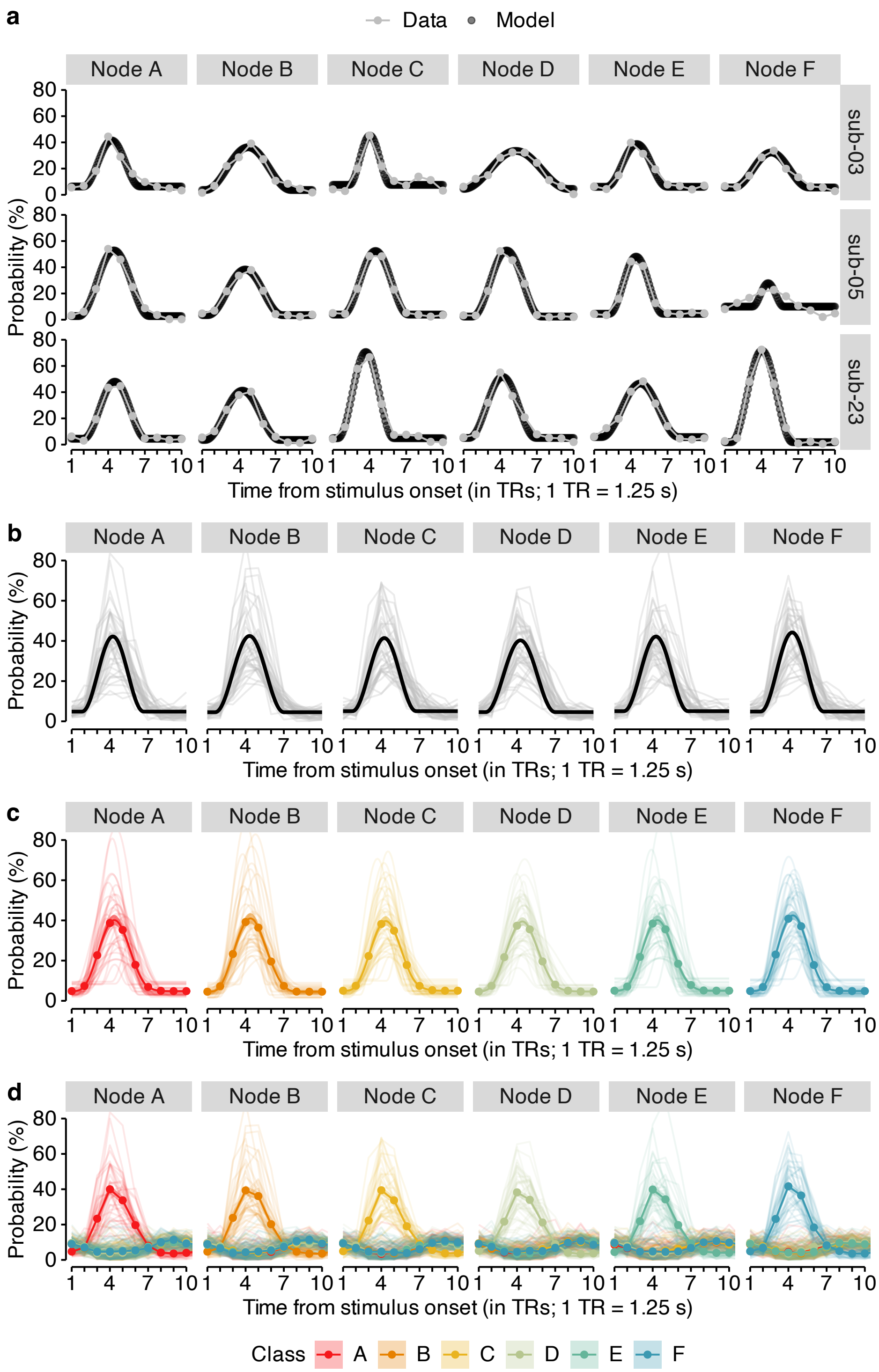

Fig. S15. Individual fits of sine wave response function to probabilistic classifier evidence. (a) Time courses (in TRs from stimulus onset; x-axis) of probabilistic classifier evidence (in %; y-axis) generated by the sine wave response function with fitted parameters (black dotted line) or the true data (gray line and dots) separately for the six stimulus classes (vertical panels) and three randomly chosen example participants (horizontal panels). **(b)** Time courses (in TRs from stimulus onset; x-axis) of mean probabilistic classifier evidence (in %; y-axis) averaged separately for each participant (gray semi-transparent lines) and each of the six stimulus class (vertical panels) or predicted by the sine wave response model based on fitted parameters averaged across all participants (black line). **(c)** Time courses (in TRs from stimulus onset; x-axis) of mean probabilistic classifier evidence (in %; y-axis) as predicted by the sine wave response model based on fitted parameters derived separately for each participant (individual, semi-transparent lines). **(d)** Time courses (in TRs from stimulus onset; x-axis) of mean probabilistic classifier evidence (in %; y-axis) for each of the six stimulus class (colors), separately for each of the stimulus trial type (vertical panels). Each semi-transparent line in (b), (c) and (d) represents data from one participant. Classifier probabilities in (a), (b), (c) and (d) were normalized across 15 TRs. The chance level therefore is at $100/15 = 6.67\%$. 1 TR = 1.25 s. All statistics have been derived from data of $n = 39$ human participants who participated in one experiment.

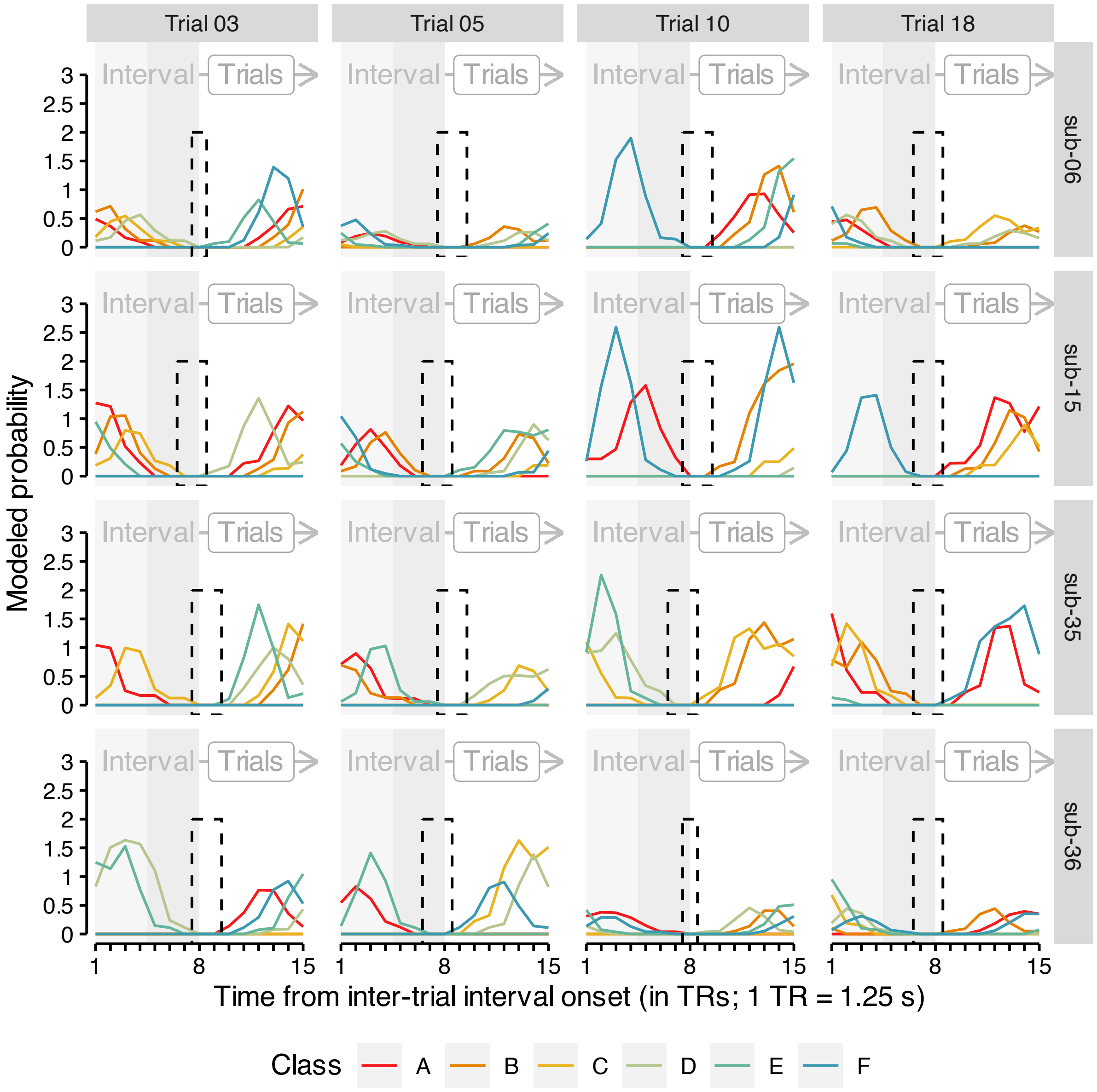

Fig. S16. Illustration of modeled stimulus-driven activity during on-task intervals. Time courses (in TR from ITI onset; x-axis; light and dark gray background) of modeled probabilistic classifier evidence (in %; y-axis) based on the sine wave response function with individually fitted parameters separately for the six stimulus classes (colors; see legend), four randomly chosen example participants (horizontal panels) and four randomly chosen example trials (vertical panels). Each line in represents data for one stimulus class from one participant on a particular trial. Rectangles with dashed lines illustrate TR intervals with no expected stimulus-driven activity (based on the modeling approach). 1 TR = 1.25 s. Source data are provided as a Source Data file.

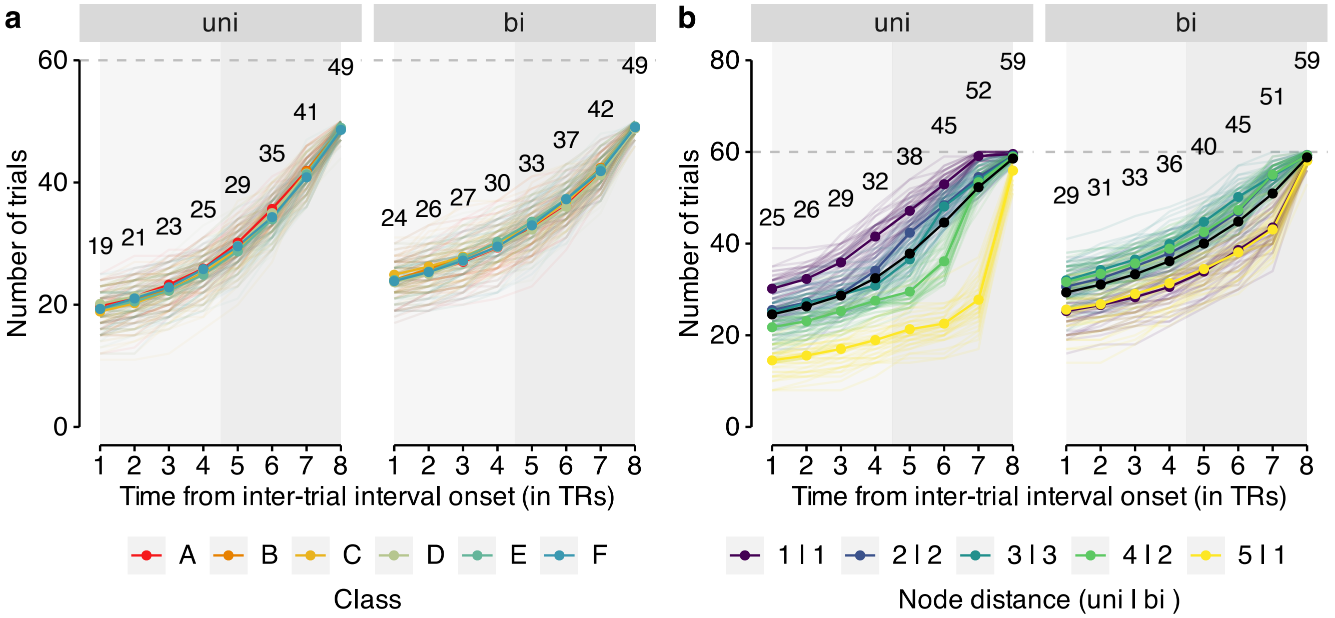

Fig. S17. Analysis of classifier probabilities in subsets of sequence trials after removing data points expected to contain stimulus-evoked activity. (a) Number of trials (y-axis) for each TR from ITI onset (x-axis; light and dark gray background) separately for each of the six stimulus classes (colors; see legend) and both graph structures (`uni` and `bi`; vertical panels). **(b)** As in (a), but for each graph node distance. The black line represents the mean number of trials across classes of node distances. The maximum number of trials for each stimulus class (a) or node distance (b) is 60 (horizontal dashed gray line). Each semi-transparent line represents data from one participant. Numbers indicate mean number of trials across all participants and stimulus classes (a) or node distances (b). All data are from the occipito-temporal anatomical ROIs. Qualitatively similar figures are obtained when using data from the motor ROI. All statistics have been derived from data of $n = 39$ human participants who participated in one experiment. 1 TR = 1.25 s.

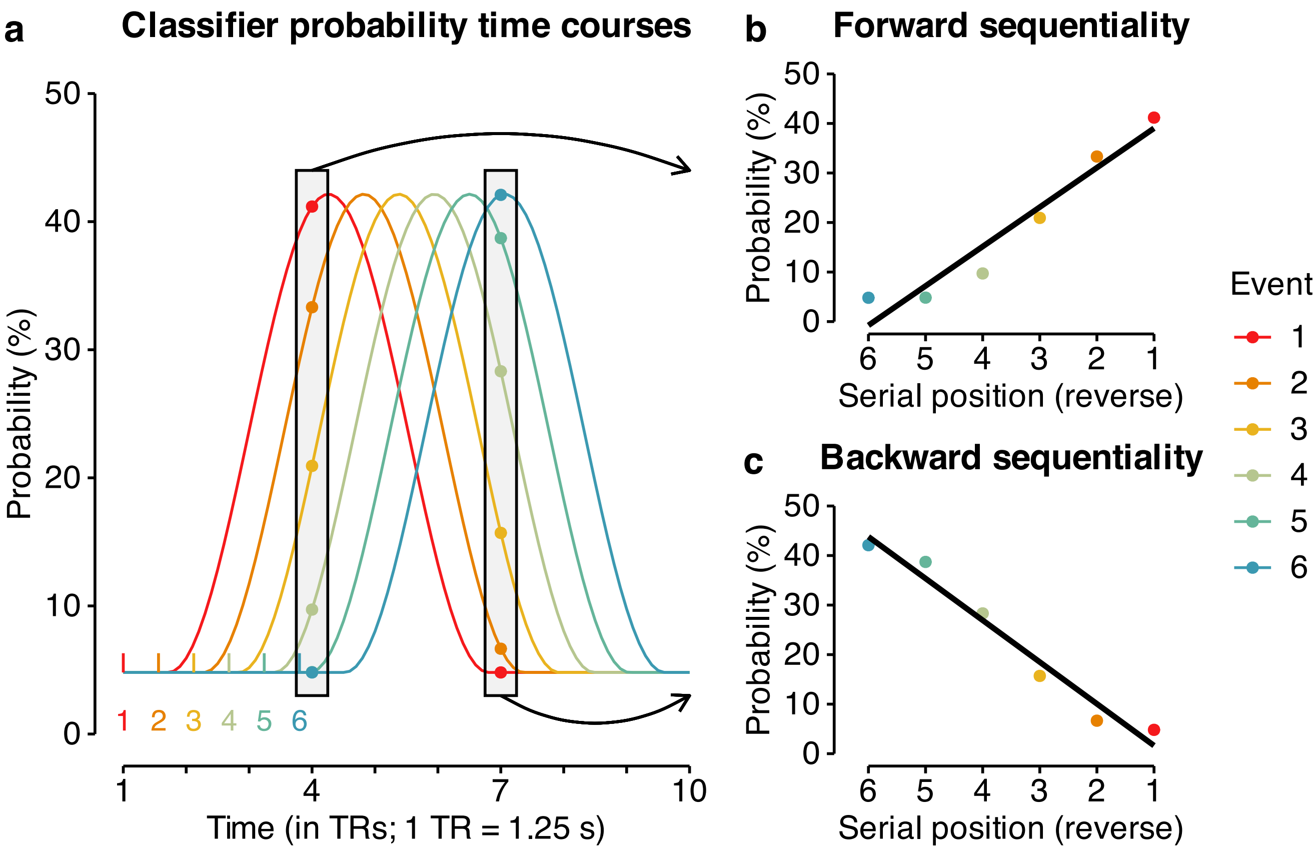

Fig. S18. Illustration of replay sequentiality metric in fMRI classifier time courses. (a) Illustrated time courses (in TRs; x-axis) of probabilistic classification evidence (in %; y-axis) for six (replay) sequence events (colors; see legend), as in the current study design. Classifier probabilities were derived from the same sine-based response function model used to fit classifier time courses on single trials. The delay between event onsets was set to 700 ms, for illustrative purposes only. Note, that when considering the ordering of classifier probabilities within a single TR (rectangles), the same event sequence (events 1 to 6) elicits both forward and backward sequentiality in earlier and later TR, respectively. **(b)** Forward sequentiality is indicated by a positive slope of a linear regression that relates the serial position of sequence events to their classifier probabilities within a single TR. Note, that the x-axis is reversed (or value for the slope sign-flipped) so that positive values indicate forward sequentiality. **(c)** Backward sequentiality is indicated by a positive slope of a linear regression that relates the serial position of sequence events to their classifier probabilities within a single TR. Note, that the x-axis is reversed (or value for the slope sign-flipped) so that negative values indicate backward sequentiality. Forward replay would thus be indicated by forward sequentiality in earlier TRs and backward sequentiality in later TRs. Conversely, backward replay would be indicated by backward sequentiality in earlier TRs and forward sequentiality in later TRs. For further details and an empirical investigation of this analysis approach, see (17).

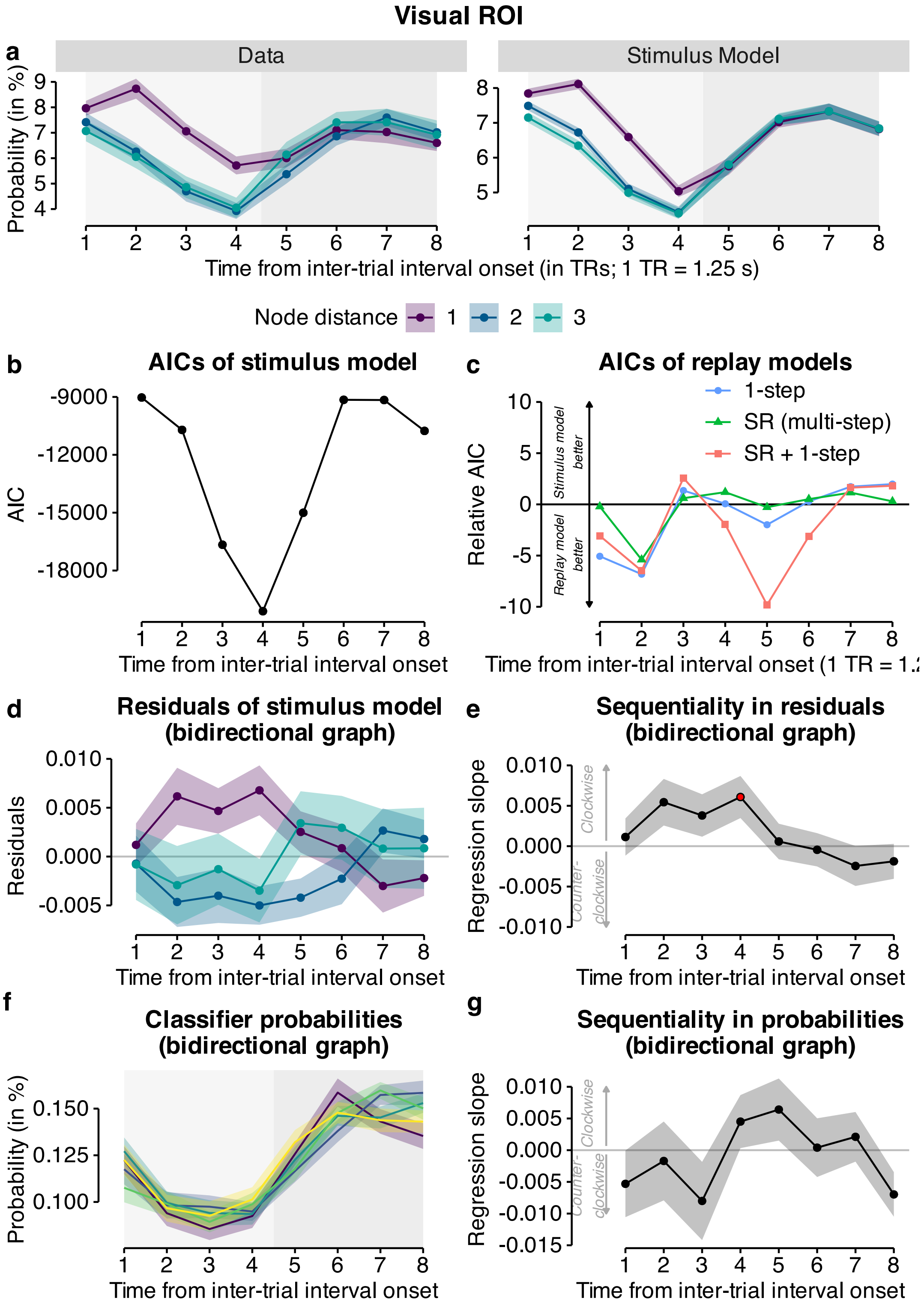

Fig. S19. Classifier probabilities during on-task intervals of sequence trials on bidirectional graph trials. For details on each panel, see main text Fig. 5.

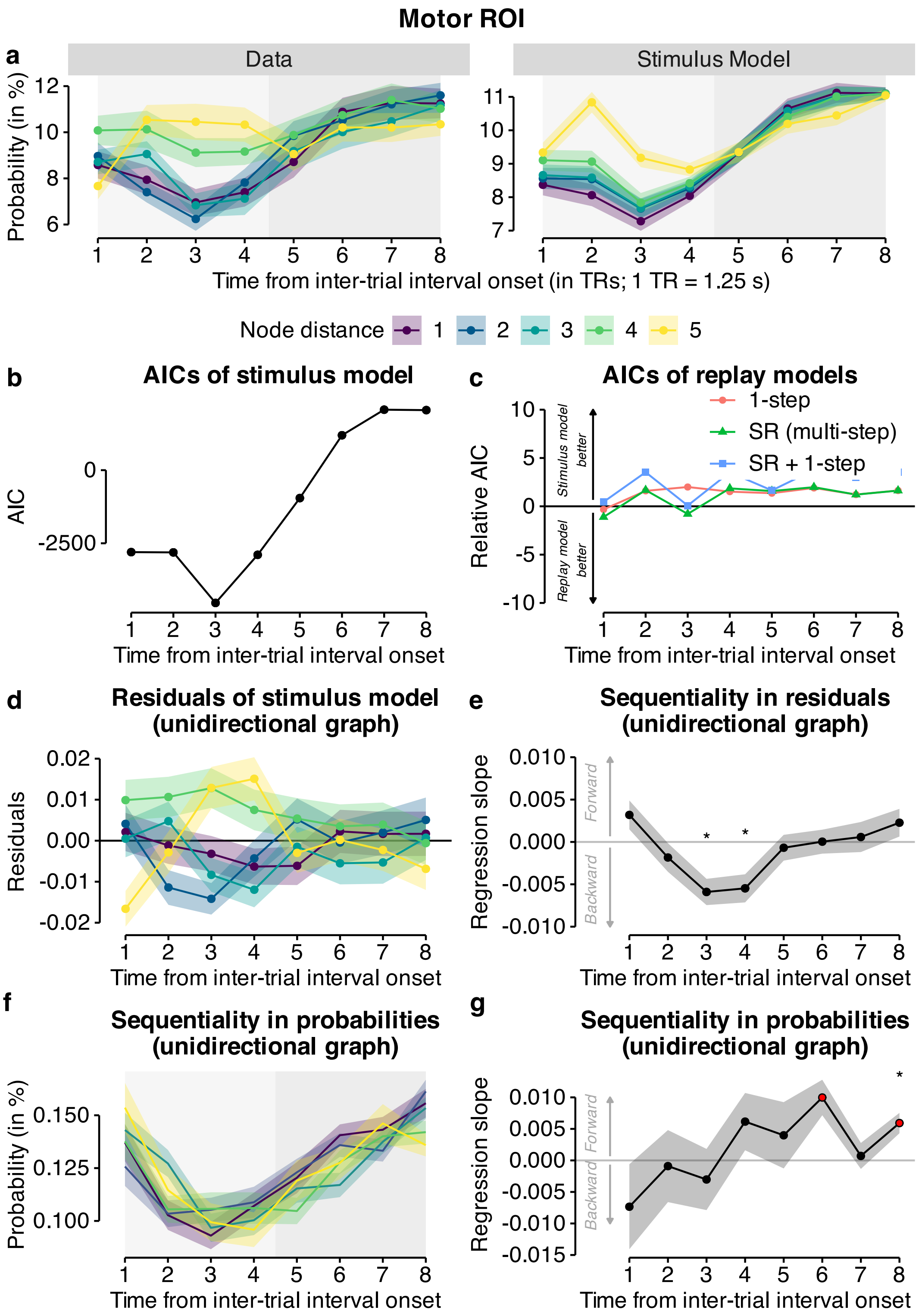

Fig. S20. Classifier probabilities during on-task intervals in motor cortex. For details on each panel, see main text Fig. 5.

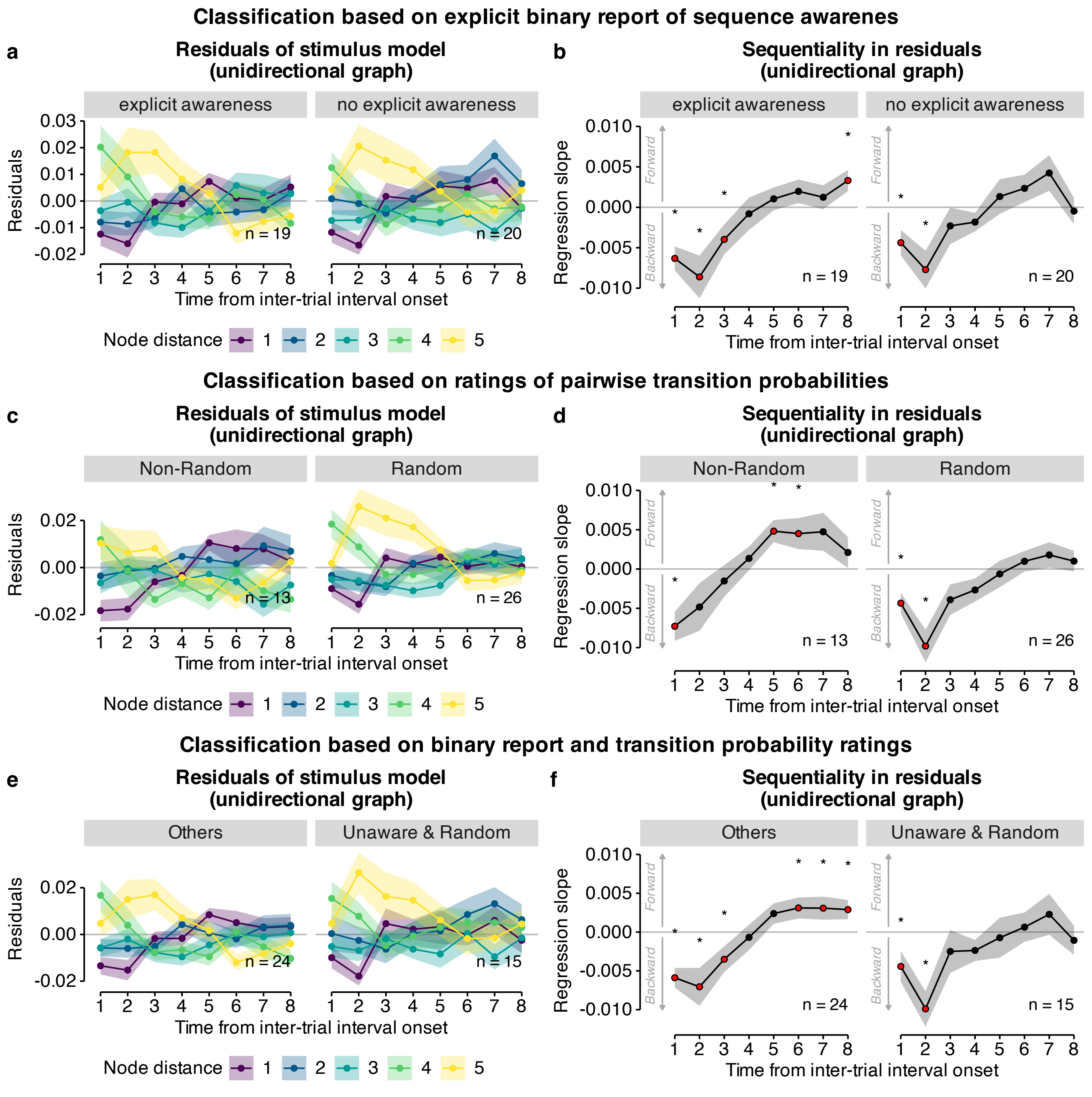

Fig. S21. Influence of sequence awareness on evidence for sequential neural replay. (a) Time courses (in TRs from ITI onset; x-axis) of the residuals of the stimulus model for each of the five node distances in unidirectional graph data (colors; see legend), split by self-reported sequence awareness (panels). **(b)** Time courses (in TRs from ITI onset; x-axis) of mean regression slopes (y-axis) relating node distance (i.e., sequential position from current node in the graph structure) to their residuals in the stimulus model, split by self-reported sequence awareness (panels). Positive and negative values indicate forward and backward sequentiality, respectively. Red dots and asterisks indicate significant differences from baseline (horizontal gray line at zero; all $ps \leq .05$, uncorrected; two-sided one-sample t-tests, one test per TR). **(c)** As in (a), split by classification of transition probability ratings based on a random guessing model. **(d)** As in (b), split by classification of transition probability ratings as in (c). **(e)** As in (a), split by a combination of binary report of sequence awareness and classification of transition probability ratings. **(f)** As in (b), split as in (e).

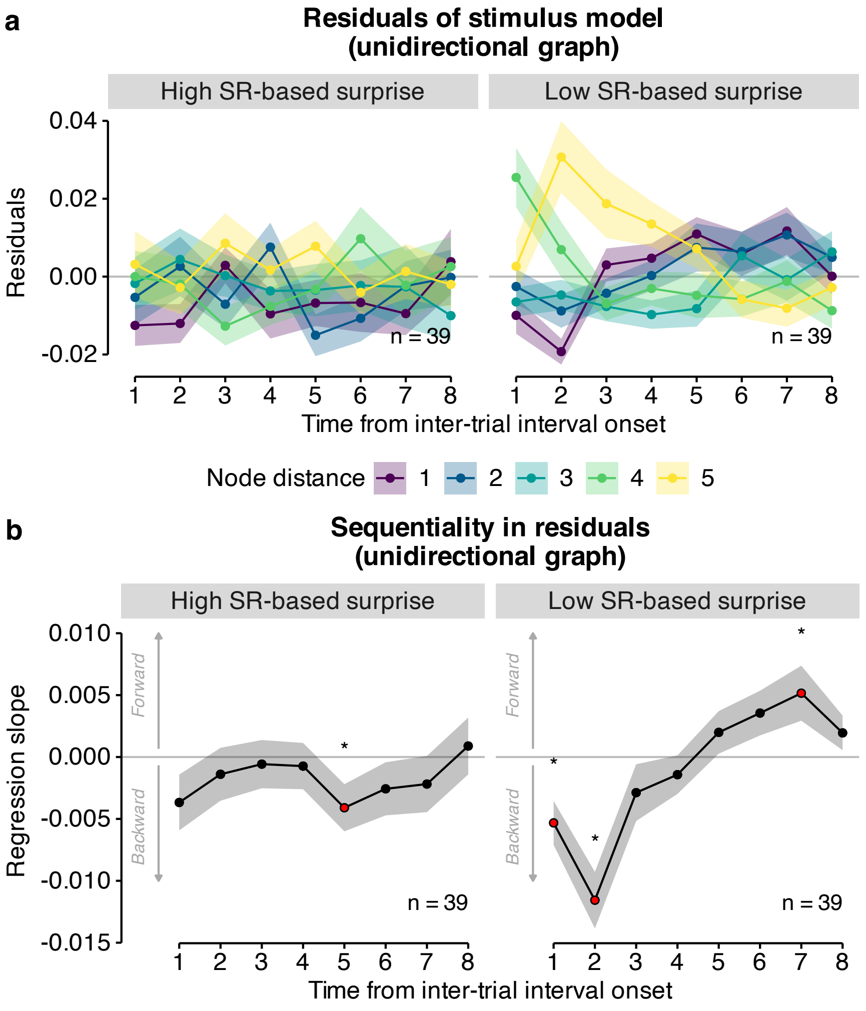

Fig. S22. Relationship between SR-based surprise and evidence for sequential neural replay. (a) Time courses (in TRs from ITI onset; x-axis) of the residuals of the stimulus model for each of the five node distances in unidirectional graph data (colors; see legend), separately for trials with high or low SR-based surprise (panels). **(b)** Time courses (in TRs from ITIs onset; x-axis) of mean regression slopes (y-axis) relating node distance (i.e., sequential position from current node in the graph structure) to their residuals in the stimulus model, separately for trials with high or low SR-based surprise (panels). Positive and negative values indicate forward and backward sequentiality, respectively. Red dots and asterisks indicate significant differences from baseline (horizontal gray line at zero; all ps $\leq .05$, uncorrected; two-sided one-sample t-tests, one test per TR). All statistics have been derived from data of $n = 39$ human participants who participated in one experiment.

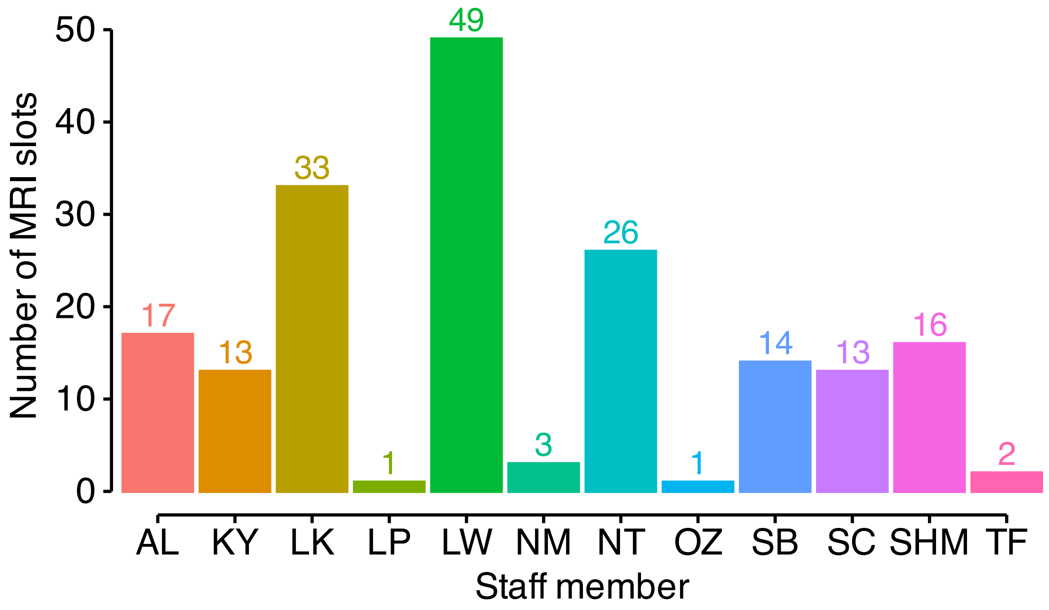

Fig. S23. True team effort during fMRI data collection. Number of MRI slots (y-axis and text labels) collected by each study staff member (x-axis; initials), indicating a a real team effort during data collection.

Table S1. Labels used to index brain regions to create participant-specific anatomical masks of selected ROIs based on Freesurfer's recon-all labels (42)

ROI	Freesurfer labels (brain region)
Occipito-temporal	1005, 2005 (cuneus); 1011, 2011 (lateral occipital sulcus); 1021, 2021 (pericalcarine gyrus); 1029, 2029 (superio parietal lobule); 1013, 2013 (lingual gyrus); 1008, 2008 (inferior parietal lobule); 1007, 2007 (fusiform gyrus); 1009, 2009 (inferior temporal gyrus); 1016, 2016 (parahippocampal gyrus); 1015, 2015 (middle temporal gyrus)
Hippocampus	17, 53 (left and right hippocampus)
Motor	1024, 2024 (left and right gyrus precentralis); 1022, 2022 (left and right gyrus postcentralis)

References

1. F Arcediano, M Escobar, RR Miller, Bidirectional associations in humans and rats. *J. Exp. Psychol. Animal Behav. Process.* **31**, 301–318 (2005).
2. PB Sharp, E Eldar, Humans adaptively deploy forward and backward prediction. *Nat. Hum. Behav.* **8**, 1726–1737 (2024).
3. CE Shannon, A mathematical theory of communication. *The Bell Syst. Tech. J.* **27**, 379–423 (1948).
4. NW Schuck, Y Niv, Sequential replay of nonspatial task states in the human hippocampus. *Science* **364**, eaaw5181 (2019).
5. I Momennejad, AR Otto, ND Daw, KA Norman, Offline replay supports planning in human reinforcement learning. *eLife* **7**, e32548 (2018).
6. A Tambini, L Davachi, Persistence of hippocampal multivoxel patterns into postencoding rest is related to memory. *Proc. Natl. Acad. Sci.* **110**, 19591–19596 (2013).
7. B Rossion, G Pourtois, Revisiting Snodgrass and Vanderwart's object pictorial set: The role of surface detail in basic-level object recognition. *Perception* **33**, 217–236 (2004).
8. JG Snodgrass, M Vanderwart, A standardized set of 260 pictures: norms for name agreement, image agreement, familiarity, and visual complexity. *J. Exp. Psychol. Hum. learning memory* **6**, 174—215 (1980).
9. MM Garvert, RJ Dolan, TE Behrens, A map of abstract relational knowledge in the human hippocampal–entorhinal cortex. *eLife* **6** (2017).
10. Y Liu, MG Mattar, TEJ Behrens, ND Daw, RJ Dolan, Experience replay is associated with efficient nonlocal learning. *Science* **372** (2021).
11. JW Peirce, PsychoPy—psychophysics software in python. *J. Neurosci. Methods* **162**, 8–13 (2007).
12. JW Peirce, Generating stimuli for neuroscience using PsychoPy. *Front. Neuroinformatics* **2** (2008).
13. J Peirce, et al., Psychopy2: Experiments in behavior made easy. *Behav. Res. Methods* **51**, 195–203 (2019).
14. P Virtanen, et al., Scipy 1.0: fundamental algorithms for scientific computing in python. *Nat. Methods* (2020).
15. G Van Rossum, FL Drake, *Python 3 Reference Manual.* (CreateSpace, Scotts Valley, CA), (2009).
16. W Yu, A Zadbood, AJH Chanales, L Davachi, Repetition dynamically and rapidly increases cortical, but not hippocampal, offline reactivation. *Proc. Natl. Acad. Sci.* **121**, e2405929121 (2024).
17. L Wittkuhn, NW Schuck, Dynamics of fMRI patterns reflect sub-second activation sequences and reveal replay in human visual cortex. *Nat. Commun.* **12** (2021).
18. N Weiskopf, C Hutton, O Josephs, R Deichmann, Optimal EPI parameters for reduction of susceptibility-induced BOLD sensitivity losses: A whole-brain analysis at 3 T and 1.5 T. *NeuroImage* **33**, 493–504 (2006).
19. O Esteban, et al., fMRIPrep: A robust preprocessing pipeline for functional MRI. *Nat. Methods* **16**, 111–116 (2018).
20. O Esteban, et al., Analysis of task-based functional MRI data preprocessed with fMRIPrep. *bioRxiv* (2019).
21. YO Halchenko, et al., datalad/datalad 0.11.5 (2019).
22. YO Halchenko, et al., DataLad: distributed system for joint management of code, data, and their relationship. *J. Open Source Softw.* **6**, 3262 (2021).
23. AS Wagner, et al., *The DataLad Handbook.* (Zenodo), (2020).
24. KJ Gorgolewski, et al., The brain imaging data structure, a format for organizing and describing outputs of neuroimaging experiments. *Sci. Data* **3** (2016).
25. M Visconti di Oleggio Castello, et al., Repronim/reproin 0.6.0 (2020).
26. GM Kurtzer, V Sochat, MW Bauer, Singularity: Scientific containers for mobility of compute. *PLoS ONE* **12**, e0177459 (2017).
27. VV Sochat, CJ Prybol, GM Kurtzer, Enhancing reproducibility in scientific computing: Metrics and registry for singularity containers. *PLoS ONE* **12**, e0188511 (2017).
28. X Li, PS Morgan, J Ashburner, J Smith, C Rorden, The first step for neuroimaging data analysis: Dicom to nifti conversion. *J. Neurosci. Methods* **264**, 47–56 (2016).
29. OF Gulban, et al., poldracklab/pydeface: v2.0.0 (2019).
30. KJ Gorgolewski, et al., Nipype: A flexible, lightweight and extensible neuroimaging data processing framework in Python. *Front. Neuroinformatics* **5** (2011).
31. KJ Gorgolewski, et al., Nipype (2019).
32. T Yarkoni, et al., PyBIDS: Python tools for BIDS datasets. *J. Open Source Softw.* **4**, 1294 (2019).
33. T Yarkoni, et al., bids-standard/pybids: 0.9.3 (2019).
34. C Gorgolewski, et al., bids-standard/bids-validator: 1.4.3 (2020).
35. O Esteban, et al., MRIQC: Advancing the automatic prediction of image quality in MRI from unseen sites. *PLoS ONE* **12**, e0184661 (2017).
36. O Esteban, et al., fMRIPrep 1.2.2. (2019).
37. A Abraham, et al., Machine learning for neuroimaging with scikit-learn. *Front. Neuroinformatics* **8** (2014).
38. NJ Tustison, et al., N4itk: Improved n3 bias correction. *IEEE Transactions on Med. Imaging* **29**, 1310–1320 (2010).
39. B Avants, C Epstein, M Grossman, J Gee, Symmetric diffeomorphic image registration with cross-correlation: Evaluating automated labeling of elderly and neurodegenerative brain. *Med. Image Analysis* **12**, 26–41 (2008).
40. Y Zhang, M Brady, S Smith, Segmentation of brain MR images through a hidden markov random field model and the expectation-maximization algorithm. *IEEE Transactions on Med. Imaging* **20**, 45–57 (2001).
41. M Reuter, HD Rosas, B Fischl, Highly accurate inverse consistent registration: A robust approach. *NeuroImage* **53**,

1181–1196 (2010).
42. AM Dale, B Fischl, MI Sereno, Cortical surface-based analysis. *NeuroImage* **9**, 179–194 (1999).
43. A Klein, et al., Mindboggling morphometry of human brains. *PLOS Comput. Biol.* **13**, e1005350 (2017).
44. AC Evans, AL Janke, DL Collins, S Baillet, Brain templates and atlases. *NeuroImage* **62**, 911–922 (2012).
45. V Fonov, A Evans, R McKinstry, C Almli, D Collins, Unbiased nonlinear average age-appropriate brain templates from birth to adulthood. *NeuroImage* **47**, S102 (2009).
46. RW Cox, JS Hyde, Software tools for analysis and visualization of fmri data. *NMR Biomed.* **10**, 171–178 (1997).
47. DN Greve, B Fischl, Accurate and robust brain image alignment using boundary-based registration. *NeuroImage* **48**, 63–72 (2009).
48. M Jenkinson, P Bannister, M Brady, S Smith, Improved optimization for the robust and accurate linear registration and motion correction of brain images. *NeuroImage* **17**, 825–841 (2002).
49. JD Power, et al., Methods to detect, characterize, and remove motion artifact in resting state fmri. *NeuroImage* **84**, 320–341 (2014).
50. Y Behzadi, K Restom, J Liau, TT Liu, A component based noise correction method (CompCor) for BOLD and perfusion based fMRI. *NeuroImage* **37**, 90–101 (2007).
51. TD Satterthwaite, et al., An improved framework for confound regression and filtering for control of motion artifact in the preprocessing of resting-state functional connectivity data. *NeuroImage* **64**, 240–256 (2013).
52. C Lanczos, Evaluation of noisy data. *J. Soc. for Ind. Appl. Math. Ser. B Numer. Analysis* **1**, 76–85 (1964).
53. SM Smith, JM Brady, SUSAN - a new approach to low level image processing. *Int. J. Comput. Vis.* **23**, 45–78 (1997).
54. F Pedregosa, et al., Scikit-learn: Machine learning in Python. *J. Mach. Learn. Res.* **12**, 2825–2830 (2011).
55. L Deuker, et al., Memory consolidation by replay of stimulus-specific neural activity. *J. Neurosci.* **33**, 19373–19383 (2013).
56. AM Dale, Optimal experimental design for event-related fmri. *Hum. Brain Mapp.* **8**, 109–114 (1999).
57. B Fischl, et al., Automatically parcellating the human cerebral cortex. *Cereb. Cortex* **14**, 11–22 (2004).
58. RA Poldrack, Region of interest analysis for fMRI. *Soc. Cogn. Affect. Neurosci.* **2**, 67–70 (2007).
59. JV Haxby, et al., Distributed and overlapping representations of faces and objects in ventral temporal cortex. *Science* **293**, 2425–2430 (2001).
60. L Kunz, L Deuker, H Zhang, N Axmacher, Chapter 26 - tracking human engrams using multivariate analysis techniques in *Handbook of in Vivo Neural Plasticity Techniques*, Handbook of Behavioral Neuroscience, ed. D Manahan-Vaughan. (Elsevier) Vol. 28, pp. 481–508 (2018).
61. D Bates, M Mächler, B Bolker, S Walker, Fitting linear mixed-effects models using lme4. *J. Stat. Softw.* **67**, 1–48 (2015).
62. R Core Team, R: A language and environment for statistical computing (2019).
63. DJ Barr, R Levy, C Scheepers, HJ Tily, Random effects structure for confirmatory hypothesis testing: Keep it maximal. *J. Mem. Lang.* **68**, 255–278 (2013).
64. MJD Powell, Developments of newuoa for unconstrained minimization without derivatives. *Dep. Appl. Math. Theor. Phys.* (2007).
65. MJD Powell, The bobyqa algorithm for bound constrained optimization without derivatives. *Dep. Appl. Math. Theor. Phys.* pp. 26–46 (2009).
66. JW Tukey, Comparing individual means in the analysis of variance. *Biometrics* **5**, 99–114 (1949).
67. R Lenth, emmeans: Estimated marginal means, aka least-squares means. *CRAN* (2019) R package version 1.3.4.
68. Y Benjamini, Y Hochberg, Controlling the false discovery rate: A practical and powerful approach to multiple testing. *J. Royal Stat. Soc.* **57**, 289–300 (1995).
69. CE Bonferroni, Teoria statistica delle classi e calcolo delle probabilità. *Pubblicazioni del R Istituto Super. di Scienze Econ. e Commer. di Firenze* **8**, 3–62 (1936).
70. J Cohen, Statistical power analysis for the behavioral sciences. *Lawrence Erlbaum Assoc.* (1988).
71. P Dayan, Improving generalization for temporal difference learning: The successor representation. *Neural Comput.* **5**, 613–624 (1993).
72. EM Russek, I Momennejad, MM Botvinick, SJ Gershman, ND Daw, Predictive representations can link model-based reinforcement learning to model-free mechanisms. *PLoS Comput. Biol.* **13**, e1005768 (2017).
73. S Hall-McMaster, P Dayan, NW Schuck, Control over patch encounters changes foraging behavior. *iScience* **24**, 103005 (2021).
74. C Koch, O Zika, NW Schuck, Influence of surprise on reinforcement learning in younger and older adults. *PsyArXiv* (2022).
75. JM Gablonsky, CT Kelley, A locally-biased form of the direct algorithm. *J. Glob. Optim.* **21**, 27–37 (2001).
76. SG Johnson, The nlopt nonlinear-optimization package. *The Compr. R Arch. Netw.* (2019).
77. RC Wilson, AG Collins, Ten simple rules for the computational modeling of behavioral data. *eLife* **8** (2019).
78. C Koch, O Zika, R Bruckner, NW Schuck, Influence of surprise on reinforcement learning in younger and older adults. *PLOS Comput. Biol.* **20**, 1–25 (2024).
79. MJD Powell, A direct search optimization method that models the objective and constraint functions by linear interpolation in *Advances in optimization and numerical analysis*, eds. S Gomez, JP Hennart. (Springer), pp. 51–67 (1994).
80. G Aguirre, E Zarahn, M D'Esposito, The variability of human, BOLD hemodynamic responses. *NeuroImage* **8**, 360 – 369 (1998).
